# BLUPmrMLM: A Fast mrMLM Algorithm in Genome-wide Association Studies

**DOI:** 10.1093/gpbjnl/qzae020

**Published:** 2024-02-29

**Authors:** Hong-Fu Li, Jing-Tian Wang, Qiong Zhao, Yuan-Ming Zhang

**Affiliations:** College of Plant Science and Technology, Huazhong Agricultural University, Wuhan 430070, China; College of Plant Science and Technology, Huazhong Agricultural University, Wuhan 430070, China; College of Plant Science and Technology, Huazhong Agricultural University, Wuhan 430070, China; College of Plant Science and Technology, Huazhong Agricultural University, Wuhan 430070, China

**Keywords:** Genome-wide association study, BLUP, Multilocus model, mrMLM, Large-scale dataset

## Abstract

Multilocus genome-wide association study has become the state-of-the-art tool for dissecting the genetic architecture of complex and multiomic traits. However, most existing multilocus methods require relatively long computational time when analyzing large datasets. To address this issue, in this study, we proposed a fast mrMLM method, namely, best linear unbiased prediction multilocus random-SNP-effect mixed linear model (BLUPmrMLM). First, genome-wide single-marker scanning in mrMLM was replaced by vectorized Wald tests based on the best linear unbiased prediction (BLUP) values of marker effects and their variances in BLUPmrMLM. Then, adaptive best subset selection (ABESS) was used to identify potentially associated markers on each chromosome to reduce computational time when estimating marker effects via empirical Bayes. Finally, shared memory and parallel computing schemes were used to reduce the computational time. In simulation studies, BLUPmrMLM outperformed GEMMA, EMMAX, mrMLM, and FarmCPU as well as the control method (BLUPmrMLM with ABESS removed), in terms of computational time, power, accuracy for estimating quantitative trait nucleotide positions and effects, false positive rate, false discovery rate, false negative rate, and F_1_ score. In the reanalysis of two large rice datasets, BLUPmrMLM significantly reduced the computational time and identified more previously reported genes, compared with the aforementioned methods. This study provides an excellent multilocus model method for the analysis of large-scale and multiomic datasets. The software mrMLM v5.1 is available at BioCode (https://ngdc.cncb.ac.cn/biocode/tool/BT007388) or GitHub (https://github.com/YuanmingZhang65/mrMLM).

## Introduction

Genome-wide association studies (GWAS) have become a standard method for dissecting the genetic architecture of complex and omics-related traits in animals, plants, and humans. GWAS focus on testing the associations of genome-wide markers with trait phenotypes of interest to identify genes that control complex and omics traits [1,2].

The mixed linear model (MLM) methodology has been widely used in GWAS since the inception of the MLM methodology [3–5], due to its effective control of important confounders (*e.g.*, population structure and relatedness). To reduce computational burden and further improve statistical power, a number of fast and approximate/exact algorithms have been proposed, such as EMMAX [6], CMLM [7], GEMMA [8], FaST-LMM [9], GRAMMAR-gamma [10], Bolt-LMM [11], fastGWA [12], and REGENIE [13]. Although these single-locus methods involve Bonferroni correction, they are simple, readily applicable, and computationally inexpensive [14,15]. In real data analysis, these methods may be unsuitable for revealing the genetic architecture of complex traits. In statistics, single-locus methods may miss true loci due to strict significance thresholds [16–18], which may result in missing heritability [19,20]. In genetics, most complex traits are controlled by a few large-effect genes and numerous small genes according to previous studies [21]. In addition, single-locus methods do not account for the effects of other SNPs and never fit a true genetic model of complex traits. Clearly, the explicit use of multiple loci in a model is a better alternative [17,22–24].

The traditional multilocus approach involves multiple regression, which fails when the number of markers is greater than the sample size and when there is multicollinearity between these markers. To address these problems, a considerable number of alternative methods, such as Bayesian lasso [25], ridge regression [26], lasso penalized logistic regression [27], adaptive lasso [28], elastic net [14], and empirical Bayes [29], have been developed. Although these methods have been shown to outperform single-locus methods, most of them fail when the number of markers is very large [30,31]. A more powerful alternative is to combine single-locus scanning with a multilocus model, such as the mrMLM [17].

In multilocus methods, MLMM [24] uses forward and backward procedures, while FarmCPU [32] splits MLMM into a fixed-effect model and a random-effect model and uses them iteratively. However, both still use the Bonferroni correction threshold, which limits their power to some extent [18]. To better balance high power and a low false positive rate (FPR) in quantitative trait nucleotide (QTN) detection, a number of multilocus methods have been proposed in our mrMLM software [33], such as mrMLM [17], ISIS EM-BLASSO [30], FASTmrEMMA [31], pLARmEB [34], and pKWmEB [35], in which a less stringent significance criterion replaces the overly conservative Bonferroni correction. Previous studies [18,33] have shown that these methods have high power, low FPR, and high accuracy. Although large datasets provide new opportunities for new discoveries, they also present enormous computational challenges.

To address the aforementioned problem, we proposed a new method, namely, the best linear unbiased prediction multilocus random-SNP-effect mixed linear model (BLUPmrMLM). In BLUPmrMLM, vectorized Wald tests were used to replace genome-wide scanning in mrMLM [36–39], and adaptive best subset selection (ABESS) [40] was used to select potentially associated markers on each chromosome. To increase power, residual error was used to fit unselected SNPs to identify additional suggested or significant QTNs [30]. In addition, shared memory and parallel computing schemes were used to reduce the running time. Our new method was validated via simulated and real data analyses. The results showed that BLUPmrMLM outperformed the other methods in terms of computational time, statistical power, accuracy, FPR, false discovery rate (FDR), false negative rate (FNR), F_1_ score, and receiver operating characteristic (ROC) curve.

## Method

### Association mapping populations in rice

#### The dataset of 1439 rice hybrids

The 1439 *indica* hybrids were genotyped using 1,098,527 SNPs (http://www.ncgr.ac.cn/RiceHap4) and phenotyped for heading date (HD) and grain length (GL) in Hangzhou and yield per plant (Yield), grain number (GN), and thousand grain weight (TGW) in Sanya (https://www.nature.com/articles/ncomms7258/), China [41]. The population structure was indicated by two principal components (PCs) from 1,098,527 SNP markers [41]. The rice reference genome used in this study was IRGSP 4.0 [41].

#### The 3K rice dataset

There were 2261 varieties genotyped by 1,011,601 SNPs, which were downloaded from the rice SNP-seek database website (https://snp-seek.irri.org/) and phenotyped by the grain length width ratio (GLWR) and TGW (https://www.rmbreeding.cn/phenotype) [42,43]. The numbers of varieties with phenotypes were 2013 and 1318 for GLWR and TGW, respectively. The population structure was indicated by the top 4 PCs of 1,011,601 SNP markers [43]. The rice reference genome used in this study was IRGSP 1.0 [43].

All the rice genes were obtained from http://www.ricedata.cn/ and confirmed via transgenic experiments in the references provided.

### Monte Carlo simulation studies

All the simulation experiments were similar to those in our previous studies [17,31]. In all the simulations, 300 individuals each with 50,000 SNP markers, as shown in Table S1, were sampled from a real Simmental beef cattle dataset [44]. To investigate the performance of BLUPmrMLM under different polygenic backgrounds, four simulation datasets were simulated, and the number of replicates in each dataset was 1000.

In the first simulation dataset, one pair of QTNs was simulated and placed on each of the first five chromosomes (1 to 5). To investigate the effect of closely linked QTNs on the performance of BLUPmrMLM, two pairs of closely linked QTNs were simulated on chromosomes 1 and 5. QTN1 and QTN2 on chromosome 1 were simulated as positive effects, while QTN9 and QTN10 on chromosome 5 were simulated as negative and positive effects, respectively. Their sizes (*r*^2^), positions, and effects are listed in Table S2. The average μ and residual variance $\sigma_{e}^{2}$ were set to 10.0. Phenotypic values y were simulated from Equation 1:

(1) $$y=\mu +\sum_{k=1}^{10}X_{k}\beta_{k}+\varepsilon$$

where $\beta_{k}$ is the effect for the *k*-th QTN, $X_{k}$ is the design matrix for $\beta_{k}$, and the residual errors are $\varepsilon ∼{\text{MVN}}_{n}(0,\sigma_{e}^{2}I_{n})$.

To investigate the effect of an additive polygenic background on BLUPmrMLM, the polygenic effect was simulated in the second simulation dataset by ${\text{MVN}}_{n}(0,\sigma_{g}^{2}K_{g})$, where $\sigma_{g}^{2}=2.00$ is polygenic variance, $h_{g}^{2}=8.33\%$, and $K_{g}$ is the kinship matrix between a pair of lines. The QTN size (*r*^2^, %), QTN position, residual variance, and population mean were the same as those in the first simulation dataset. Phenotypes were simulated from Equation 2:

(2) $$y=\mu +\sum_{k=1}^{10}X_{k}\beta_{k}+\xi +\varepsilon$$

where $\xi ∼{\text{MVN}}_{n}(0,\sigma_{g}^{2}K_{g})$, and the meanings of the other symbols are consistent with those in the first simulated dataset.

To investigate the effect of epistatic polygenic background on BLUPmrMLM, five pairs of epistatic QTNs with 2% heritability each were simulated in the third simulation dataset by ${\text{MVN}}_{n}(0,\sigma_{ep}^{2}K_{ep})$, where $\sigma_{ep}^{2}$ is epistatic variance. All the parameters for five pairs of epistatic QTNs are reported in Table S3. The QTN size (*r*^2^, %), QTN position, residual variance, and population mean were the same as those in the first simulation dataset. Phenotypes were simulated from Equation 3:

(3) $$y=\mu +\sum_{k=1}^{10}X_{k}\beta_{k}+\sum_{j=1}^{5}(A_{j}\#B_{j})\beta_{jj}+\varepsilon$$

where $\beta_{jj}$ is the epistatic effect, and $A_{j}\#B_{j}$ is the corresponding incidence coefficient. The meanings of the other symbols are as described above.

To investigate the effect of additive and epistatic polygenic backgrounds on BLUPmrMLM, additive and epistatic polygenic effects were simulated in the fourth simulation dataset by ${\text{MVN}}_{n}(0,2.0\times K_{g})$ and five pairs of epistatic QTNs with 2% heritability each, as described in the second and third simulation experiments, respectively. The other parameters were the same as those in the first simulation experiment. Phenotypes were simulated from Equation 4:

(4) $$y=\mu +\sum_{k=1}^{10}X_{k}\beta_{k}+\sum_{j=1}^{5}(A_{j}\#B_{j})\beta_{jj}+\xi +\varepsilon$$

where the meanings of all the symbols are as described above.

The empirical statistical power of each QTN was calculated as the proportion of samples with logarithm of odds (LOD) ≥ 3.0 for BLUPmrMLM, mrMLM, and the control, and *P* ≤ 1.00E−6 (0.05/*m*) was used for the other methods. A QTN detected within 2 kb of its simulated QTN position was considered to be a true QTN. The FPR was the ratio of the number of false QTNs detected to the total number of zero effects in the full model, while the FNR was the ratio of the number of simulated QTNs not detected to the total number of nonzero effects in the full model. The mean squared error (MSE) and mean absolute deviation (MAD) were used to assess the accuracy of the estimates of QTN positions and effects, where the MSE and MAD for the *i*-th QTN parameter $\beta_{i}$ were calculated as follows:

(5) $$MSE_{i}=\frac{1}{n_{T}}\sum_{j=1}^{n_{T}}{\left({\hat{\beta }}_{ij}-\beta_{i}\right)}^{2}MAD_{i}=\frac{1}{n_{T}}\sum_{j=1}^{n_{T}}\left|{\hat{\beta }}_{ij}-\beta_{i}\right|$$

where $n_{T}$ is the number of replicates, ${\hat{\beta }}_{ij}$ is the estimate for the *i*-th QTN parameter in the *j*-th sample, and $\beta_{i}$ is the true value of the *i*-th QTN parameter. The method with a small MSE (or MAD) is better than the method with a large MSE (or MAD).

### Selection of potentially associated markers in BLUPmrMLM

#### Genetic model

As described by Gualdrón Duarte et al. [36], Ning et al. [37], Wang et al. [38], and Wang et al. [39], the phenotypes for quantitative traits, **y** , were indicated by the following MLM:

(6) y=Xβ+Zγ+ε

where y is a n×1 vector of phenotypic values for quantitative trait; *n* is the number of individuals in the association mapping population; β is a q×1 vector of fixed effects, including the population mean; X is the design matrix for β; $\gamma ∼{\text{MVN}}_{m}(0,I_{m}\sigma_{g}^{2})$ is a m×1 vector of random effects for all *m* markers; $\sigma_{g}^{2}$ is the genetic variance; Z is the n×m design matrix for γ; residual errors $\varepsilon ∼{\text{MVN}}_{n}(0,I_{n}\sigma_{}{}^{2})$ is a n×1 vector; $\sigma^{2}$ is residual variance; and I is the identity matrix. Thus, the variance of y in Equation 1 is expressed as:

(7) $$V=\text{Var}(y)=ZZ^{T}\sigma_{g}^{2}+I_{n}\sigma_{}^{2}=K\sigma_{g}^{2}+I_{n}\sigma_{}^{2}$$

where $K=ZZ^{T}$ is a marker-inferred kinship matrix.

#### Parameter estimation for genetic and residual variances

The genetic and residual variances $\theta ={\left(\sigma_{g}^{2},\sigma_{}^{2}\right)}^{T}$ in Equation 6 are estimated by maximizing the restricted log-likelihood function (RELF):

(8) $$L=-\frac{1}{2}(\text{log}\;|V|+\text{log}\;|X^{T}V^{-1}X|+y^{T}Py)$$

where $P=V^{-1}-V^{-1}X{\left(X^{T}V^{-1}X\right)}^{-1}X^{T}V^{-1}$. Here, a lower computational demand and faster average information (AI) iterative algorithm were used to maximize the RELF via the following formula:

(9) $$\theta^{\left(t+1\right)}=\theta^{\left(t\right)}+{\left(AI^{\left(t\right)}\right)}^{-1}\frac{\partial L}{\partial \theta }|\theta^{\left(t\right)}$$

as described by Johnson & Thompson [45], where *t* is the number of iterations, **AI** is the average of the observed and expected information matrices, and $\frac{\partial L}{\partial \theta }$ is a vector of the first derivatives of the RELF with respect to θ:

(10) $$\hat{\theta }=\left(\begin{array}{c} {\hat{\sigma }}_{g}^{2}\\ {\hat{\sigma }}^{2} \end{array}\right),\;AI=\frac{1}{2}\left(\begin{array}{cc} y^{T}P\mathrm{KPKPy} & y^{T}P\mathrm{KPPy}\\ y^{T}P\mathrm{KPPy} & y^{T}P\mathrm{PPy} \end{array}\right),\;\frac{\partial L}{\partial \theta }=-\frac{1}{2}\left(\begin{array}{c} \text{tr}\left(PK\right)+y^{T}P\mathrm{KPy}\\ \text{tr}\left(P\right)+y^{T}PPy \end{array}\right)$$

Since the iterative AI algorithm is highly sensitive to the choice of initial values of variance parameters, the initial values of these parameters in the AI algorithm are determined via several iterations using expectation maximization (EM) algorithm, as described by Yang and colleagues [46]. The EM algorithm is expressed as:

(11) $$\left(\begin{array}{c} {\hat{\sigma }}_{g}^{2(t+1)}\\ {\hat{\sigma }}^{2(t+1)} \end{array}\right)=\frac{1}{n}\left(\begin{array}{c} {\hat{\sigma }}_{g}^{4(t)}y^{T}P\mathrm{KPy}+\text{tr}\left({\hat{\sigma }}_{g}^{2(t)}I-{\hat{\sigma }}_{g}^{4(t)}PK\right)\\ {\hat{\sigma }}^{4(t)}y^{T}PPy+\text{tr}\left({\hat{\sigma }}^{2(t)}I-{\hat{\sigma }}^{4(t)}P\right) \end{array}\right)$$

If the estimates of variance parameter θ do not change between the *t-*th and (*t*+1)-th iterations, the EM algorithm is used to implement the optimization for accelerating calculation via the Rcpp-based high-performance gaston package (https://cran.r-project.org/web/packages/gaston/).

#### Test statistics

As described by Henderson [47], the random-SNP-effect can be estimated by

(12) $$\hat{\gamma }=(I\sigma_{g}^{2})Z^{T}Py$$

and its corresponding variance is

(13) $$\text{Var}(\hat{\gamma })=(I\sigma_{g}^{2})Z^{T}P\mathrm{VPZ}(I\sigma_{g}^{2})$$

It should be noted that

(14) $$PVP=(V^{-1}-V^{-1}X{\left(X^{T}V^{-1}X\right)}^{-1}X^{T}V^{-1})\times V\left(V^{-1}-V^{-1}X{\left(X^{T}V^{-1}X\right)}^{-1}X^{T}V^{-1}\right)=P$$

If the number of markers is large, it takes a long time to compute the matrix $\text{Var}(\hat{\gamma })$. However, in the Wald tests for all the markers, only the diagonal elements of Var(γ) are affected. If these diagonal elements can be expressed as a vector, the Wald test can be implemented in a vectorial way, as shown in Equation 16. In this sense, genome scanning in mrMLM can be replaced by vectorized Wald tests in BLUPmrMLM. Using some matrix transformations and Equation 13, the diagonal element vector can be expressed in a simplified way as:

(15) $$\text{diag}\left(\text{Var}\left(\gamma \right)\right)=\left(\sigma_{g}^{4}\sum_{j}^{n}{\left(Z^{T}P\#Z^{T}\right)}_{ij}\right)$$

where # represents the hadamard product and $\sum_{j}^{n}{\left(Z^{T}P\#Z^{T}\right)}_{ij}$ is the row sum for $Z^{T}P\#Z^{T}$. Thus, vectorized Wald tests $W_{m\times 1}$ for all the *m* markers are denoted by

(16) $$W=\left({\hat{\gamma }}^{T}\;\#\;{\hat{\gamma }}^{T}\right)/\text{diag}\left(\text{Var}(\gamma )\right)∼\chi_{df=1}^{2}$$

for the null hypothesis $H_{0}:\gamma =0$. To save computational time, P and Py can be precomputed only once. Once the probabilities (*P* value) of the Wald tests are obtained, the markers with *P* ≤ 0.01 are selected and entered the next step.

#### ABESS optimal variable selection algorithm

If the number of markers selected in Wald tests is large, it will take a long time to estimate the effects via empirical Bayes. To overcome this problem, ABESS, developed by Zhu et al. [40], was used to further select markers on each chromosome. The procedure is as follows.

First, fixed effects in Equation 6 are estimated using $\hat{\beta }={\left(X^{T}V_{}^{-1}X\right)}^{-1}X^{T}V_{}^{-1}y$, so that the corrected phenotype used in ABESS is obtained by $y^{\prime }=y-X\hat{\beta }=Z\gamma +\varepsilon$.

Second, $y^{\prime }$ is used to further select markers on each chromosome via ABESS, implemented by the abess package (https://cran.r-project.org/web//packages/abess/).

### Identification of significant QTNs in BLUPmrMLM

#### Estimation of the effects of potential QTNs in a multilocus model

As described by Wang et al. [17] and Wen et al. [31], all the potentially associated markers obtained above are placed into one multilocus model:

(17) $$y=X\beta +\sum_{k=1}^{s}Z_{k}\gamma_{k}+\varepsilon$$

where **y**, **X**, β, and **ε** are the same as those in Equation 6; *s* is the number of potentially associated markers; $\gamma_{k}$ is the effect of the *k*-th marker; and **Z***_k_* is its corresponding incidence vector for $\gamma_{k}$. Here **X** includes the population average and population structure. All the priors and hyperparameters in Equation 17 are the same as those in the study by Wang and colleagues [48].

All the effects in Equation 17 are estimated by the EM empirical Bayesian (EMEB) method [49]. The procedure of EMEB is as follows.

1. Setting the initial values of all the parameters:
  (18) $$\begin{array}{l} \hat{\beta }={\left(X^{T}X\right)}^{-1}X^{T}y\\ {\hat{\sigma }}^{2}=\frac{1}{2n}{\left(y-X\beta \right)}^{T}\left(y-X\beta \right)\\ {\hat{\sigma }}_{k}^{2}={\left[{\left(Z_{k}^{T}Z_{k}\right)}^{-1}Z_{k}^{T}\left(y-X\beta \right)\right]}^{2}+{\left(Z_{k}^{T}Z_{k}\right)}^{-1}\sigma^{2} \end{array}$$
2. E step: the QTN effect is predicted by
  (19) $$E\left(\gamma_{k}\right)=\sigma_{k}^{2}Z_{k}^{T}V^{-1}\left(y-X\beta \right)$$
  where $V=\sum_{i=1}^{s}Z_{k}^{}Z_{k}^{T}\sigma_{k}^{2}+I_{n}\sigma^{2}$.
3. M step: update $\sigma_{k}^{2}$, $\hat{\beta }$, and ${\hat{\sigma }}^{2}$
  (20) $$\begin{array}{l} {\hat{\sigma }}_{k}^{2}=\frac{E\left(\gamma_{k}^{T}\gamma_{k}\right)+\omega }{\tau +3}\\ \hat{\beta }={\left(X^{T}V^{-1}X\right)}^{-1}X^{T}V^{-1}y\\ {\hat{\sigma }}^{2}=\frac{1}{n}{\left(y-X\beta \right)}^{T}\left[y-X\beta -\sum_{k=1}^{s}Z_{k}E\left(\gamma_{k}\right)\right] \end{array}$$
  where $\text{Var}\left(\gamma_{k}\right)=\sigma_{k}^{2}-\sigma_{k}^{2}Z_{k}^{T}V^{-1}Z_{k}\sigma_{k}^{2}$ and $E\left(\gamma_{k}^{T}\gamma_{k}\right)=E\left(\gamma_{k}^{T}\right)E\left(\gamma_{k}\right)+\text{tr}\left(\text{Var}\left(\gamma_{k}\right)\right)$.

Steps E and M are repeated until convergence is satisfied.

#### Identification of significant QTNs

If the absolute estimates of marker effects in Equation 17 are less than 1.00E−5, these markers are removed from Equation 17. We assumed that the number of markers remaining in the multilocus model was *t*. Thus, the likelihood ratio statistic can be used to identify significant QTNs

(21) $$\text{LOD}=0.4343\left[L_{1}\left(\theta_{1}\right)-L_{0}\left(\theta_{0}\right)\right]$$

for the null hypothesis $H_{0}:\;\gamma_{k}=0$, where $L_{1}\left(\theta_{1}\right)$ and $L_{0}\left(\theta_{0}\right)$ are the natural logarithms of likelihood functions under the full ($H_{0}:\;\gamma_{k}\neq 0$) and null ($H_{0}:\;\gamma_{k}=0$) models, respectively, $\theta_{0}=\left(\gamma_{1},\ldots ,\gamma_{k-1},\gamma_{k+1},\ldots ,\gamma_{t}\right)$ and $\theta_{1}=\left(\gamma_{1},\ldots ,\gamma_{t}\right)$.

Although the aforementioned multilocus model does not include multiple testing correction and *P* = 0.05 can be used as a threshold for significant QTNs, a more stringent significance threshold of LOD = 3.0 (*P* = 2.00E−4) was used in this study to more effectively control FPRs, as described in our previous multilocus methods [18].

To better fit the dataset, residual errors were used to fit the remaining potentially associated markers to identify additional significant QTNs, as described above.

### Mining of novel known and candidate genes in BLUPmrMLM

Once significant QTNs are obtained, bioinformatics, haplotype, and multiomics analyses are performed to mine known/candidate genes around these significant QTNs. Approximately 300 kb of each significant QTN was identified, and functional genes with evidence from transgenic experiments (https://www.ricedata.cn/ontology/default.aspx) and haplotype analysis were mined and further confirmed by the rice ATAC-seq dataset [50] (http://glab.hzau.edu.cn/RiceENCODE/).

### Implementation of BLUPmrMLM

We briefly describe how BLUPmrMLM is implemented in three steps.

First, all the potentially associated markers were selected from among all the markers in the genome using vectorized Wald tests, decollinearity, and ABESS. In vectorized Wald tests, a loose critical threshold of *P* = 0.01 is used by default to remove most markers that are not associated with the trait. Decollinearity removes closely linked markers near the peak, as described by Wang and colleagues [17]. In real data analysis, the region length is set to 20 kb, which can be artificially selected. In ABESS, potentially associated markers are selected on each chromosome, and their purpose is to estimate effects in a multilocus model. If the number of markers is more than one million, 50 potentially associated markers are recommended for each chromosome. The setup can balance computational speed and statistical power.

In the second step, significant QTNs are identified by parameter estimation in a multilocus model and by the likelihood ratio test. In parameter estimation, the effects are removed from the multilocus model if their absolute estimates are less than 1.00E−5. In the likelihood ratio test, each marker with a nonzero effect is tested. An LOD score greater than 3.0 is considered to indicate a significant QTN.

Finally, known/candidate genes are mined around these significant QTNs from previous studies and multiomics data analysis.

### Other GWAS methods

The mrMLM (https://cran.microsoft.com/web/packages/mrMLM/) is a multilocus GWAS method [17] in which the QTN effect is treated as random, and the threshold for identifying significant QTNs is set at LOD = 3.0 [18].

FarmCPU (https://zzlab.net/FarmCPU/) is a multilocus GWAS method [32] in which the QTN effect is treated as fixed, and the Bonferroni correction (0.05/*m*, where *m* is the number of markers) is used to determine significant QTNs.

GEMMA (https://github.com/genetics-statistics/GEMMA/releases/) is an exact single-locus GWAS algorithm [8] that treats the QTN effect as fixed and uses Bonferroni correction to determine significant QTNs.

EMMAX (http://csg.sph.umich.edu//kang/emmax/download/index.html) is an existing single-locus and fast GWAS method [6] that treats the QTN effect as fixed and uses Bonferroni correction to determine significant QTNs.

To investigate the effect of ABESS on BLUPmrMLM, ABESS was removed from BLUPmrMLM. This is the control. In the control, the QTN effect is treated as random, and the threshold for significant QTNs is set as LOD = 3.0 [18].

## Results

### Performance comparison of BLUPmrMLM with existing methods

#### Computational efficiency

**Figure 1:**
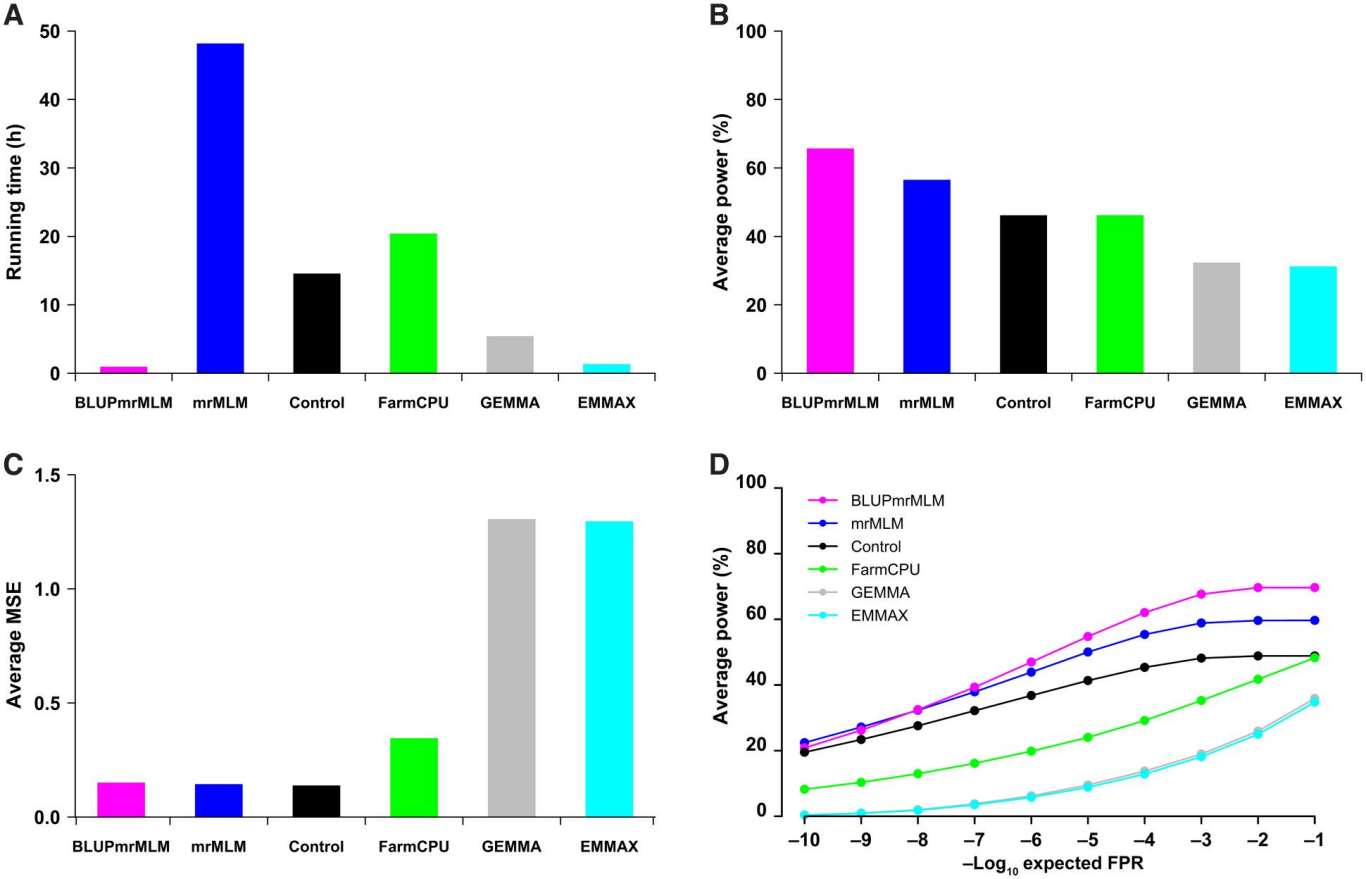
Performance comparison of BLUPmrMLM with existing methods in simulation studies **A**. Running time (h). **B**. Statistical power (%). **C**. Average MSE of the quantitative trait locus effects. **D**. ROC characteristic curve. Control indicates BLUPmrMLM with ABESS removed. MSE, mean squared error; ROC, receiver operating characteristic; FPR, false positive rate.

To verify the speed of the BLUPmrMLM, four Monte Carlo simulation experiments were performed with 300 individuals and 50,000 SNP markers. All the datasets were analyzed by BLUPmrMLM, mrMLM, Control, FarmCPU, GEMMA, and EMMAX. As a result, the average running time (h) for the four Monte Carlo simulation experiments was 1.2897, 48.2689, 14.6661, 20.4987, 5.5024, and 1.4030 for the aforementioned six methods, respectively (**Figure 1**A). This indicates that BLUPmrMLM and EMMAX are the fastest. To further validate this conclusion, five traits in 1439 rice hybrids with 1,098,527 SNPs [41] and two traits in the 3K rice dataset with 2261 accessions and 1,011,601 SNPs [42,43] were reanalyzed by BLUPmrMLM, mrMLM, FarmCPU, GEMMA, and EMMAX. As a result, the running time (h) for the five methods was 0.9167, 38.9576, 3.4341, 4.8139, and 0.2953 for the first five traits, and 0.2644, 17.8622, 0.8982, 1.1764, and 0.1311 for the last two traits, respectively. Clearly, almost the same trend was observed, although BLUPmrMLM was slightly slower than EMMAX.

#### Statistical power for QTN detection

To evaluate the power of BLUPmrMLM, the four simulation datasets were reanalyzed by all the six methods. The average power over all the QTNs in all four experiments was 65.90%, 56.72%, 46.36%, 46.37%, 32.53%, and 31.42% for BLUPmrMLM, mrMLM, Control, FarmCPU, GEMMA, and EMMAX, respectively, indicating that the power of BLUPmrMLM is significantly greater than that of the other methods (*P* = 0.0002–0.0214; Figure 1B, Figures S1 and S2; Table S4), and the average power across all the QTNs identified by BLUPmrMLM was 65.31%, 68.85%, 63.42%, and 66.00% for the four datasets mentioned above (Table S5). When comparing BLUPmrMLM with other methods, the difference in average power between BLUPmrMLM and other methods in the first simulation experiment was more than 20% for small-effect QTNs (*r*^2^ ≤ 5%) and ranged from 1.04% to 11.52% for large-effect QTNs (*r*^2^ > 5%). For two pairs of linked QTNs, the first and second QTNs (positive effects) and the ninth (positive effect) and tenth (negative effect) QTNs, the average power from BLUPmrMLM in the first simulation experiment was more than 40%, while the average power from other methods was less than 20%, especially for GEMMA and EMMAX (≤ 3%). The same trends were also observed in other simulation experiments, although mrMLM had a slightly greater influence on the third QTN than did BLUPmrMLM (Table S5). These results indicate that BLUPmrMLM has high potential for small-effect and linked QTNs, albeit under different polygenic backgrounds. In addition, the strict Bonferroni correction of FarmCPU, GEMMA, and EMMAX results in the missed identification of many important loci.

#### Accuracy of the estimates of QTN effects and positions

To assess the accuracy of the BLUPmrMLM estimates of QTN effects and positions, the four simulation datasets mentioned above were reanalyzed using the aforementioned six methods. In simulation studies I and II, BLUPmrMLM, mrMLM, and the control had the smallest MSEs and MADs for the estimates of QTN effects, followed by FarmCPU, and GEMMA and EMMAX had the largest values (Figure 1C, Figures S3 and S4; Tables S6–S8). The same trends were also observed in simulation studies III and IV, except for the second QTN (Tables S6 and S8). Using paired *t*-test, BLUPmrMLM had significantly lower MSE and MAD for the estimates of QTN effects than did GEMMA or EMMAX (*P* = 0.0001–0.0149) but not for mrMLM, control, or FarmCPU (*P* = 0.1626–0.9912) (Table S4), indicating the high accuracy of BLUPmrMLM, mrMLM, control, and FarmCPU, but not GEMMA or EMMAX.

In all the simulation studies, the MSEs and MADs for the estimates of QTN positions were close to zero for all methods, indicating high accuracy in the estimation of QTN positions (Tables S9 and S10).

#### FPR, FDR, FNR, F_1_ score, and ROC curve

To measure the performance of BLUPmrMLM, the FPR, FNR, and ROC curve were obtained from the aforementioned four simulation studies (Figure 1D, Figures S5 and S6; Table S11). The average FPRs were 0.7792‱, 0.8161‱, 0.9856‱, 0.3544‱, 0.5484‱, and 0.5018‱ for BLUPmrMLM, mrMLM, Control, FarmCPU, GEMMA, and EMMAX, respectively, indicating their relatively low FPRs. Although FarmCPU, GEMMA, and EMMAX had slightly lower FPRs than did BLUPmrMLM, mrMLM, and control due to the strict Bonferroni correction, the former had significantly lower power in QTN detection than did the latter. Moreover, the average FDRs were 37.07%, 41.76%, 51.42%, 27.62%, 45.72%, and 44.36% for the aforementioned six methods, respectively, with BLUPmrMLM having the lowest FDR, except for FarmCPU. Thus, BLUPmrMLM and mrMLM balance high power and low FPR.

The average FNRs for the aforementioned six methods were 34.11%, 43.29%, 53.64%, 53.63%, 67.47%, and 68.58%, respectively, with BLUPmrMLM having the lowest FNR.

The average F_1_ scores for the aforementioned six methods were 0.6436, 0.5745, 0.4741, 0.5650, 0.4067, and 0.4015, respectively, indicating the robustness of BLUPmrMLM compared to the other methods.

In the first simulation study, different significance levels were set from 1.00E−10 to 0.10 to calculate the corresponding statistical power for the ten QTNs. BLUPmrMLM had the largest area under the ROC curve (AUC) for almost all the QTNs except for the fifth QTN (Figure S6). To better evaluate the performance of BLUPmrMLM, the average power across all ten QTNs in each simulation experiment was used to plot their ROC curves. BLUPmrMLM had the largest AUC, and mrMLM had the second largest AUC, further demonstrating that BLUPmrMLM is the best (Figure 1D, Figure S6).

### Identification of QTNs for rice yield-related traits in large-scale datasets

#### Reanalysis of five yield-related traits in 1439 rice hybrids

To validate the performance of BLUPmrMLM in large datasets, HD, GL, Yield, GN, and TGW in 1439 rice hybrids [41] were reanalyzed using BLUPmrMLM, mrMLM, FarmCPU, GEMMA, and EMMAX.

The numbers of significant QTNs, identified by the aforementioned five methods, for the five traits are listed in Table S12, where BLUPmrMLM identified 71, 61, 38, 45, and 76 significant QTNs associated with HD, GL, Yield, GN, and TGW, respectively. To further compare BLUPmrMLM with the other methods, we performed regression of trait phenotypes on all the significant QTNs from each method. BLUPmrMLM had the best model fit for all traits except for GL (the second-best model fit) (Table S13).

**Figure 2:**
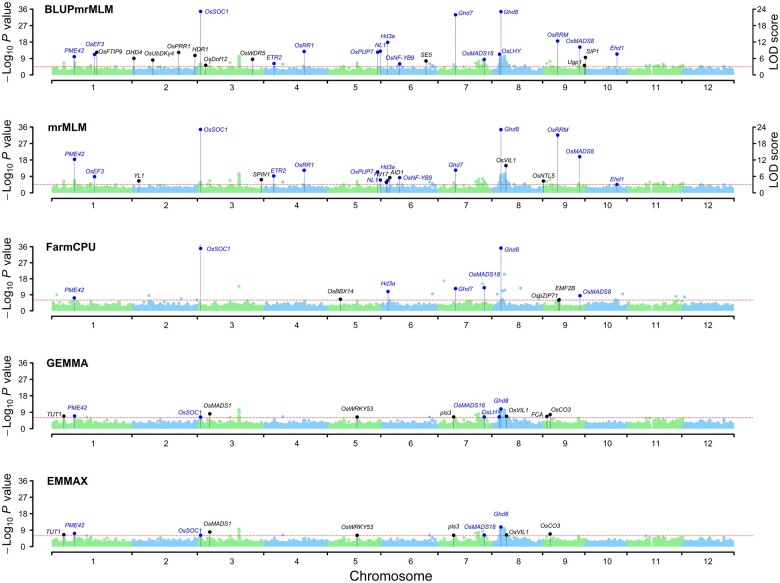
Manhattan plots for HD in Hangzhou in 1439 rice hybrids The known genes commonly detected by BLUPmrMLM and other existing methods are marked in blue, while those detected only by the existing methods are marked in black. The *y*-axis on the left for all the methods shows the –log_10_*P* value obtained from genome-wide single-marker scanning in the first step, while the *y*-axis on the right for mrMLM and BLUPmrMLM shows the LOD scores obtained from the likelihood ratio test for significant (*P* ≤ 0.05/*m*) and suggested (LOD score ≥ 3.0, red dashed line) QTNs in the second step. For LODs ≥ LOD_max_ (the maximum LOD score), the LODs were transformed to $\text{LO}D^{\prime }={\text{LOD}}_{\max}-1+\left(\text{LOD}-{\text{LOD}}_{\max}+1\right)/100$. HD, heading date; LOD, logarithm of odds; QTN, quantitative trait nucleotide.

Around all the significant QTNs mentioned above, BLUPmrMLM, mrMLM, FarmCPU, GEMMA, and EMMAX detected 102, 70, 43, 23, and 20 known genes, respectively, which were further confirmed by haplotype analysis (**Figure 2**, Figures S7–S10; Tables S13 and S14). Of the 102 known genes from BLUPmrMLM, 54 were found by other methods. Almost all of the known genes associated with the aforementioned five traits identified by Huang et al. [41] were also detected by BLUPmrMLM (Figure 2, Figures S7–S10). Clearly, BLUPmrMLM identified more known genes.

#### Reanalysis of GLWR and TGW in the 3K rice dataset

To further validate the performance of BLUPmrMLM in large datasets, GLWR and TGW in the 3K rice dataset [42,43] were reanalyzed using BLUPmrMLM, mrMLM, FarmCPU, GEMMA, and EMMAX.

The numbers of significant QTNs identified by the five methods for GLWR and TGW are listed in Table S15, where BLUPmrMLM identified 48 and 66 significant QTNs for GLWR and TGW, respectively. To further compare BLUPmrMLM with the other methods, we performed regression of trait phenotypes on all the significant QTNs from each method. BLUPmrMLM had the best model fit for TGW and the second-best model fit for GLWR (Table S16).

**Figure 3:**
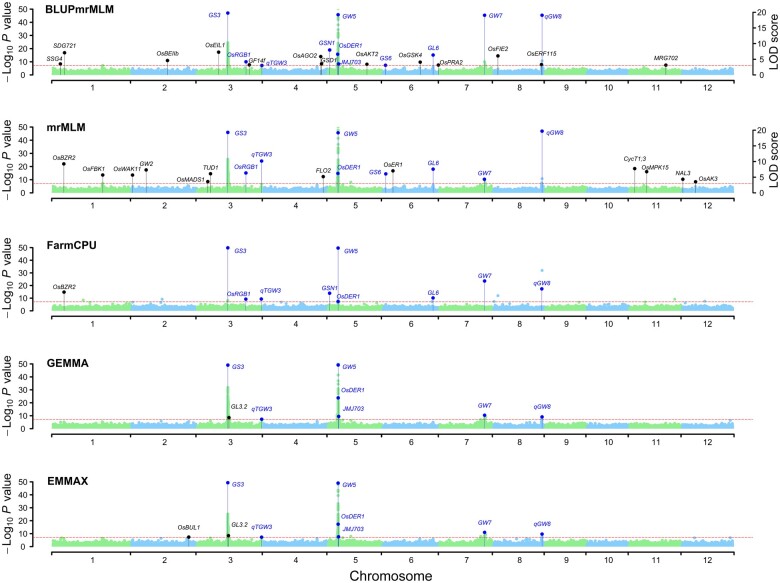
Manhattan plots for GLWR in the 3K rice dataset The known genes commonly detected by BLUPmrMLM and other existing methods are marked in blue, while those detected only by the existing methods are marked in black. The *y*-axis on the left for all the methods shows the –log_10_*P* value obtained from genome-wide single-marker scanning in the first step, while the *y*-axis on the right for mrMLM and BLUPmrMLM shows the LOD scores obtained from the likelihood ratio test for significant (*P* ≤ 0.05/*m*) and suggested (LOD score ≥ 3.0, red dashed line) QTNs in the second step. For LODs ≥ LOD_max_ (the maximum LOD score), the LODs were transformed to ${\mathrm{LOD}}^{\prime }={\mathrm{LOD}}_{\max}-1+(\mathrm{LOD}-{\mathrm{LOD}}_{\max}+1)/100$. GLWR, grain length width ratio.

Around all the QTNs mentioned above, BLUPmrMLM, mrMLM, FarmCPU, GEMMA, and EMMAX detected 53, 42, 18, 15, and 18 known genes for the two traits, respectively, which were further confirmed by haplotype analysis (**Figure 3**, Figure S11; **Table 1**, Table S15). Of the 53 known genes identified by BLUPmrMLM, 25 were also found by other methods (Figure 3, Figure S11). All the results are consistent with those obtained in previous studies (Table 1, Table S17). These results demonstrate the superiority of BLUPmrMLM over other approaches for the detection of known genes.

**Table 1:** Previously reported genes around significant QTNs for GLWR and TGW in the 3K rice dataset using five GWAS methods

Trait	Gene	RAP locus	Marker	Chr	Position	MAF	BLUPmrMLM			mrMLM			FarmCPU	GEMMA	EMMAX	**Distance** **(kb)**	*P* value of haplotype test	ATAC-seq
							LOD	Effect	*r* ^2^ (%)	LOD	Effect	*r* ^2^ (%)	*P* value	*P* value	*P* value			
**GLWR**	*SSG4*	Os01g0179400	rs10422	1	3952184	0.107	3.507	−0.0368	0.1626							175.048	2.74E−31	
	*OsBZR2*	Os01g0203000	rs15314	1	5858653	0.3379				9.2391	0.0477	0.6855				187.186	2.30E−37	✓
			rs15642	1	5962421	0.0672							1.65E−15			290.954		
	*SDG721*	Os01g0218800	rs16695	1	6250695	0.3215	7.0776	0.0401	0.4685							256.661	8.76E−17	✓
	*OsFBK1*	Os01g0659900	rs74667	1	27212743	0.0343				5.6923	0.1024	0.4708				337.712	1.92E−32	✓
	*OsWAK11*	Os02g0111600	rs118047	2	336009	0.2996				5.6776	−0.0426	0.525				291.676	1.17E−20	✓
	*GW2*	Os02g0244100	rs138136	2	7811181	0.4345				7.3201	−0.0804	2.1496				304.042	2.25E−15	✓
	*OsBEIIb*	Os02g0528200	rs163772	2	19527270	0.0874	4.6009	0.0631	0.2233							160.143	4.05E−19	✓
	*OsBUL1*	Os02g0747900	rs202132	2	31189225	0.0181									4.50E−08	234.718	1.79E−39	
	*OsMADS1*	Os03g0215400	rs224234	3	5733410	0.4905				3.5892	−0.0489	0.805				319.492	5.77E−36	✓
	*TUD1*	Os03g0232600	rs227848	3	7230027	0.3368				6.093	0.0411	0.4451				198.416	3.65E−28	✓
	*OsEIL1*	Os03g0324300	rs238434	3	11689579	0.1088	7.2619	0.0824	0.8159							84.651	7.91E−53	
	*GS3*	Os03g0407400	rs250624	3	16733441	0.3602	94.0115	0.1882	10.9481	47.9572	0.2021	12.7197	1.74E−131	7.87E−58	4.84E−50	1.668	1.25E−87	
	*GL3.2*	Os03g0417700	rs251702	3	17057445	0.0234									3.32E−09	282.972	2.58E−30	✓
			rs252368	3	17307455	0.0997								2.57E−09		32.962		
	*OsRGB1*	Os03g0669200	rs276891	3	26617207	0.2912							6.07E−10			210.715	3.54E−24	✓
			rs276968	3	26652912	0.2837				6.3435	0.0537	0.7739				246.42		
			rs277088	3	26707321	0.2888	4.1848	0.0349	0.3316							300.829		
	*GF14f*	Os03g0710800	rs282005	3	28533159	0.4323	3.2154	0.0261	0.2239							130.909	1.86E−46	✓
	*qTGW3*	Os03g0841800	rs296798	3	35111562	0.0668							5.11E−10			274.395	1.99E−25	✓
			rs297266	3	35283486	0.021				10.1569	0.154	0.5962		5.09E−08	5.20E−08	102.471		
			rs297787	3	35435106	0.1559	3.0204	0.0282	0.138							43.124		
	*OsAGO2*	Os04g0615700	rs381670	4	31344502	0.1864	5.8562	−0.0389	0.2949							101.072	1.58E−08	✓
	*GSD1*	Os04g0620200	rs385046	4	31774110	0.3204	3.5407	0.028	0.2264							246.249	2.37E−22	✓
	*FLO2*	Os04g0645100	rs389365	4	32723838	0.2304				5.1822	−0.0485	0.5623				111.442	1.02E−21	✓
	*GSN1*	Os05g0115800	rs396148	5	717134	0.1842							1.33E−14			141.265	4.99E−41	✓
			rs396305	5	761432	0.3965	7.9509	−0.0553	0.9955							96.967		
	*OsDER1*	Os05g0187800	rs408642	5	5149751	0.3072	6.6025	0.0569	0.5942							244.705	8.73E−86	✓
			rs408853	5	5270113	0.1661				6.2007	0.0738	0.9775				124.343		
			rs409071	5	5376245	0.0927							5.15E−08			18.211		
			rs409105	5	5389338	0.3357								1.65E−24	4.82E−18	5.118		
	*GW5*	Os05g0187500	rs409040	5	5361276	0.3715							3.50E−115			3.846	3.16E−38	✓
			rs409060	5	5371949	0.4954	40.688	0.1248	5.1355	36.1816	0.1277	5.518		8.49E−66	2.91E−54	5.248		
	*JMJ703*	Os05g0196500	rs409691	5	5591452	0.0239	3.5073	0.072	0.1732						2.70E−08	348.711	4.31E−02	✓
			rs409813	5	5651540	0.2291								3.67E−10		288.623		
	*OsAKT2*	Os05g0428700	rs450232	5	21192321	0.0714	3.3905	0.0458	0.1831							147.461	4.52E−20	
	*GS6*	Os06g0127800	rs470383	6	1463390	0.1161	3.0849	−0.0306	0.1294							2.11	7.51E−13	✓
			rs471181	6	1625954	0.0462				6.0566	−0.0779	0.3773				157.371		
	*OsER1*	Os06g0203800	rs479867	6	5536745	0.1822				7.0219	0.0456	0.409				284.902	1.49E−28	✓
	*OsGSK4*	Os06g0547900	rs520736	6	20511590	0.0851	4.0826	0.053	0.1393							206.218	1.21E−18	✓
	*GL6*	Os06g0666100	rs539595	6	27564497	0.1073							6.81E−11			2.899	1.06E−25	✓
			rs539698	6	27601168	0.0796	6.3503	0.0756	0.5172	7.5664	0.0697	0.435				39.57		
	*OsPRA2*	Os06g0714600	rs547025	6	30489823	0.2101	3.1508	−0.0281	0.1661							152.817	1.89E−35	✓
	*GW7*	Os07g0603300	rs613171	7	24536015	0.4376				4.332	0.0536	0.9147				128.313	7.15E−34	✓
			rs613372	7	24629753	0.023	23.2464	0.2447	1.8416				2.66E−24	4.23E−11	1.04E−11	34.575		
	*OsFIE2*	Os08g0137100	rs631294	8	2280470	0.069	6.0722	0.0762	0.5181							197.198	2.22E−13	✓
	*OsERF115*	Os08g0521600	rs705616	8	26192238	0.0367	3.3271	0.0659	0.1903							241.081	1.57E−24	✓
	*qGW8*	Os08g0531600	rs706209	8	26380813	0.0126							4.41E−18			120.354	1.10E−36	✓
			rs706632	8	26504638	0.0155	23.5337	0.3286	1.8316	19.6927	0.2753	1.2614		8.17E−10	2.23E−10	1.56		
	*CycT1;3*	Os11g0157100	rs841920	11	2787848	0.0316				7.7448	0.1258	0.5				51.205	1.67E−14	✓
	*OsMPK15*	Os11g0271100	rs861052	11	9377957	0.0847				6.7533	0.0544	0.2849				93.632	9.65E−19	✓
	*MRG702*	Os11g0545600	rs898399	11	19917207	0.0953	3.1177	−0.0342	0.1199							180.859	1.39E−08	✓
	*NAL3*	Os12g0101600	rs937059	12	233600	0.4474				4.3437	−0.0395	0.5187				166.562	9.11E−08	
	*OsAK3*	Os12g0236400	rs951310	12	7218697	0.4774				3.5347	−0.0363	0.433				257.778	1.37E−15	✓
**TGW**	*SSG4*	Os01g0179400	rs10525	1	3985902	0.4646				3.6368	0.4275	0.8666				141.33	1.52E−4	
	*SMG11*	Os01g0197100	rs14361	1	5521069	0.4177	3.2592	−0.2831	0.3765							276.549	1.19E−05	✓
	*SDG721*	Os01g0218800	rs16821	1	6285978	0.0904				10.493	1.1013	1.2587				221.378	2.87E−15	✓
	*YGL8*	Os01g0279100	rs23380	1	9659921	0.0133									3.71E−08	214.458	2.41E−13	✓
	*OsFBK1*	Os01g0659900	rs74390	1	27079447	0.0953	3.3863	−0.4628	0.1681							204.416	3.70E−08	✓
	*OsCCS52B*	Os01g0972900	rs117379	1	43240002	0.2917	4.0455	0.2618	0.2639							287.323	3.70E−08	✓
	*FUWA*	Os02g0234200	rs137622	2	7623771	0.0475	5.1705	0.6709	0.3783							23.903	1.33E−27	✓
			rs137755	2	7692509	0.3173							1.18E−08			92.641		
			rs138092	2	7800124	0.3881				5.7165	0.6131	1.6631				200.256		
	*OsPLIM2a*	Os02g0641000	rs189023	2	25943490	0.1362	5.6016	0.5421	0.6905							202.657	6.76E−05	✓
			rs189668	2	26092847	0.2371									4.38E−08	352.014		
	*OsMRP5*	Os03g0142800	rs217372	3	2569221	0.0391	3.0001	−0.5306	0.2102	3.5575	−0.6306	0.2372	2.23E−08			194.784	4.67E−13	✓
	*BG1*	Os03g0175800	rs219408	3	3756927	0.1194				4.9682	0.8006	0.9715				281.414	2.40E−05	✓
	*OsPUP1*	Os03g0187800	rs220534	3	4368320	0.0487	3.2451	−0.7274	0.3516							230.604	1.36E−04	✓
	*DG1*	Os03g0229500	rs226238	3	6587019	0.1581				8.3582	0.991	2.3121			2.84E−08	266.772	1.40E−14	✓
	*LPA1*	Os03g0237250	rs226979	3	6930244	0.4847				3.3425	0.4079	0.8019				306.918	2.25E−05	✓
	*GS3*	Os03g0407400	rs250497	3	16696473	0.0137							1.17E−18			33.028	3.08E−13	
			rs250624	3	16733441	0.3602	24.0141	0.9777	3.6694	28.1945	1.1614	5.2053		3.66E−11	3.55E−10	1.668		
	*GL3.1*	Os03g0646900	rs272978	3	24807654	0.1059	3.5869	0.5141	0.2374							234.773	1.51E−03	✓
	*RGB1*	Os03g0669200	rs277085	3	26706426	0.3638	4.7309	0.3761	0.6165							299.934	2.06E−13	✓
	*OsINV2*	Os04g0535600	rs372241	4	26852458	0.0902	3.6488	0.5272	0.2342							80.589	1.36E−09	✓
	*GSN1*	Os05g0115800	rs396679	5	831332	0.2291				4.9257	−0.3742	0.4187				27.067	2.00E−08	✓
	*GW5*	Os05g0187500	rs408480	5	5007414	0.3768	8.9059	−0.5827	1.1038							357.708	1.29E−04	✓
			rs409047	5	5363587	0.454				10.3202	0.5857	1.6463		3.60E−08		1.535		
			rs409051	5	5365256	0.3757							3.69E−11			0.134		
	*OsDER1*	Os05g0187800	rs408898	5	5299051	0.1988				7.857	−0.7748	1.5369				95.405	1.22E−13	✓
			rs409051	5	5365256	0.3757							3.69E−11			0.134		
			rs409091	5	5383914	0.2125								1.63E−08		10.542		
			rs409769	5	5617272	0.123	3.0422	−0.3752	0.2379							219.227		
	*OsGSK2*	Os05g0207500	rs412981	5	6818314	0.0982	3.5983	0.4014	0.282							156.821	2.08E−05	✓
	*OsLAC*	Os05g0458600	rs452380	5	22468025	0.207	6.38	0.4848	0.5813							65.511	1.45E−07	
	*OsS40-14*	Os05g0531000	rs459015	5	26365993	0.0252								3.34E−08	6.77E−09	0.398	4.23E−29	✓
	*SSG6*	Os06g0130400	rs470654	6	1510781	0.1592				12.5252	−0.9105	1.969				118.997	1.64E−07	✓
	*DSG1*	Os06g0154500	rs472999	6	2577029	0.1473	4.318	0.4333	0.4671							229.639	1.91E−09	✓
			rs473055	6	2608798	0.1303				4.9554	0.6177	0.8821				197.87		
	*OsUBR7*	Os06g0529800	rs519253	6	19932046	0.1743				4.4211	0.7026	0.6198				262.504	9.08E−13	✓
	*OsGSK4*	Os06g0547900	rs520850	6	20546753	0.4651	3.9321	0.3448	0.4327	5.1944	0.5645	1.3782				171.055	1.60E−09	✓
	*TGW6*	Os06g0623700	rs533452	6	25081743	0.0057							6.41E−08			11.499	6.67E−04	✓
	*OsCYP19-4*	Os06g0708400	rs546149	6	30066598	0.4312				3.4205	0.3763	0.6457				93.625	2.13E−07	✓
			rs546397	6	30171804	0.0312	3.8971	0.6755	0.2867							198.831		
	*RAG2*	Os07g0214300	rs559704	7	6168361	0.228	3.014	0.3272	0.2613							90.556	7.41E−03	✓
	*LC7*	Os07g0658400	rs619861	7	27596731	0.3591	3.9196	0.2819	0.3304							126.358	1.46E−09	✓
	*FZP*	Os07g0669500	rs621236	7	28319838	0.0812	13.7981	−1.1926	2.1479	15.156	−1.3705	2.7552		3.83E−12	2.71E−13	18.749	7.57E−11	✓
			rs621242	7	28323091	0.0831							9.11E−21			22.002		
	*OsEIL2*	Os07g0685700	rs622032	7	28762557	0.0801	6.8419	−0.9577	0.58							353.501	5.56E−04	✓
	*Ghd7.1*	Os07g0695100	rs623102	7	29368338	0.0389	6.254	1.1589	0.7388							248.367	1.85E−05	✓
	*OsCCC1*	Os08g0323700	rs670843	8	14535813	0.1026	3.3126	0.5492	0.4574							340.393	4.41E−09	✓
	*IPA1*	Os08g0509600	rs702329	8	24928701	0.0279								4.95E−08	1.74E−09	345.84	4.10E−07	✓
	*OsSHI1*	Os09g0531600	rs769245	9	20921346	0.0431	3.1289	−0.865	0.5452					2.26E−08	4.83E−09	81.417	5.35E−17	✓
	*YL3*	Os09g0552800	rs770909	9	21757666	0.3569	3.4285	−0.3847	0.5536							145.048	3.93E−05	✓
	*OsSCP46*	Os10g0101200	rs773656	10	134175	0.4314				4.9424	0.4364	0.8483				17.723	1.72E−10	✓
			rs774268	10	398800	0.2105	3.1077	−0.3462	0.3137							282.348		
	*OsMADS56*	Os10g0536100	rs833332	10	21100560	0.0887	7.0199	−0.7163	0.7639	5.4997	−0.7916	0.9192				226.925	5.68E−11	✓
	*OsSMK1*	Os11g0213500	rs851224	11	6255324	0.0261									1.06E−08	358.157	1.03E−07	✓
	*SRS5*	Os11g0247300	rs855595	11	7731678	0.3806							4.77E−09			228.853	4.38E−05	✓
	*SWEET14*	Os11g0508600	rs890142	11	18042330	0.1101	6.7951	0.6876	0.5484	3.8641	0.6425	0.2857				129.377	6.22E−14	✓
	*MRG702*	Os11g0545600	rs899035	11	20049196	0.1061				3.0479	0.472	0.3021				48.87	2.76E−02	✓

*Note*: The known genes were confirmed by haplotype analysis, and the *P* value of the haplotype analysis was obtained from ANOVA for the traits of interest across various haplotypes. The ATAC-seq dataset [50] was derived from http://glab.hzau.edu.cn/RiceENCODE/, in which the genes with open chromosomal regions were marked by “✓”. QTN, quantitative trait nucleotide; GLWR, grain length width ratio; TGW, thousand grain weight; GWAS, genome-wide association study; MAF, minor allelic frequency; LOD, logarithm of odds.

Based on the rice ATAC-seq dataset [50], 210 out of the 233 genes previously reported (Table 1, Table S14) had open chromosomal regions, confirming the reliability of our results.

## Discussion

BLUPmrMLM is a fast method within the methodological framework of our mrMLM method [17]. Compared to the mrMLM, significant progress has been made in terms of computational speed. First, genome-wide single-marker scanning in the mrMLM was replaced by vectorized Wald tests in the BLUPmrMLM. Specifically, a mixed model was used to estimate the genetic variance only once. Within the framework of BLUP, the effect vector (γ) of all the markers in the genome is predicted, and their variances are simplified as a vector. This allows Wald tests to be performed on all effects in a vectorial fashion, saving considerable computational time. The ABESS from Zhu et al. [40] was subsequently used to determine potentially associated markers from the reduced SNP markers. This reduces the running time of empirical Bayes estimation [49]. Finally, parallel computing and shared memory schemes were integrated into our software to increase computational speed and reduce memory usage. According to our simulation results, BLUPmrMLM is faster than EMMAX, strongly validating the ability of BLUPmrMLM to reduce running time. Note that BLUPmrMLM is slightly slower than EMMAX in real data analysis, because large population sizes increase running time in empirical Bayes. Therefore, BLUPmrMLM is valuable for analyzing large datasets, such as those from phenomics, transcriptomics, metabolomics, and proteomics.

Although BLUPmrMLM is a fast mrMLM algorithm, it outperforms mrMLM in our simulation studies and real data analyses. The possible reasons for this are as follows. First, the ABESS in BLUPmrMLM may be better than the decollinearity treatment in mrMLM for obtaining potential association markers, because the markers with the minimum probabilities in genome-wide single-marker scanning may not be identified as significant QTNs in the multilocus model in real data analysis. This finding suggests that the ABESS algorithm may minimize the loss of significant QTNs. Second, residual errors after multilocus model fitting are used for further association with all the unassociated markers to identify additional QTNs in BLUPmrMLM [30].

In addition, BLUPmrMLM had lower FPRs than did mrMLM, while BLUPmrMLM and mrMLM had relatively low MSE and MAD for the estimates of QTN parameters (Figure 1C, Figures S3−S5; Tables S4−S11). Because most of the SNPs not associated with traits were removed by the vectorized Wald tests and ABESS algorithm, all the remaining SNPs were closer to the true QTNs, and empirical Bayes can be used to effectively identify true QTNs and shrink the effects of false QTNs to zero. When ABESS is removed from BLUPmrMLM, the control has significantly less power than does BLUPmrMLM. According to our simulation studies, almost all the MSEs and MADs for the estimates of QTN positions are close to zero (Tables S9 and S10), while all the average estimates of QTN effects are close to their true values (Tables S6−S8). Thus, BLUPmrMLM has high accuracy and low FPRs. This finding is consistent with those of our previous studies. This finding suggests that, compared with several penalized methods, such as SCAD, the BLUPmrMLM is a more efficient way to handle large datasets in GWAS [51].

Although multilocus GWAS methods have many advantages [14,17,18,22–24,29–31,52], almost all the GWAS methods currently available for large datasets are single-locus methods, because there are numerous challenges associated with high-dimensional genotype data in multilocus GWAS methods [53]. Although several penalized- and Bayes-based multilocus methods have been used to address this problem [23,25,54,55], these methods fail when the number of markers is many times larger than the population size.

BLUPmrMLM differs from existing multilocus methods. First, BLUPmrMLM differs from our previous multilocus methods, such as mrMLM, as described above, although these methods have the same steps. Then, BLUPmrMLM differs from the MLMM of Segura et al. [24], in which all the potentially associated markers are first selected on the basis of their size to enter the multilocus model one by one. Then, each of the least significant markers is gradually eliminated from the model, and finally, its optimal genetic model is used to determine significant QTNs. The stepwise variable selection method may limit the exploration of a large model space, resulting in some important loci being missed, especially with Bonferroni correction. Finally, BLUPmrMLM differs from FarmCPU [32], which iteratively uses a fixed-effect model and a random-effect model. In statistics, FarmCPU looks like stepwise regression under the framework of MLM.

Although the vectorized Wald tests for all the effects in BLUPmrMLM are similar to those in the studies by Gualdrón Duarte et al. [36], Ning et al. [37], and Wang et al. [38], BLUPmrMLM differs from them in several ways. First, BLUPmrMLM is a multistep method, whereas the other methods are single-step methods. For the first time, Gualdrón Duarte et al. [36] proposed a standardized test of marker effects using variance to detect specific genomic regions. This method was further extended by Ning et al. [37] in a nonpolygenic background and by Wang et al. [38] in a normal distribution polygenic background to identify epistatic effects. Recently, Wang et al. [39] proposed deshrinking ridge regression to deshrink the estimated effect and its standard error so that the Wald test is returned to the same level as that of EMMA. All of these methods detect all of the markers simultaneously. The aforementioned idea is adopted in this study. However, its purpose is to select potentially associated markers rather than to identify significant QTNs or epistasis. Second, BLUPmrMLM uses the AI algorithm to estimate genetic variance components, which is different from the EM and average information algorithm in the study by Wang et al. [38] and the L-BFGS-B algorithm in the study by Wang et al. [39]. The EM algorithm is used in BLUPmrMLM to determine the initial values of the AI algorithm to optimize the restricted likelihood function. The EM algorithm is also used when the change in parameters between iterations of the AI algorithm is small.

## Conclusion

For the selection of potentially associated markers, genome scanning in mrMLM was replaced by vectorized Wald tests and ABESS in the BLUPmrMLM. A shared memory parallel computing scheme was implemented to improve the computational performance. According to a series of simulated and real data analyses, BLUPmrMLM significantly saved computational time, improved statistical power and accuracy, and had a low FPR. More importantly, in rice real data analyses, BLUPmrMLM detected more known genes than did mrMLM, FarmCPU, GEMMA, and EMMAX. BLUPmrMLM will be available for analysis of large datasets.

## Code availability

The software mrMLM v5.1 used is available at BioCode (https://ngdc.cncb.ac.cn/biocode/tool/BT007388) or GitHub (https://github.com/YuanmingZhang65/mrMLM).

## CRediT author statement

**Hong-Fu Li:** Conceptualization, Methodology, Software, Investigation, Validation, Formal analysis, Visualization, Writing – original draft. **Jing-Tian Wang:** Software, Investigation, Validation, Formal analysis, Visualization, Writing – review & editing. **Qiong Zhao:** Investigation, Validation. **Yuan-Ming Zhang:** Conceptualization, Methodology, Supervision, Writing – review & editing, Resources. All authors have read and approved the final manuscript.

## Supplementary material

Supplementary material is available at *Genomics, Proteomics & Bioinformatics* online (https://doi.org/10.1093/gpbjnl/qzae020).

## Competing interests

The authors have declared no competing interests.

## Supplementary Material

qzae020_Supplementary_Data
