# Supplementary material for: BLUPmrMLM: A Fast mrMLM Algorithm in Genome-wide Association Studies
Source: Genomics Proteomics Bioinformatics. 2024 Feb 29;22(3):qzae020. doi: 10.1093/gpbjnl/qzae020 (PMC12016565; doi:10.1093/gpbjnl/qzae020)
Supplement: qzae020_Supplementary_Data [file qzae020_supplementary_data.zip › Table S12.docx]

**Table S12** **The numbers of QTNs for the five traits in 1439 rice hybrids and their previously reported genes detected by the new and existing methods**

| **Trait** | **BLUPmrMLM** | | **mrMLM** | | **FarmCPU** | | **GEMMA** | | **EMMAX** | |
| --- | --- | --- | --- | --- | --- | --- | --- | --- | --- | --- |
|  | **Genes** | **QTNs** | **Genes** | **QTNs** | **Genes** | **QTNs** | **Genes** | **QTNs** | **Genes** | **QTNs** |
| HD | 26 | 71 | 20 | 67 | 10 | 33 | 12 | 806 | 10 | 140 |
| GL | 23 | 61 | 17 | 60 | 12 | 56 | 3 | 1636 | 3 | 1546 |
| Yield | 10 | 38 | 8 | 26 | 3 | 8 | 3 | 8 | 2 | 6 |
| GN | 15 | 45 | 10 | 44 | 7 | 27 | 1 | 412 | 1 | 410 |
| TGW | 28 | 76 | 15 | 67 | 11 | 45 | 4 | 781 | 4 | 748 |

*Note*: Genes, the number of previously reported genes; QTNs, the number of significantly associated QTNs; HD, heading date in Hangzhou; GL, grain length in Hangzhou; Yield, yield per plant in Sanya; GN, grain number in Sanya; TGW, grain weight in Sanya.
