# Supplementary material for: BLUPmrMLM: A Fast mrMLM Algorithm in Genome-wide Association Studies
Source: Genomics Proteomics Bioinformatics. 2024 Feb 29;22(3):qzae020. doi: 10.1093/gpbjnl/qzae020 (PMC12016565; doi:10.1093/gpbjnl/qzae020)
Supplement: qzae020_Supplementary_Data [file qzae020_supplementary_data.zip › Table S11.docx]

**Table S11 FPRs (‱), FDRs (%), FNRs (%), and F1 scores using different methods in four simulation experiments**

| **Method** | **Experiment Ⅰ** | | | |  | **Experiment Ⅱ** | | | |  | **Experiment Ⅲ** | | | |  | **Experiment Ⅳ** | | | |
| --- | --- | --- | --- | --- | --- | --- | --- | --- | --- | --- | --- | --- | --- | --- | --- | --- | --- | --- | --- |
|  | **FPR** | **FDR** | **FNR** | **F1 score** |  | **FPR** | **FDR** | **FNR** | **F1 score** |  | **FPR** | **FDR** | **FNR** | **F1 score** |  | **FPR** | **FDR** | **FNR** | **F1 score** |
| BLUPmrMLM | 0.6815 | 34.28 | 34.69 | 0.6551 |  | 0.7093 | 33.99 | 31.15 | 0.674 |  | 0.8434 | 39.93 | 36.58 | 0.6170 |  | 0.8826 | 40.07 | 34.00 | 0.6282 |
| mrMLM | 0.6927 | 37.77 | 42.95 | 0.5953 |  | 0.7437 | 37.47 | 37.96 | 0.6228 |  | 0.8936 | 46.13 | 47.83 | 0.5301 |  | 0.9342 | 45.65 | 44.40 | 0.5497 |
| Control | 0.8604 | 48.39 | 54.12 | 0.4858 |  | 0.9140 | 47.07 | 48.63 | 0.5214 |  | 1.0298 | 54.82 | 57.58 | 0.4375 |  | 1.138 | 55.41 | 54.22 | 0.4518 |
| FarmCPU | 0.3070 | 25.26 | 54.59 | 0.565 |  | 0.3579 | 26.47 | 50.29 | 0.5932 |  | 0.3587 | 29.06 | 56.23 | 0.5414 |  | 0.3941 | 29.71 | 53.40 | 0.5604 |
| GEMMA | 0.4960 | 43.47 | 67.75 | 0.4107 |  | 0.5513 | 44.51 | 65.64 | 0.4244 |  | 0.5665 | 47.63 | 68.86 | 0.3906 |  | 0.5799 | 47.25 | 67.63 | 0.4012 |
| EMMAX | 0.4460 | 41.91 | 69.10 | 0.4034 |  | 0.5079 | 43.26 | 66.70 | 0.4197 |  | 0.5199 | 46.30 | 69.86 | 0.3861 |  | 0.5333 | 45.97 | 68.66 | 0.3967 |

*Note*: FPRs, false positive rates; FDR, false discovery rate; FNR, false negative rate.
