# Supplementary material for: BLUPmrMLM: A Fast mrMLM Algorithm in Genome-wide Association Studies
Source: Genomics Proteomics Bioinformatics. 2024 Feb 29;22(3):qzae020. doi: 10.1093/gpbjnl/qzae020 (PMC12016565; doi:10.1093/gpbjnl/qzae020)
Supplement: qzae020_Supplementary_Data [file qzae020_supplementary_data.zip › Table S4.docx]

**Table S4** **Paired *t*-tests of powers and MSE and MAD of QTN effects between the new and existing methods**

| **Indicator** | **Experiment** | **mrMLM** | | **Control** | | **GEMMA** | | **FarmCPU** | | **EMMAX** | |
| --- | --- | --- | --- | --- | --- | --- | --- | --- | --- | --- | --- |
|  |  | ***T* value** | ***P* value** | ***T* value** | ***P* value** | ***T* value** | ***P* value** | ***T* value** | ***P* value** | ***T* value** | ***P* value** |
| Power (%) | **Ⅰ** | 3.4532 | 0.0072^***^ | 5.8153 | 0.0003^***^ | 4.5534 | 0.0014^***^ | 5.4179 | 0.0004^***^ | 4.8051 | 0.0010^***^ |
|  | **Ⅱ** | 2.9092 | 0.0173^**^ | 6.096 | 0.0002^***^ | 4.2381 | 0.0022^***^ | 4.4238 | 0.0017^***^ | 4.4038 | 0.0017^***^ |
|  | **Ⅲ** | 3.0928 | 0.0129^**^ | 3.8696 | 0.0038^***^ | 4.6537 | 0.0012^***^ | 6.1911 | 0.0002^***^ | 4.8065 | 0.0010^***^ |
|  | **Ⅳ** | 2.7787 | 0.0214^**^ | 3.6883 | 0.005^***^ | 4.4204 | 0.0017^***^ | 5.6513 | 0.0003^***^ | 4.5409 | 0.0014^***^ |
| MSE | **Ⅰ** | –1.1053 | 0.2977 | –1.1648 | 0.2740 | –3.3311 | 0.0088^***^ | –1.0464 | 0.3227 | –3.2502 | 0.0100^***^ |
|  | **Ⅱ** | –0.1691 | 0.8694 | 0.014 | 0.9891 | –4.336 | 0.0019^***^ | –1.0165 | 0.3360 | –4.3268 | 0.0019^***^ |
|  | **Ⅲ** | 0.4398 | 0.6705 | 0.757 | 0.4684 | –3.004 | 0.0149^**^ | –0.9783 | 0.3535 | –3.2276 | 0.0104^**^ |
|  | **Ⅳ** | 0.7086 | 0.4965 | 0.8038 | 0.4422 | –3.99 | 0.0032^***^ | –0.9387 | 0.3724 | –4.0374 | 0.0029^***^ |
| MAD | **Ⅰ** | –0.9945 | 0.3459 | –1.5208 | 0.1626 | –6.102 | 0.0002^***^ | –0.9654 | 0.3596 | –5.9831 | 0.0002^***^ |
|  | **Ⅱ** | –0.2519 | 0.8068 | –0.5298 | 0.6091 | –6.7374 | 0.0001^***^ | –0.9018 | 0.3907 | –6.7075 | 0.0001^***^ |
|  | **Ⅲ** | –0.0114 | 0.9912 | 0.3875 | 0.7074 | –4.2084 | 0.0023^***^ | –0.7708 | 0.4606 | –4.3017 | 0.0020^***^ |
|  | **Ⅳ** | 0.4282 | 0.6786 | 0.673 | 0.5179 | –6.6147 | 0.0001^***^ | –0.6789 | 0.5143 | –6.7027 | 0.0001^***^ |

*Note*: H_0_: there are no significance differences of indicators between BLUPmrMLM and other GWAS methods. *, **, and ***, significances at the 0.1, 0.05, and 0.01 probability levels, respectively. MAD, mean absolute deviation; MSE, mean squared error; QTN: quantitative trait nucleotide.
