# Supplementary material for: BLUPmrMLM: A Fast mrMLM Algorithm in Genome-wide Association Studies
Source: Genomics Proteomics Bioinformatics. 2024 Feb 29;22(3):qzae020. doi: 10.1093/gpbjnl/qzae020 (PMC12016565; doi:10.1093/gpbjnl/qzae020)
Supplement: qzae020_Supplementary_Data [file qzae020_supplementary_data.zip › Supplementary material captions.docx]

## Supplementary material

**Figure S1** **Statistical power of all the QTNs in four simulation experiments with different polygenic backgrounds using the new and existing methods**

**A.** no polygenic background; **B.** additive polygenic background; **C.** epistatic polygenic background; **D.** additive plus epistatic polygenic backgrounds; new: BLUPmrMLM; Control: the control method of BLUPmrMLM; existing: mrMLM, GEMMA, FarmCPU, and EMMAX. The descriptions above are the same as those in Figures S2 to S6.

**Figure S2** **Average power across all the QTNs in four simulation experiments with different polygenic backgrounds using the new and existing methods**

**Figure S3** **MSPE of all the QTN effects in four simulation experiments with different polygenic backgrounds using the new and existing methods**

**Figure S4** **Mean absolute deviations of all the QTN effects in four simulation experiments with different polygenic backgrounds using the new and existing methods**

**Figure S5** **False positive rates (‱) in four simulation experiments with different polygenic backgrounds using the new and existing methods**

**Figure S6** **Receiver operating characteristic curve** **for each QTN and their average in four simulation experiments using the new and existing methods**

**Figure S7** **Manhattan plot for grain length at Hangzhou in 1439 rice hybrids.**

**Figure S8 Manhattan plot for yield per plant at Sanya in 1439 rice hybrids**

**Figure S9 Manhattan plot for grain number at Sanya in 1439 rice hybrids**

**Figure S10 Manhattan plot for grain weight at Sanya in 1439 rice hybrids**

**Figure S11 Manhattan plot for thousand grain weight in the 3K rice dataset**

**Table S1 Genotypic datasets used in the simulation experiments and derived from Zhu et al. [44]**

**Table S2 Positions and effects of simulated QTNs in Monte Carlo simulation experiments**

**Table S3 Five pairs of epistatic QTNs in simulation experiment Ⅳ**

**Table S4 Paired *t*-tests of power and MSE and MAD of QTN effects between the new and existing methods**

**Table S5** **Statistical power of QTN detection in four simulation experiments using different methods**

**Table S6** **Mean square errors of the QTN effects in four simulation experiments using different methods**

**Table S7** **Mean absolute deviations of the QTN effects in four simulation experiments using different methods**

**Table S8** **Average estimates for QTN effects in four simulation experiments using different methods**

**Table S9 MSEs of QTN positions in four simulation experiments using different methods**

**Table S10 Mean absolute deviations of QTN positions in four simulation experiments using different methods**

**Table S11 False positive rate (‱), false discovery rate (%), false negative rate (%), and F1 score using different methods in four simulation experiments**

**Table S12 The numbers of QTNs for the five traits in 1439 rice hybrids and their previously reported genes detected by the new and existing methods**

**Table S13 Model fitting results for the regression of trait phenotypes on all QTNs from the new and existing methods in 1439 rice hybrids**

**Table S14 Previously reported genes around significant QTNs for the five traits in 1439 rice hybrids using new and existing methods**

**Table S15 The numbers of QTNs and their previously reported genes for the two traits in the 3K rice dataset detected by the new and existing methods**

**Table S16 Model fitting results for the regression of trait phenotypes on all QTNs from the new and existing methods in the 3K rice dataset**

**Table S17 Known genes around QTNs for grain length width ratio and thousand grain weight in 3K rice dataset**
