# Supplementary material for: BLUPmrMLM: A Fast mrMLM Algorithm in Genome-wide Association Studies
Source: Genomics Proteomics Bioinformatics. 2024 Feb 29;22(3):qzae020. doi: 10.1093/gpbjnl/qzae020 (PMC12016565; doi:10.1093/gpbjnl/qzae020)
Supplement: qzae020_Supplementary_Data [file qzae020_supplementary_data.zip › Table S17.docx]

**Table S17**  **Previously reported genes around significant QTNs for GLWR and TGW in 3K rice dataset using five GWAS methods**

| **Trait** | **Gene** | **RAP locus** | **Marker** | **Chr** | **Position** | **MAF** | **BLUPmrMLM** | | | **mrMLM** | | | **FarmCPU** | **GEMMA** | **EMMAX** | **Distance**  **(kb)** | ***P* value of haplotype test** | **Ref.** | **ATAC-seq** |
| --- | --- | --- | --- | --- | --- | --- | --- | --- | --- | --- | --- | --- | --- | --- | --- | --- | --- | --- | --- |
|  |  |  |  |  |  |  | **LOD** | **Effect** | **R^2^ (%)** | **LOD** | **Effect** | **R^2^ (%)** | ***P* value** | ***P* value** | ***P* value** |  |  |  |  |
| **GLWR** | *SSG4* | Os01g0179400 | rs10422 | 1 | 3952184 | 0.107 | 3.507 | -0.0368 | 0.1626 |  |  |  |  |  |  | 175.048 | 2.74E−31 | [1] |  |
|  | *OsBZR2* | Os01g0203000 | rs15314 | 1 | 5858653 | 0.3379 |  |  |  | 9.2391 | 0.0477 | 0.6855 |  |  |  | 187.186 | 2.30E−37 | [2] | ✓ |
|  |  |  | rs15642 | 1 | 5962421 | 0.0672 |  |  |  |  |  |  | 1.65E−15 |  |  | 290.954 |  |  |  |
|  | *SDG721* | Os01g0218800 | rs16695 | 1 | 6250695 | 0.3215 | 7.0776 | 0.0401 | 0.4685 |  |  |  |  |  |  | 256.661 | 8.76E−17 | [3] | ✓ |
|  | *OsFBK1* | Os01g0659900 | rs74667 | 1 | 27212743 | 0.0343 |  |  |  | 5.6923 | 0.1024 | 0.4708 |  |  |  | 337.712 | 1.92E−32 | [4] | ✓ |
|  | *OsWAK11* | Os02g0111600 | rs118047 | 2 | 336009 | 0.2996 |  |  |  | 5.6776 | -0.0426 | 0.525 |  |  |  | 291.676 | 1.17E−20 | [5] | ✓ |
|  | *GW2* | Os02g0244100 | rs138136 | 2 | 7811181 | 0.4345 |  |  |  | 7.3201 | -0.0804 | 2.1496 |  |  |  | 304.042 | 2.25E−15 | [6] | ✓ |
|  | *OsBEIIb* | Os02g0528200 | rs163772 | 2 | 19527270 | 0.0874 | 4.6009 | 0.0631 | 0.2233 |  |  |  |  |  |  | 160.143 | 4.05E−19 | [7] | ✓ |
|  | *OsBUL1* | Os02g0747900 | rs202132 | 2 | 31189225 | 0.0181 |  |  |  |  |  |  |  |  | 4.50E−08 | 234.718 | 1.79E−39 | [8] |  |
|  | *OsMADS1* | Os03g0215400 | rs224234 | 3 | 5733410 | 0.4905 |  |  |  | 3.5892 | -0.0489 | 0.805 |  |  |  | 319.492 | 5.77E−36 | [9] | ✓ |
|  | *TUD1* | Os03g0232600 | rs227848 | 3 | 7230027 | 0.3368 |  |  |  | 6.093 | 0.0411 | 0.4451 |  |  |  | 198.416 | 3.65E−28 | [10] | ✓ |
|  | *OsEIL1* | Os03g0324300 | rs238434 | 3 | 11689579 | 0.1088 | 7.2619 | 0.0824 | 0.8159 |  |  |  |  |  |  | 84.651 | 7.91E−53 | [11] |  |
|  | *GS3* | Os03g0407400 | rs250624 | 3 | 16733441 | 0.3602 | 94.0115 | 0.1882 | 10.9481 | 47.9572 | 0.2021 | 12.7197 | 1.74E−131 | 7.87E−58 | 4.84E−50 | 1.668 | 1.25E−87 | [12] |  |
|  | *GL3.2* | Os03g0417700 | rs251702 | 3 | 17057445 | 0.0234 |  |  |  |  |  |  |  |  | 3.32E−09 | 282.972 | 2.58E−30 | [13] | ✓ |
|  |  |  | rs252368 | 3 | 17307455 | 0.0997 |  |  |  |  |  |  |  | 2.57E−09 |  | 32.962 |  |  |  |
|  | *OsRGB1* | Os03g0669200 | rs276891 | 3 | 26617207 | 0.2912 |  |  |  |  |  |  | 6.07E−10 |  |  | 210.715 | 3.54E−24 | [14] | ✓ |
|  |  |  | rs276968 | 3 | 26652912 | 0.2837 |  |  |  | 6.3435 | 0.0537 | 0.7739 |  |  |  | 246.42 |  |  |  |
|  |  |  | rs277088 | 3 | 26707321 | 0.2888 | 4.1848 | 0.0349 | 0.3316 |  |  |  |  |  |  | 300.829 |  |  |  |
|  | *GF14f* | Os03g0710800 | rs282005 | 3 | 28533159 | 0.4323 | 3.2154 | 0.0261 | 0.2239 |  |  |  |  |  |  | 130.909 | 1.86E−46 | [15] | ✓ |
|  | *qTGW3* | Os03g0841800 | rs296798 | 3 | 35111562 | 0.0668 |  |  |  |  |  |  | 5.11E−10 |  |  | 274.395 | 1.99E−25 | [16] | ✓ |
|  |  |  | rs297266 | 3 | 35283486 | 0.021 |  |  |  | 10.1569 | 0.154 | 0.5962 |  | 5.09E−08 | 5.20E−08 | 102.471 |  |  |  |
|  |  |  | rs297787 | 3 | 35435106 | 0.1559 | 3.0204 | 0.0282 | 0.138 |  |  |  |  |  |  | 43.124 |  |  |  |
|  | *OsAGO2* | Os04g0615700 | rs381670 | 4 | 31344502 | 0.1864 | 5.8562 | -0.0389 | 0.2949 |  |  |  |  |  |  | 101.072 | 1.58E−08 | [17] | ✓ |
|  | *GSD1* | Os04g0620200 | rs385046 | 4 | 31774110 | 0.3204 | 3.5407 | 0.028 | 0.2264 |  |  |  |  |  |  | 246.249 | 2.37E−22 | [18] | ✓ |
|  | *FLO2* | Os04g0645100 | rs389365 | 4 | 32723838 | 0.2304 |  |  |  | 5.1822 | -0.0485 | 0.5623 |  |  |  | 111.442 | 1.02E−21 | [19] | ✓ |
|  | *GSN1* | Os05g0115800 | rs396148 | 5 | 717134 | 0.1842 |  |  |  |  |  |  | 1.33E−14 |  |  | 141.265 | 4.99E−41 | [20] | ✓ |
|  |  |  | rs396305 | 5 | 761432 | 0.3965 | 7.9509 | -0.0553 | 0.9955 |  |  |  |  |  |  | 96.967 |  |  |  |
|  | *OsDER1* | Os05g0187800 | rs408642 | 5 | 5149751 | 0.3072 | 6.6025 | 0.0569 | 0.5942 |  |  |  |  |  |  | 244.705 | 8.73E−86 | [21] | ✓ |
|  |  |  | rs408853 | 5 | 5270113 | 0.1661 |  |  |  | 6.2007 | 0.0738 | 0.9775 |  |  |  | 124.343 |  |  |  |
|  |  |  | rs409071 | 5 | 5376245 | 0.0927 |  |  |  |  |  |  | 5.15E−08 |  |  | 18.211 |  |  |  |
|  |  |  | rs409105 | 5 | 5389338 | 0.3357 |  |  |  |  |  |  |  | 1.65E−24 | 4.82E−18 | 5.118 |  |  |  |
|  | *GW5* | Os05g0187500 | rs409040 | 5 | 5361276 | 0.3715 |  |  |  |  |  |  | 3.50E−115 |  |  | 3.846 | 3.16E−38 | [22] | ✓ |
|  |  |  | rs409060 | 5 | 5371949 | 0.4954 | 40.688 | 0.1248 | 5.1355 | 36.1816 | 0.1277 | 5.518 |  | 8.49E−66 | 2.91E−54 | 5.248 |  |  |  |
|  | *JMJ703* | Os05g0196500 | rs409691 | 5 | 5591452 | 0.0239 | 3.5073 | 0.072 | 0.1732 |  |  |  |  |  | 2.70E−08 | 348.711 | 4.31E−02 | [23] | ✓ |
|  |  |  | rs409813 | 5 | 5651540 | 0.2291 |  |  |  |  |  |  |  | 3.67E−10 |  | 288.623 |  |  |  |
|  | *OsAKT2* | Os05g0428700 | rs450232 | 5 | 21192321 | 0.0714 | 3.3905 | 0.0458 | 0.1831 |  |  |  |  |  |  | 147.461 | 4.52E−20 | [24] |  |
|  | *GS6* | Os06g0127800 | rs470383 | 6 | 1463390 | 0.1161 | 3.0849 | -0.0306 | 0.1294 |  |  |  |  |  |  | 2.11 | 7.51E−13 | [25] | ✓ |
|  |  |  | rs471181 | 6 | 1625954 | 0.0462 |  |  |  | 6.0566 | -0.0779 | 0.3773 |  |  |  | 157.371 |  |  |  |
|  | *OsER1* | Os06g0203800 | rs479867 | 6 | 5536745 | 0.1822 |  |  |  | 7.0219 | 0.0456 | 0.409 |  |  |  | 284.902 | 1.49E−28 | [26] | ✓ |
|  | *OsGSK4* | Os06g0547900 | rs520736 | 6 | 20511590 | 0.0851 | 4.0826 | 0.053 | 0.1393 |  |  |  |  |  |  | 206.218 | 1.21E−18 | [2] | ✓ |
|  | *GL6* | Os06g0666100 | rs539595 | 6 | 27564497 | 0.1073 |  |  |  |  |  |  | 6.81E−11 |  |  | 2.899 | 1.06E−25 | [27] | ✓ |
|  |  |  | rs539698 | 6 | 27601168 | 0.0796 | 6.3503 | 0.0756 | 0.5172 | 7.5664 | 0.0697 | 0.435 |  |  |  | 39.57 |  |  |  |
|  | *OsPRA2* | Os06g0714600 | rs547025 | 6 | 30489823 | 0.2101 | 3.1508 | -0.0281 | 0.1661 |  |  |  |  |  |  | 152.817 | 1.89E−35 | [28] | ✓ |
|  | *GW7* | Os07g0603300 | rs613171 | 7 | 24536015 | 0.4376 |  |  |  | 4.332 | 0.0536 | 0.9147 |  |  |  | 128.313 | 7.15E−34 | [29] | ✓ |
|  |  |  | rs613372 | 7 | 24629753 | 0.023 | 23.2464 | 0.2447 | 1.8416 |  |  |  | 2.66E−24 | 4.23E−11 | 1.04E−11 | 34.575 |  |  |  |
|  | *OsFIE2* | Os08g0137100 | rs631294 | 8 | 2280470 | 0.069 | 6.0722 | 0.0762 | 0.5181 |  |  |  |  |  |  | 197.198 | 2.22E−13 | [30] | ✓ |
|  | *OsERF115* | Os08g0521600 | rs705616 | 8 | 26192238 | 0.0367 | 3.3271 | 0.0659 | 0.1903 |  |  |  |  |  |  | 241.081 | 1.57E−24 | [11] | ✓ |
|  | *qGW8* | Os08g0531600 | rs706209 | 8 | 26380813 | 0.0126 |  |  |  |  |  |  | 4.41E−18 |  |  | 120.354 | 1.10E−36 | [29] | ✓ |
|  |  |  | rs706632 | 8 | 26504638 | 0.0155 | 23.5337 | 0.3286 | 1.8316 | 19.6927 | 0.2753 | 1.2614 |  | 8.17E−10 | 2.23E−10 | 1.56 |  |  |  |
|  | *CycT1;3* | Os11g0157100 | rs841920 | 11 | 2787848 | 0.0316 |  |  |  | 7.7448 | 0.1258 | 0.5 |  |  |  | 51.205 | 1.67E−14 | [31] | ✓ |
|  | *OsMPK15* | Os11g0271100 | rs861052 | 11 | 9377957 | 0.0847 |  |  |  | 6.7533 | 0.0544 | 0.2849 |  |  |  | 93.632 | 9.65E−19 | [32] | ✓ |
|  | *MRG702* | Os11g0545600 | rs898399 | 11 | 19917207 | 0.0953 | 3.1177 | -0.0342 | 0.1199 |  |  |  |  |  |  | 180.859 | 1.39E−08 | [33] | ✓ |
|  | *NAL3* | Os12g0101600 | rs937059 | 12 | 233600 | 0.4474 |  |  |  | 4.3437 | -0.0395 | 0.5187 |  |  |  | 166.562 | 9.11E−08 | [34] |  |
|  | *OsAK3* | Os12g0236400 | rs951310 | 12 | 7218697 | 0.4774 |  |  |  | 3.5347 | -0.0363 | 0.433 |  |  |  | 257.778 | 1.37E−15 | [35] | ✓ |
| **TGW** | *SSG4* | Os01g0179400 | rs10525 | 1 | 3985902 | 0.4646 |  |  |  | 3.6368 | 0.4275 | 0.8666 |  |  |  | 141.33 | 1.52E−4 | [1] |  |
|  | *SMG11* | Os01g0197100 | rs14361 | 1 | 5521069 | 0.4177 | 3.2592 | -0.2831 | 0.3765 |  |  |  |  |  |  | 276.549 | 1.19E−05 | [36] | ✓ |
|  | *SDG721* | Os01g0218800 | rs16821 | 1 | 6285978 | 0.0904 |  |  |  | 10.493 | 1.1013 | 1.2587 |  |  |  | 221.378 | 2.87E−15 | [3] | ✓ |
|  | *YGL8* | Os01g0279100 | rs23380 | 1 | 9659921 | 0.0133 |  |  |  |  |  |  |  |  | 3.71E−08 | 214.458 | 2.41E−13 | [37] | ✓ |
|  | *OsFBK1* | Os01g0659900 | rs74390 | 1 | 27079447 | 0.0953 | 3.3863 | -0.4628 | 0.1681 |  |  |  |  |  |  | 204.416 | 3.70E−08 | [4] | ✓ |
|  | *OsCCS52B* | Os01g0972900 | rs117379 | 1 | 43240002 | 0.2917 | 4.0455 | 0.2618 | 0.2639 |  |  |  |  |  |  | 287.323 | 3.70E−08 | [38] | ✓ |
|  | *FUWA* | Os02g0234200 | rs137622 | 2 | 7623771 | 0.0475 | 5.1705 | 0.6709 | 0.3783 |  |  |  |  |  |  | 23.903 | 1.33E−27 | [39] | ✓ |
|  |  |  | rs137755 | 2 | 7692509 | 0.3173 |  |  |  |  |  |  | 1.18E−08 |  |  | 92.641 |  |  |  |
|  |  |  | rs138092 | 2 | 7800124 | 0.3881 |  |  |  | 5.7165 | 0.6131 | 1.6631 |  |  |  | 200.256 |  |  |  |
|  | *OsPLIM2a* | Os02g0641000 | rs189023 | 2 | 25943490 | 0.1362 | 5.6016 | 0.5421 | 0.6905 |  |  |  |  |  |  | 202.657 | 6.76E−05 | [40] | ✓ |
|  |  |  | rs189668 | 2 | 26092847 | 0.2371 |  |  |  |  |  |  |  |  | 4.38E−08 | 352.014 |  |  |  |
|  | *OsMRP5* | Os03g0142800 | rs217372 | 3 | 2569221 | 0.0391 | 3.0001 | -0.5306 | 0.2102 | 3.5575 | -0.6306 | 0.2372 | 2.23E−08 |  |  | 194.784 | 4.67E−13 | [41] | ✓ |
|  | *BG1* | Os03g0175800 | rs219408 | 3 | 3756927 | 0.1194 |  |  |  | 4.9682 | 0.8006 | 0.9715 |  |  |  | 281.414 | 2.40E−05 | [42] | ✓ |
|  | *OsPUP1* | Os03g0187800 | rs220534 | 3 | 4368320 | 0.0487 | 3.2451 | -0.7274 | 0.3516 |  |  |  |  |  |  | 230.604 | 1.36E−04 | [43] | ✓ |
|  | *DG1* | Os03g0229500 | rs226238 | 3 | 6587019 | 0.1581 |  |  |  | 8.3582 | 0.991 | 2.3121 |  |  | 2.84E−08 | 266.772 | 1.40E−14 | [44] | ✓ |
|  | *LPA1* | Os03g0237250 | rs226979 | 3 | 6930244 | 0.4847 |  |  |  | 3.3425 | 0.4079 | 0.8019 |  |  |  | 306.918 | 2.25E−05 | [47] | ✓ |
|  | *GS3* | Os03g0407400 | rs250497 | 3 | 16696473 | 0.0137 |  |  |  |  |  |  | 1.17E−18 |  |  | 33.028 | 3.08E−13 | [48] |  |
|  |  |  | rs250624 | 3 | 16733441 | 0.3602 | 24.0141 | 0.9777 | 3.6694 | 28.1945 | 1.1614 | 5.2053 |  | 3.66E−11 | 3.55E−10 | 1.668 |  |  |  |
|  | *GL3.1* | Os03g0646900 | rs272978 | 3 | 24807654 | 0.1059 | 3.5869 | 0.5141 | 0.2374 |  |  |  |  |  |  | 234.773 | 1.51E−03 | [31] | ✓ |
|  | *RGB1* | Os03g0669200 | rs277085 | 3 | 26706426 | 0.3638 | 4.7309 | 0.3761 | 0.6165 |  |  |  |  |  |  | 299.934 | 2.06E−13 | [49] | ✓ |
|  | *OsINV2* | Os04g0535600 | rs372241 | 4 | 26852458 | 0.0902 | 3.6488 | 0.5272 | 0.2342 |  |  |  |  |  |  | 80.589 | 1.36E−09 | [50] | ✓ |
|  | *GSN1* | Os05g0115800 | rs396679 | 5 | 831332 | 0.2291 |  |  |  | 4.9257 | -0.3742 | 0.4187 |  |  |  | 27.067 | 2.00E−08 | [20] | ✓ |
|  | *GW5* | Os05g0187500 | rs408480 | 5 | 5007414 | 0.3768 | 8.9059 | -0.5827 | 1.1038 |  |  |  |  |  |  | 357.708 | 1.29E−04 | [22] | ✓ |
|  |  |  | rs409047 | 5 | 5363587 | 0.454 |  |  |  | 10.3202 | 0.5857 | 1.6463 |  | 3.60E−08 |  | 1.535 |  |  |  |
|  |  |  | rs409051 | 5 | 5365256 | 0.3757 |  |  |  |  |  |  | 3.69E−11 |  |  | 0.134 |  |  |  |
|  | *OsDER1* | Os05g0187800 | rs408898 | 5 | 5299051 | 0.1988 |  |  |  | 7.857 | -0.7748 | 1.5369 |  |  |  | 95.405 | 1.22E−13 | [21] | ✓ |
|  |  |  | rs409051 | 5 | 5365256 | 0.3757 |  |  |  |  |  |  | 3.69E−11 |  |  | 0.134 |  |  |  |
|  |  |  | rs409091 | 5 | 5383914 | 0.2125 |  |  |  |  |  |  |  | 1.63E−08 |  | 10.542 |  |  |  |
|  |  |  | rs409769 | 5 | 5617272 | 0.123 | 3.0422 | -0.3752 | 0.2379 |  |  |  |  |  |  | 219.227 |  |  |  |
|  | *OsGSK2* | Os05g0207500 | rs412981 | 5 | 6818314 | 0.0982 | 3.5983 | 0.4014 | 0.282 |  |  |  |  |  |  | 156.821 | 2.08E−05 | [51] | ✓ |
|  | *OsLAC* | Os05g0458600 | rs452380 | 5 | 22468025 | 0.207 | 6.38 | 0.4848 | 0.5813 |  |  |  |  |  |  | 65.511 | 1.45E−07 | [52] |  |
|  | *OsS40-14* | Os05g0531000 | rs459015 | 5 | 26365993 | 0.0252 |  |  |  |  |  |  |  | 3.34E−08 | 6.77E−09 | 0.398 | 4.23E−29 | [53] | ✓ |
|  | *SSG6* | Os06g0130400 | rs470654 | 6 | 1510781 | 0.1592 |  |  |  | 12.5252 | -0.9105 | 1.969 |  |  |  | 118.997 | 1.64E−07 | [54] | ✓ |
|  | *DSG1* | Os06g0154500 | rs472999 | 6 | 2577029 | 0.1473 | 4.318 | 0.4333 | 0.4671 |  |  |  |  |  |  | 229.639 | 1.91E−09 | [55] | ✓ |
|  |  |  | rs473055 | 6 | 2608798 | 0.1303 |  |  |  | 4.9554 | 0.6177 | 0.8821 |  |  |  | 197.87 |  |  |  |
|  | *OsUBR7* | Os06g0529800 | rs519253 | 6 | 19932046 | 0.1743 |  |  |  | 4.4211 | 0.7026 | 0.6198 |  |  |  | 262.504 | 9.08E−13 | [56] | ✓ |
|  | *OsGSK4* | Os06g0547900 | rs520850 | 6 | 20546753 | 0.4651 | 3.9321 | 0.3448 | 0.4327 | 5.1944 | 0.5645 | 1.3782 |  |  |  | 171.055 | 1.60E−09 | [2] | ✓ |
|  | *TGW6* | Os06g0623700 | rs533452 | 6 | 25081743 | 0.0057 |  |  |  |  |  |  | 6.41E−08 |  |  | 11.499 | 6.67E−04 | [57] | ✓ |
|  | *OsCYP19-4* | Os06g0708400 | rs546149 | 6 | 30066598 | 0.4312 |  |  |  | 3.4205 | 0.3763 | 0.6457 |  |  |  | 93.625 | 2.13E−07 | [58] | ✓ |
|  |  |  | rs546397 | 6 | 30171804 | 0.0312 | 3.8971 | 0.6755 | 0.2867 |  |  |  |  |  |  | 198.831 |  |  |  |
|  | *RAG2* | Os07g0214300 | rs559704 | 7 | 6168361 | 0.228 | 3.014 | 0.3272 | 0.2613 |  |  |  |  |  |  | 90.556 | 7.41E−03 | [59] | ✓ |
|  | *LC7* | Os07g0658400 | rs619861 | 7 | 27596731 | 0.3591 | 3.9196 | 0.2819 | 0.3304 |  |  |  |  |  |  | 126.358 | 1.46E−09 | [60] | ✓ |
|  | *FZP* | Os07g0669500 | rs621236 | 7 | 28319838 | 0.0812 | 13.7981 | -1.1926 | 2.1479 | 15.156 | -1.3705 | 2.7552 |  | 3.83E−12 | 2.71E−13 | 18.749 | 7.57E−11 | [61] | ✓ |
|  |  |  | rs621242 | 7 | 28323091 | 0.0831 |  |  |  |  |  |  | 9.11E−21 |  |  | 22.002 |  |  |  |
|  | *OsEIL2* | Os07g0685700 | rs622032 | 7 | 28762557 | 0.0801 | 6.8419 | -0.9577 | 0.58 |  |  |  |  |  |  | 353.501 | 5.56E−04 | [62] | ✓ |
|  | *Ghd7.1* | Os07g0695100 | rs623102 | 7 | 29368338 | 0.0389 | 6.254 | 1.1589 | 0.7388 |  |  |  |  |  |  | 248.367 | 1.85E−05 | [63] | ✓ |
|  | *OsCCC1* | Os08g0323700 | rs670843 | 8 | 14535813 | 0.1026 | 3.3126 | 0.5492 | 0.4574 |  |  |  |  |  |  | 340.393 | 4.41E−09 | [64] | ✓ |
|  | *IPA1* | Os08g0509600 | rs702329 | 8 | 24928701 | 0.0279 |  |  |  |  |  |  |  | 4.95E−08 | 1.74E−09 | 345.84 | 4.10E−07 | [65] | ✓ |
|  | *OsSHI1* | Os09g0531600 | rs769245 | 9 | 20921346 | 0.0431 | 3.1289 | -0.865 | 0.5452 |  |  |  |  | 2.26E−08 | 4.83E−09 | 81.417 | 5.35E−17 | [66] | ✓ |
|  | *YL3* | Os09g0552800 | rs770909 | 9 | 21757666 | 0.3569 | 3.4285 | -0.3847 | 0.5536 |  |  |  |  |  |  | 145.048 | 3.93E−05 | [67] | ✓ |
|  | *OsSCP46* | Os10g0101200 | rs773656 | 10 | 134175 | 0.4314 |  |  |  | 4.9424 | 0.4364 | 0.8483 |  |  |  | 17.723 | 1.72E−10 | [68] | ✓ |
|  |  |  | rs774268 | 10 | 398800 | 0.2105 | 3.1077 | -0.3462 | 0.3137 |  |  |  |  |  |  | 282.348 |  |  |  |
|  | *OsMADS56* | Os10g0536100 | rs833332 | 10 | 21100560 | 0.0887 | 7.0199 | -0.7163 | 0.7639 | 5.4997 | -0.7916 | 0.9192 |  |  |  | 226.925 | 5.68E−11 | [69] | ✓ |
|  | *OsSMK1* | Os11g0213500 | rs851224 | 11 | 6255324 | 0.0261 |  |  |  |  |  |  |  |  | 1.06E−08 | 358.157 | 1.03E−07 | [41] | ✓ |
|  | *SRS5* | Os11g0247300 | rs855595 | 11 | 7731678 | 0.3806 |  |  |  |  |  |  | 4.77E−09 |  |  | 228.853 | 4.38E−05 | [70] | ✓ |
|  | *SWEET14* | Os11g0508600 | rs890142 | 11 | 18042330 | 0.1101 | 6.7951 | 0.6876 | 0.5484 | 3.8641 | 0.6425 | 0.2857 |  |  |  | 129.377 | 6.22E−14 | [71] | ✓ |
|  | *MRG702* | Os11g0545600 | rs899035 | 11 | 20049196 | 0.1061 |  |  |  | 3.0479 | 0.472 | 0.3021 |  |  |  | 48.87 | 2.76E−02 | [33] | ✓ |

*Note*: RAP locus: Gene names. The known genes were confirmed by haplotype analysis, and *P* value of haplotype analysis is obtained from ANOVA for the traits of interest across various haplotypes. ATAC-seq dataset was derived from http://glab.hzau.edu.cn/RiceENCODE/, in which the genes with open chromosomal regions were marked by “✓”. GLWR, grain length width ratio; LOD, logarithm of odds.

**References**

1. Matsushima R, Maekawa M, Kusano M, Kondo H, Fujita N, Kawagoe Y, et al. Amyloplast-localized SUBSTANDARD STARCH GRAIN4 protein influences the size of starch grains in rice endosperm. Plant Physiol 2014;164:623–36.
2. Liu D, Yu Z, Zhang G, Yin W, Li L, Niu M, et al. Diversification of plant agronomic traits by genome editing of brassinosteroid signaling family genes in rice. Plant Physiol 2021;187:2563–76.
3. Liu Y, Chen X, Xue S, Quan T, Cui D, Han L, et al. SET DOMAIN GROUP 721 protein functions in saline-alkaline stress tolerance in the model rice variety Kitaake. Plant Biotechnol J 2021;19:2576–88.
4. Borna RS, Murchie EH, Pyke KA, Roberts JA, Gonzalez-Carranza ZH. The rice *EP3* and *OsFBK1 E3* ligases alter plant architecture and flower development, and affect transcript accumulation of microRNA pathway genes and their targets. Plant Biotechnol J 2022;20:297–309.
5. Yue ZL, Liu N, Deng ZP, Zhang Y, Wu ZM, Zhao JL, et al. The receptor kinase OsWAK11 monitors cell wall pectin changes to fine-tune brassinosteroid signaling and regulate cell elongation in rice. Curr Biol 2022;32: 2454–66e7.
6. Hao J, Wang D, Wu Y, Huang K, Duan P, Li N, et al. The GW2-WG1-OsbZIP47 pathway controls grain size and weight in rice. Mol Plant 2021;14:1266–80.
7. Tanaka N, Fujita N, Nishi A, Satoh H, Hosaka Y, Ugaki M, et al. The structure of starch can be manipulated by changing the expression levels of starch branching enzyme IIb in rice endosperm. Plant Biotechnol J 2004;2:507–16.
8. Jang S, An G, Li HY. Rice leaf angle and grain size are affected by the OsBUL1 transcriptional activator complex. Plant Physiol 2017;173:688–702.
9. Liu Q, Han R, Wu K, Zhang J, Ye Y, Wang S, et al. G-protein βγ betagamma subunits determine grain size through interaction with MADS-domain transcription factors in rice. Nat Commun 2018;9:852.
10. Hu X, Qian Q, Xu T, Zhang Y, Dong G, Gao T, et al. The U-box E3 ubiquitin ligase TUD1 functions with a heterotrimeric G α subunit to regulate Brassinosteroid-mediated growth in rice. PLoS Genet 2013;9: e1003391.
11. Liu C, Ma T, Yuan D, Zhou Y, Long Y, Li Z, et al. The OsEIL1-OsERF115-target gene regulatory module controls grain size and weight in rice. Plant Biotechnol J 2022;20:1470–86.
12. Fan C, Yu S, Wang C, Xing Y. A causal C-A mutation in the second exon of *GS3* highly associated with rice grain length and validated as a functional marker. Theor Appl Genet 2009;118:465–72.
13. Xu F, Fang J, Ou S, Gao S, Zhang F, Du L, et al. Variations in *CYP78A13* coding region influence grain size and yield in rice. Plant Cell Environ 2015;38:800–11.
14. Sun S, Wang L, Mao H, Shao L, Li X, Xiao J, et al. A G-protein pathway determines grain size in rice. Nat Commun 2018;9:851.
15. Zhang Z, Zhao H, Huang F, Long J, Song G, Lin W. The 14-3-3 protein GF14f negatively affects grain filling of inferior spikelets of rice (*Oryza sativa* L.). Plant J 2019;99:344–58.
16. Ying JZ, Ma M, Bai C, Huang XH, Liu JL, Fan YY, et al. *TGW3*, a major QTL that negatively modulates grain length and weight in rice. Mol Plant 2018;11:750–3.
17. Yin W, Xiao Y, Niu M, Meng W, Li L, Zhang X, et al. ARGONAUTE2 enhances grain length and salt tolerance by activating *BIG GRAIN3* to modulate cytokinin distribution in rice. Plant Cell 2020;32:2292–306.
18. Gui J, Liu C, Shen J, Li L. *Grain setting defect1*, encoding a remorin protein, affects the grain setting in rice through regulating plasmodesmatal conductance. Plant Physiol 2014;166:1463–78.
19. She KC, Kusano H, Koizumi K, Yamakawa H, Hakata M, Imamura T, et al. A novel factor *FLOURY ENDOSPERM2* is involved in regulation of rice grain size and starch quality. Plant Cell 2010;22:3280–94.
20. Guo T, Chen K, Dong NQ, Shi CL, Ye WW, Gao JP, et al. *GRAIN SIZE AND NUMBER1* negatively regulates the OsMKKK10-OsMKK4-OsMPK6 cascade to coordinate the trade-off between grain number per panicle and grain size in rice. Plant Cell 2018;30:871–88.
21. Qian D, Chen G, Tian L, Qu LQ. OsDER1 is an ER-associated protein degradation factor that responds to ER stress. Plant Physiol 2018;178:402–12.
22. Liu J, Chen J, Zheng X, Wu F, Lin Q, Heng Y, et al. *GW5* acts in the brassinosteroid signalling pathway to regulate grain width and weight in rice. Nat Plants 2017;3:17043.
23. Cui X, Jin P, Cui X, Gu L, Lu Z, Xue Y, et al. Control of transposon activity by a histone H3K4 demethylase in rice. Proc Natl Acad Sci U S A 2013;110:1953–8.
24. Tian Q, Shen L, Luan J, Zhou Z, Guo D, Shen Y, et al. Rice shaker potassium channel OsAKT2 positively regulates salt tolerance and grain yield by mediating K(+) redistribution. Plant Cell Environ 2021;44:2951–65.
25. Sun L, Li X, Fu Y, Zhu Z, Tan L, Liu F, et al. *GS6*, a member of the GRAS gene family, negatively regulates grain size in rice. J Integr Plant Biol 2013;55:938–49.
26. Guo T, Lu ZQ, Shan JX, Ye WW, Dong NQ, Lin HX. *ERECTA1* acts upstream of the OsMKKK10- OsMKK4-OsMPK6 cascade to control spikelet number by regulating cytokinin metabolism in rice. Plant Cell 2020;32:2763–79.
27. Wang A, Hou Q, Si L, Huang X, Luo J, Lu D, et al. The PLATZ transcription factor GL6 affects grain length and number in rice. Plant Physiol 2019;180:2077–90.
28. Zhang G, Song X, Guo H, Wu Y, Chen X, Fang R. A small G protein as a novel component of the rice brassinosteroid signal transduction. Mol Plant 2016;9:1260–71.
29. Wang S, Li S, Liu Q, Wu K, Zhang J, Wang S, et al. The *OsSPL16-GW7* regulatory module determines grain shape and simultaneously improves rice yield and grain quality. Nat Genet 2015;47:949–54.
30. Cheng X, Pan M, E Z, Zhou Y, Niu B, Chen C. Functional divergence of two duplicated fertilization independent endosperm genes in rice with respect to seed development. Plant J 2020;104:124–37.
31. Qi P, Lin YS, Song XJ, Shen JB, Huang W, Shan JX, et al. The novel quantitative trait locus *GL3.1* controls rice grain size and yield by regulating Cyclin-T1;3. Cell Res 2012;22:1666–80.
32. Hong Y, Liu Q, Cao Y, Zhang Y, Chen D, Lou X, et al. The *OsMPK15* negatively regulates m*agnaporthe* oryza and *Xoo d*isease resistance via SA and JA signaling pathway in rice. Front Plant Sci 2019;10:752.
33. Jin J, Shi J, Liu B, Liu Y, Huang Y, Yu Y, et al. MORF-RELATED GENE702, a reader protein of trimethylated histone H3 lysine 4 and histone H3 lysine 36, is involved in brassinosteroid-regulated growth and flowering time control in rice. Plant Physiol 2015;168:1275–85.
34. Cho SH, Yoo SC, Zhang H, Pandeya D, Koh HJ, Hwang JY, et al. The rice *narrow leaf2* and *narrow leaf3* loci encode WUSCHEL-related homeobox 3A (OsWOX3A) and function in leaf, spikelet, tiller and lateral root development. New Phytol 2013;198:1071–84.
35. Zhang H, Lu Y, Zhao Y, Zhou DX. OsSRT1 is involved in rice seed development through regulation of starch metabolism gene expression. Plant Sci 2016;248:28–36.
36. Fang N, Xu R, Huang L, Zhang B, Duan P, Li N, et al. *SMALL GRAIN* 11 controls grain size, grain number and grain yield in rice. Rice 2016;9:64.
37. Kong W, Yu X, Chen H, Liu L, Xiao Y, Wang Y, et al. The catalytic subunit of magnesium-protoporphyrin IX monomethyl ester cyclase forms a chloroplast complex to regulate chlorophyll biosynthesis in rice. Plant Mol Biol 2016;92:177–91.
38. Su’udi M, Cha JY, Ahn IP, Kwak YS, Woo YM, Son D. Functional characterization of a B-type cell cycle switch 52 in rice (*OsCCS52B*). Plant Cell Tiss Org 2012;111:101–11.
39. Chen J, Gao H, Zheng XM, Jin M, Weng JF, Ma J, et al. An evolutionarily conserved gene, *FUWA*, plays a role in determining panicle architecture, grain shape and grain weight in rice. Plant J 2015;83:427–38.
40. Na JK, Huh SM, Yoon IS, Byun MO, Lee YH, Lee KO, et al. Rice LIM protein OsPLIM2a is involved in rice seed and tiller development. Mol Breeding 2014;34:569–81.
41. Li XJ, Zhang YF, Hou M, Sun F, Shen Y, Xiu ZH, et al. *Small kernel 1* encodes a pentatricopeptide repeat protein required for mitochondrial nad7 transcript editing and seed development in maize (*Zea mays*) and rice (*Oryza sativa*). Plant J 2014;79:797–809.
42. Liu L, Tong H, Xiao Y, Che R, Xu F, Hu B, et al. Activation of Big Grain1 significantly improves grain size by regulating auxin transport in rice. Proc Natl Acad Sci U S A 2015;112:11102–7.
43. Xiao Y, Zhang J, Yu G, Lu X, Mei W, Deng H, et al. Endoplasmic reticulum-localized PURINE PERMEASE1 regulates plant height and grain weight by modulating cytokinin distribution in rice. Front Plant Sci 2020;11:618560.
44. Qin P, Zhang G, Hu B, Wu J, Chen W, Ren Z, et al. Leaf-derived ABA regulates rice seed development via a transporter- mediated and temperature-sensitive mechanism. Sci Adv 2021;7:eabc8873.
45. Zhang J, Gao X, Cai G, Wang Y, Li J, Du H, et al. An adenylate kinase OsAK3 involves brassinosteroid signaling and grain length in rice (*Oryza sativa* L.). Rice 2021;14:105.
46. Li WX, Zhao HJ, Pang WQ, Cui HR, Poirier Y, Shu QY. Seed-specific silencing of *OsMRP5* reduces seed phytic acid and weight in rice. Transgenic Res 2014;23:585–99.
47. Sun Q, Li TY, Li DD, Wang ZY, Li S, Li DP, et al. Overexpression of *Loose Plant Architecture 1* increases planting density and resistance to sheath blight disease via activation of *PIN-FORMED 1a* in rice. Plant Biotechnol J 2019;17:855–7.
48. Fan C, Xing Y, Mao H, Lu T, Han B, Xu C, et al. *GS3*, a major QTL for grain length and weight and minor QTL for grain width and thickness in rice, encodes a putative transmembrane protein. Theor Appl Genet 2006;112:1164–71.
49. Zhang D, Zhang M, Liang J. RGB1 regulates grain development and starch accumulation through its effect on *OsYUC11*-mediated auxin biosynthesis in rice endosperm cells. Front Plant Sci 2021;12:585174.
50. Deng X, Han X, Yu S, Liu Z, Guo D, He Y, et al. *OsINV3* and its homolog, *OsINV2*, control grain size in rice. Int J Mol Sci 2020;21:2199.
51. Xiao Y, Zhang G, Liu D, Niu M, Tong H, Chu C. GSK2 stabilizes OFP3 to suppress brassinosteroid responses in rice. Plant J 2020;102:1187–201.
52. Zhang YC, Yu Y, Wang CY, Li ZY, Liu Q, Xu J, et al. Overexpression of microRNA OsmiR397 improves rice yield by increasing grain size and promoting panicle branching. Nat Biotechnol 2013;31:848–52.
53. Habiba, Xu J, Gad AG, Luo Y, Fan C, Uddin JBG, et al. Five *OsS40* family members are identified as senescence-related genes in rice by reverse genetics approach. Front Plant Sci 2021;12:701529.
54. Matsushima R, Maekawa M, Kusano M, Tomita K, Kondo H, Nishimura H, et al. Amyloplast membrane protein SUBSTANDARD STARCH GRAIN6 controls starch grain size in rice endosperm. Plant Physiol 2016;170:1445–59.
55. Liu S, Hua L, Dong S, Chen H, Zhu X, Jiang J, et al. OsMAPK6, a mitogen-activated protein kinase, influences rice grain size and biomass production. Plant J 2015;84:672–81.
56. Zheng Y, Zhang S, Luo Y, Li F, Tan J, Wang B, et al. Rice OsUBR7 modulates plant height by regulating histone H2B monoubiquitination and cell proliferation. Plant Commun 2022;3:100412.
57. Ishimaru K, Hirotsu N, Madoka Y, Murakami N, Hara N, Onodera H, et al. Loss of function of the IAA-glucose hydrolase gene *TGW6* enhances rice grain weight and increases yield. Nat Genet 2013;45:707–11.
58. Yoon DH, Lee SS, Park HJ, Lyu JI, Chong WS, Liu JR, et al. Overexpression of *OsCYP19-4* increases tolerance to cold stress and enhances grain yield in rice (*Oryza sativa*). J Exp Bot 2016;67:69–82.
59. Zhou W, Wang X, Zhou D, Ouyang Y, Yao J. Overexpression of the 16-kDa α-amylase/trypsin inhibitor RAG2 improves grain yield and quality of rice. Plant Biotechnol J 2017;15:568–80.
60. Sun L, Wang Y, Liu LL, Wang C, Gan T, Zhang Z, et al. Isolation and characterization of a *spotted leaf 32* mutant with early leaf senescence and enhanced defense response in rice. Sci Rep 2017;7:41846.
61. Ren D, Hu J, Xu Q, Cui Y, Zhang Y, Zhou T, et al. *FZP* determines grain size and sterile lemma fate in rice. J Exp Bot 2018;69:4853–66.
62. Yang C, Ma B, He SJ, Xiong Q, Duan KX, Yin CC, et al. *MAOHUZI6/ETHYLENE INSENSITIVE3-LIKE1* and *ETHYLENE INSENSITIVE3-LIKE2* regulate ethylene response of roots and coleoptiles and negatively affect salt tolerance in rice. Plant Physiol 2015;169:148–65.
63. Wei X, Qiu J, Yong K, Fan J, Zhang Q, Hua H, et al. A quantitative genomics map of rice provides genetic insights and guides breeding. Nat Genet 2021;53:243–53.
64. Chen ZC, Yamaji N, Fujii-Kashino M, Ma JF. A cation-chloride cotransporter gene is required for cell elongation and osmoregulation in rice. Plant Physiol 2016;171:494–507.
65. Jiao Y, Wang Y, Xue D, Wang J, Yan M, Liu G, et al. Regulation of *OsSPL14* by OsmiR156 defines ideal plant architecture in rice. Nat Genet 2010;42:541–4.
66. Duan E, Wang Y, Li X, Lin Q, Zhang T, Wang Y, et al. OsSHI1 regulates plant architecture through modulating the transcriptional activity of IPA1 in rice. Plant Cell 2019;31:1026–42.
67. Li L, He Y, Zhang Z, Shi Y, Zhang X, Xu X, et al. OsNAC109 regulates senescence, growth and development by altering the expression of senescence- and phytohormone-associated genes in rice. Plant Mol Biol 2021;105:637–54.
68. Li Z, Tang L, Qiu J, Zhang W, Wang Y, Tong X, et al. *Serine carboxypeptidase 46* regulates grain filling and seed germination in rice (*Oryza sativa* L.). PLoS One 2016;11:e0159737.
69. Zhan P, Ma S, Xiao Z, Li F, Wei X, Lin S, et al. Natural variations in *grain length 10 (GL10)* regulate rice grain size. J Genet Genomics 2022;49:405–13.
70. Segami S, Kono I, Ando T, Yano M, Kitano H, Miura K, et al. *Small and round seed 5* gene encodes alpha-tubulin regulating seed cell elongation in rice. Rice 2012;5:4.
71. Kim P, Xue CY, Song HD, Gao Y, Feng L, Li Y, et al. Tissue-specific activation of *DOF11* promotes rice resistance to sheath blight disease and increases grain weight via activation of *SWEET14*. Plant Biotechnol J 2021;19:409–11.
