# Supplementary material for: BLUPmrMLM: A Fast mrMLM Algorithm in Genome-wide Association Studies
Source: Genomics Proteomics Bioinformatics. 2024 Feb 29;22(3):qzae020. doi: 10.1093/gpbjnl/qzae020 (PMC12016565; doi:10.1093/gpbjnl/qzae020)
Supplement: qzae020_Supplementary_Data [file qzae020_supplementary_data.zip › Table S6.docx]

**Table S6**  **MSE of QTN effects in four simulation experiments using different methods**

| **Experiment** | **Method** | **MSE for QTN effects** | | | | | | | | | | **Average** |
| --- | --- | --- | --- | --- | --- | --- | --- | --- | --- | --- | --- | --- |
|  |  | **1** | **2** | **3** | **4** | **5** | **6** | **7** | **8** | **9** | **10** |  |
| Ⅰ | BLUPmrMLM | 0.0872 | 0.175 | 0.1318 | 0.0887 | 0.1069 | 0.1034 | 0.1619 | 0.1088 | 0.0991 | 0.084 | 0.1147 |
|  | mrMLM | 0.2162 | 0.148 | 0.1529 | 0.0885 | 0.1292 | 0.2473 | 0.1489 | 0.0783 | 0.0556 | 0.1074 | 0.1372 |
|  | Control | 0.1559 | 0.1019 | 0.1317 | 0.0945 | 0.195 | 0.1961 | 0.1777 | 0.0994 | 0.08 | 0.1046 | 0.1337 |
|  | FarmCPU | 0.2062 | 1.6873 | 0.0919 | 0.0656 | 0.0885 | 0.198 | 0.1674 | 0.1113 | 0.0699 | 0.0452 | 0.2731 |
|  | GEMMA | 1.9466 | 3.7123 | 0.6856 | 0.5715 | 0.5369 | 1.6704 | 0.5254 | 0.4511 | 0.8346 | 0.864 | 1.1798 |
|  | EMMAX | 1.986 | 3.8651 | 0.6973 | 0.5903 | 0.5308 | 1.6752 | 0.5088 | 0.4598 | 0.8428 | 0.8451 | 1.2001 |
| Ⅱ | BLUPmrMLM | 0.1086 | 0.2476 | 0.1456 | 0.1074 | 0.1115 | 0.0986 | 0.1662 | 0.1418 | 0.1241 | 0.1237 | 0.1375 |
|  | mrMLM | 0.1967 | 0.1459 | 0.1701 | 0.0974 | 0.1273 | 0.229 | 0.1475 | 0.0921 | 0.073 | 0.1325 | 0.1412 |
|  | Control | 0.1579 | 0.1201 | 0.1561 | 0.1164 | 0.1326 | 0.1827 | 0.1638 | 0.1246 | 0.1001 | 0.1184 | 0.1373 |
|  | FarmCPU | 0.206 | 1.7324 | 0.1001 | 0.0724 | 0.0989 | 0.1911 | 0.1914 | 0.1573 | 0.068 | 0.0738 | 0.2892 |
|  | GEMMA | 2.261 | 2.7206 | 0.7406 | 0.6489 | 0.6122 | 1.8691 | 0.5744 | 0.4903 | 1.0001 | 1.1367 | 1.2054 |
|  | EMMAX | 2.3095 | 2.6843 | 0.7542 | 0.6688 | 0.5967 | 1.8653 | 0.5565 | 0.4955 | 0.9912 | 1.1178 | 1.204 |
| Ⅲ | BLUPmrMLM | 0.1052 | 0.5442 | 0.1329 | 0.1188 | 0.1172 | 0.1323 | 0.1541 | 0.1328 | 0.1602 | 0.1152 | 0.1713 |
|  | mrMLM | 0.1683 | 0.1906 | 0.1529 | 0.078 | 0.1742 | 0.3326 | 0.161 | 0.0956 | 0.046 | 0.1145 | 0.1514 |
|  | Control | 0.1051 | 0.1183 | 0.1453 | 0.0785 | 0.1699 | 0.253 | 0.1789 | 0.1141 | 0.1053 | 0.0945 | 0.1363 |
|  | FarmCPU | 0.2008 | 2.615 | 0.1067 | 0.0603 | 0.097 | 0.2102 | 0.1822 | 0.1375 | 0.0351 | 0.1069 | 0.3752 |
|  | GEMMA | 2.1011 | 4.6918 | 0.8235 | 0.6777 | 0.6575 | 2.1102 | 0.7944 | 0.5292 | -- | 1.1225 | 1.3508 |
|  | EMMAX | 2.1216 | 4.1829 | 0.848 | 0.717 | 0.6318 | 2.11 | 0.7721 | 0.5424 | -- | 1.1187 | 1.3045 |
| Ⅳ | BLUPmrMLM | 0.1139 | 0.5475 | 0.1514 | 0.1501 | 0.1315 | 0.1113 | 0.1661 | 0.1778 | 0.2175 | 0.1367 | 0.1904 |
|  | mrMLM | 0.1804 | 0.1726 | 0.1866 | 0.095 | 0.1847 | 0.2616 | 0.1869 | 0.1136 | 0.0657 | 0.1298 | 0.1577 |
|  | Control | 0.1296 | 0.1514 | 0.1713 | 0.1229 | 0.1537 | 0.1787 | 0.2346 | 0.1352 | 0.1759 | 0.1109 | 0.1564 |
|  | FarmCPU | 0.188 | 3.3442 | 0.1139 | 0.0795 | 0.12 | 0.1902 | 0.1903 | 0.1779 | 0.0399 | 0.1094 | 0.4553 |
|  | GEMMA | 2.6424 | 3.9929 | 0.8512 | 0.7439 | 0.7335 | 2.2882 | 0.9563 | 0.5706 | 0.9279 | 1.2826 | 1.4989 |
|  | EMMAX | 2.5944 | 3.9371 | 0.8894 | 0.7925 | 0.7025 | 2.2867 | 0.9304 | 0.5821 | 0.9068 | 1.2597 | 1.4882 |

*Note*: *MSE*, mean squared error; *QTN*, quantitative trait nucleotide.
