# Supplementary material for: BLUPmrMLM: A Fast mrMLM Algorithm in Genome-wide Association Studies
Source: Genomics Proteomics Bioinformatics. 2024 Feb 29;22(3):qzae020. doi: 10.1093/gpbjnl/qzae020 (PMC12016565; doi:10.1093/gpbjnl/qzae020)
Supplement: qzae020_Supplementary_Data [file qzae020_supplementary_data.zip › Table S3.docx]

**Table S3 Five pairs of epistatic QTNs in simulation experiment Ⅳ**

| **Epistatic QTNs** | **Locus 1 (bp)** | **Chromosome** | **Locus 2 (bp)** | **Chromosome** | **r^2^ (%)** |
| --- | --- | --- | --- | --- | --- |
| 1 | 19,915,324 | 1 | 37,318,725 | 5 | 2 |
| 2 | 30,848,016 | 5 | 28,081,335 | 6 | 2 |
| 3 | 25,431,037 | 2 | 29,807,763 | 8 | 2 |
| 4 | 34,720,101 | 7 | 405,577,938 | 7 | 2 |
| 5 | 27,045,208 | 4 | 27,588,335 | 4 | 2 |

*Note*: *QTN*, quantitative trait nucleotide.
