# Supplementary material for: BLUPmrMLM: A Fast mrMLM Algorithm in Genome-wide Association Studies
Source: Genomics Proteomics Bioinformatics. 2024 Feb 29;22(3):qzae020. doi: 10.1093/gpbjnl/qzae020 (PMC12016565; doi:10.1093/gpbjnl/qzae020)
Supplement: qzae020_Supplementary_Data [file qzae020_supplementary_data.zip › Table S7.docx]

**Table S7** **MAD of QTN effects in four simulation experiments using different methods**

| **Experiment** | **Method** | **MAD for QTN effects** | | | | | | | | | | **Average** |
| --- | --- | --- | --- | --- | --- | --- | --- | --- | --- | --- | --- | --- |
|  |  | **1** | **2** | **3** | **4** | **5** | **6** | **7** | **8** | **9** | **10** |  |
| Ⅰ | BLUPmrMLM | 0.2414 | 0.2693 | 0.2897 | 0.2467 | 0.2613 | 0.2454 | 0.3208 | 0.2645 | 0.2671 | 0.2318 | 0.2638 |
|  | mrMLM | 0.3782 | 0.2838 | 0.3099 | 0.233 | 0.2853 | 0.438 | 0.3071 | 0.2203 | 0.1842 | 0.2552 | 0.2895 |
|  | Control | 0.3046 | 0.2501 | 0.2944 | 0.2575 | 0.3035 | 0.3611 | 0.3237 | 0.2563 | 0.2347 | 0.263 | 0.2849 |
|  | FarmCPU | 0.3695 | 0.8193 | 0.2327 | 0.1994 | 0.2439 | 0.3894 | 0.3252 | 0.2875 | 0.1978 | 0.1521 | 0.3217 |
|  | GEMMA | 1.3918 | 1.6764 | 0.8125 | 0.7459 | 0.689 | 1.282 | 0.6451 | 0.6412 | 0.9075 | 0.9235 | 0.9715 |
|  | EMMAX | 1.4058 | 1.7161 | 0.8186 | 0.7587 | 0.6855 | 1.2837 | 0.6324 | 0.6484 | 0.9117 | 0.9142 | 0.9775 |
| Ⅱ | BLUPmrMLM | 0.2719 | 0.3222 | 0.3037 | 0.2704 | 0.2681 | 0.2411 | 0.3259 | 0.3042 | 0.3 | 0.2875 | 0.2895 |
|  | mrMLM | 0.3381 | 0.2962 | 0.3295 | 0.249 | 0.2891 | 0.414 | 0.3035 | 0.2434 | 0.2134 | 0.2772 | 0.2953 |
|  | Control | 0.3159 | 0.2769 | 0.3149 | 0.2729 | 0.2945 | 0.3485 | 0.3249 | 0.2855 | 0.2617 | 0.2738 | 0.297 |
|  | FarmCPU | 0.3605 | 0.8143 | 0.254 | 0.2177 | 0.2561 | 0.3869 | 0.3469 | 0.342 | 0.1962 | 0.2144 | 0.3389 |
|  | GEMMA | 1.4959 | 1.5218 | 0.843 | 0.7902 | 0.738 | 1.3566 | 0.6817 | 0.6646 | 0.9916 | 1.0563 | 1.014 |
|  | EMMAX | 1.5122 | 1.5088 | 0.8509 | 0.8046 | 0.7286 | 1.3554 | 0.6684 | 0.6681 | 0.9861 | 1.0488 | 1.0132 |
| Ⅲ | BLUPmrMLM | 0.2723 | 0.4243 | 0.2921 | 0.2806 | 0.2729 | 0.2829 | 0.3145 | 0.2911 | 0.3427 | 0.2772 | 0.3051 |
|  | mrMLM | 0.3232 | 0.3503 | 0.3105 | 0.2228 | 0.3411 | 0.5042 | 0.3205 | 0.2449 | 0.1731 | 0.2638 | 0.3054 |
|  | Control | 0.2626 | 0.2799 | 0.3086 | 0.2318 | 0.3357 | 0.4118 | 0.3368 | 0.272 | 0.2738 | 0.2465 | 0.2959 |
|  | FarmCPU | 0.3621 | 1.0682 | 0.2569 | 0.2006 | 0.2551 | 0.4194 | 0.341 | 0.3191 | 0.1466 | 0.2332 | 0.3602 |
|  | GEMMA | 1.4469 | 1.9183 | 0.8939 | 0.8129 | 0.7709 | 1.4433 | 0.8246 | 0.6975 | -- | 1.0542 | 0.9863 |
|  | EMMAX | 1.4542 | 1.8179 | 0.9078 | 0.8384 | 0.755 | 1.4433 | 0.8119 | 0.7066 | -- | 1.0533 | 0.9788 |
| Ⅳ | BLUPmrMLM | 0.2862 | 0.43 | 0.3155 | 0.322 | 0.2876 | 0.2557 | 0.3258 | 0.3409 | 0.4126 | 0.3057 | 0.3282 |
|  | mrMLM | 0.3275 | 0.3176 | 0.352 | 0.2464 | 0.3509 | 0.4393 | 0.3435 | 0.2679 | 0.2107 | 0.2805 | 0.3137 |
|  | Control | 0.292 | 0.3137 | 0.3375 | 0.2778 | 0.3176 | 0.3308 | 0.3642 | 0.2921 | 0.3692 | 0.2677 | 0.3163 |
|  | FarmCPU | 0.3389 | 1.2217 | 0.2806 | 0.2297 | 0.2824 | 0.3907 | 0.3457 | 0.3652 | 0.1688 | 0.2511 | 0.3875 |
|  | GEMMA | 1.6219 | 1.8071 | 0.9092 | 0.8492 | 0.8158 | 1.5041 | 0.9095 | 0.7207 | 0.9633 | 1.1231 | 1.1224 |
|  | EMMAX | 1.6075 | 1.7937 | 0.9311 | 0.8785 | 0.7992 | 1.5038 | 0.8957 | 0.7283 | 0.9523 | 1.1134 | 1.1204 |

*Note*: *MAD*, mean absolute deviation; *QTN*, quantitative trait nucleotide.
