# Supplementary material for: BLUPmrMLM: A Fast mrMLM Algorithm in Genome-wide Association Studies
Source: Genomics Proteomics Bioinformatics. 2024 Feb 29;22(3):qzae020. doi: 10.1093/gpbjnl/qzae020 (PMC12016565; doi:10.1093/gpbjnl/qzae020)
Supplement: qzae020_Supplementary_Data [file qzae020_supplementary_data.zip › Table S10.docx]

**Table S10**  **MAD of QTN positions in four simulation experiments using different methods**

| **Experiment** | **Method** | **MAD for QTN position** | | | | | | | | | | **Average** |
| --- | --- | --- | --- | --- | --- | --- | --- | --- | --- | --- | --- | --- |
|  |  | **1** | **2** | **3** | **4** | **5** | **6** | **7** | **8** | **9** | **10** |  |
| Ⅰ | BLUPmrMLM | 0.0000 | 0.0118 | 0.0000 | 0.0000 | 0.0000 | 0.0000 | 0.0000 | 0.0000 | 0.0000 | 0.1517 | 0.0164 |
|  | mrMLM | 0.0000 | 0.0023 | 0.0000 | 0.0000 | 0.0000 | 0.0000 | 0.0000 | 0.0000 | 0.0000 | 0.0066 | 0.0009 |
|  | Control | 0.0000 | 0.0000 | 0.0000 | 0.0000 | 0.0078 | 0.0000 | 0.0015 | 0.0000 | 0.0000 | 0.0082 | 0.0018 |
|  | FarmCPU | 0.0000 | 0.1539 | 0.0000 | 0.0000 | 0.0000 | 0.0000 | 0.0000 | 0.0000 | 0.0000 | 0.0106 | 0.1645 |
|  | GEMMA | 0.0000 | 0.1520 | 0.0000 | 0.0000 | 0.0000 | 0.0000 | 0.0000 | 0.0000 | 0.0000 | 0.0000 | 0.0152 |
|  | EMMAX | 0.0000 | 0.1609 | 0.0000 | 0.0000 | 0.0000 | 0.0000 | 0.0000 | 0.0000 | 0.0000 | 0.0000 | 0.0161 |
| Ⅱ | BLUPmrMLM | 0.0000 | 0.0143 | 0.0000 | 0.0000 | 0.0000 | 0.0000 | 0.0000 | 0.0000 | 0.0000 | 0.1925 | 0.0207 |
|  | mrMLM | 0.0000 | 0.0000 | 0.0000 | 0.0000 | 0.0000 | 0.0000 | 0.0000 | 0.0000 | 0.0000 | 0.0088 | 0.0008 |
|  | Control | 0.0000 | 0.0000 | 0.0000 | 0.0000 | 0.0000 | 0.0000 | 0.0000 | 0.0000 | 0.0000 | 0.0159 | 0.0016 |
|  | FarmCPU | 0.0000 | 0.1368 | 0.0000 | 0.0000 | 0.0000 | 0.0000 | 0.0000 | 0.0000 | 0.0000 | 0.0089 | 0.1457 |
|  | GEMMA | 0.0000 | 0.0595 | 0.0000 | 0.0000 | 0.0000 | 0.0000 | 0.0000 | 0.0000 | 0.0000 | 0.0000 | 0.0060 |
|  | EMMAX | 0.0000 | 0.0595 | 0.0000 | 0.0000 | 0.0000 | 0.0000 | 0.0000 | 0.0000 | 0.0000 | 0.0000 | 0.0060 |
| Ⅲ | BLUPmrMLM | 0.0000 | 0.0369 | 0.0000 | 0.0000 | 0.0000 | 0.0000 | 0.0000 | 0.0000 | 0.0000 | 0.0219 | 0.0059 |
|  | mrMLM | 0.0000 | 0.0000 | 0.0000 | 0.0000 | 0.0000 | 0.0000 | 0.0000 | 0.0000 | 0.0000 | 0.0000 | 0.0000 |
|  | Control | 0.0000 | 0.0000 | 0.0000 | 0.0000 | 0.0000 | 0.0000 | 0.0000 | 0.0000 | 0.0000 | 0.0000 | 0.0000 |
|  | FarmCPU | 0.0000 | 0.1822 | 0.0000 | 0.0000 | 0.0000 | 0.0000 | 0.0000 | 0.0000 | 0.0000 | 0.0000 | 0.0182 |
|  | GEMMA | 0.0000 | 0.1520 | 0.0000 | 0.0000 | 0.0000 | 0.0000 | 0.0000 | 0.0000 | 0.0000 | 0.0000 | 0.0152 |
|  | EMMAX | 0.0000 | 0.1244 | 0.0000 | 0.0000 | 0.0000 | 0.0000 | 0.0000 | 0.0000 | 0.0000 | 0.0000 | 0.0124 |
| Ⅳ | BLUPmrMLM | 0.0000 | 0.0315 | 0.0000 | 0.0000 | 0.0000 | 0.0000 | 0.0000 | 0.0000 | 0.0000 | 0.0257 | 0.0572 |
|  | mrMLM | 0.0000 | 0.0000 | 0.0000 | 0.0000 | 0.0000 | 0.0000 | 0.0000 | 0.0000 | 0.0000 | 0.0000 | 0.0000 |
|  | Control | 0.0000 | 0.0000 | 0.0000 | 0.0000 | 0.0000 | 0.0000 | 0.0014 | 0.0000 | 0.0000 | 0.0000 | 0.0001 |
|  | FarmCPU | 0.0000 | 0.2064 | 0.0000 | 0.0000 | 0.0000 | 0.0000 | 0.0000 | 0.0000 | 0.0000 | 0.0000 | 0.0206 |
|  | GEMMA | 0.0000 | 0.0805 | 0.0000 | 0.0000 | 0.0000 | 0.0000 | 0.0000 | 0.0000 | 0.0000 | 0.0000 | 0.0081 |
|  | EMMAX | 0.0000 | 0.0805 | 0.0000 | 0.0000 | 0.0000 | 0.0000 | 0.0000 | 0.0000 | 0.0000 | 0.0000 | 0.0081 |

*Note*: *MAD*, mean absolute deviation; *QTN*, quantitative trait nucleotide.
