# Supplementary material for: BLUPmrMLM: A Fast mrMLM Algorithm in Genome-wide Association Studies
Source: Genomics Proteomics Bioinformatics. 2024 Feb 29;22(3):qzae020. doi: 10.1093/gpbjnl/qzae020 (PMC12016565; doi:10.1093/gpbjnl/qzae020)
Supplement: qzae020_Supplementary_Data [file qzae020_supplementary_data.zip › Table S8.docx]

**Table S8**  **Average estimates for QTN effects in four simulation experiments using different methods**

| **Experiment** | **Method** | **QTN estimated effect value** | | | | | | | | | |
| --- | --- | --- | --- | --- | --- | --- | --- | --- | --- | --- | --- |
|  |  | **1** | **2** | **3** | **4** | **5** | **6** | **7** | **8** | **9** | **10** |
| Ⅰ | True value | 1.3194 | 1.3558 | –1.6550 | 1.3613 | –1.7158 | –0.9405 | 2.4383 | 1.7178 | –1.2654 | 1.3558 |
|  | BLUPmrMLM | 1.3099 | 1.2190 | –1.5912 | 1.2756 | –1.6698 | –1.1302 | 2.3051 | 1.5558 | –1.0915 | 1.3235 |
|  | mrMLM | 1.6753 | 1.5927 | –1.8431 | 1.5077 | –1.8938 | –1.3784 | 2.5015 | 1.721 | –1.3331 | 1.5359 |
|  | Control | 1.534 | 1.4097 | –1.6937 | 1.4431 | –1.8508 | –1.2896 | 2.4482 | 1.6536 | –1.1546 | 1.3546 |
|  | FarmCPU | 1.6865 | 0.9763 | –1.6610 | 1.4075 | –1.6705 | –1.3299 | 2.3331 | 1.5054 | –1.3857 | 1.3708 |
|  | GEMMA | 2.7112 | 1.5029 | –2.4675 | 2.1072 | –2.4048 | –2.2225 | 3.0801 | 2.359 | –2.1729 | 2.2793 |
|  | EMMAX | 2.7252 | 1.4544 | –2.4736 | 2.12 | –2.4013 | –2.2242 | 3.0672 | 2.3662 | –2.1771 | 2.2700 |
| Ⅱ | True effect | 1.4454 | 1.4852 | –1.8129 | 1.4913 | –1.8796 | –1.0303 | 2.6711 | 1.8818 | –1.3862 | 1.4852 |
|  | BLUPmrMLM | 1.4253 | 1.3372 | –1.7447 | 1.3849 | –1.8231 | –1.201 | 2.5116 | 1.6978 | –1.1949 | 1.425 |
|  | mrMLM | 1.7397 | 1.7279 | –1.9693 | 1.597 | –2.0189 | –1.4423 | 2.6937 | 1.8709 | –1.4347 | 1.6454 |
|  | Control | 1.5743 | 1.4795 | –1.8231 | 1.5446 | –1.992 | –1.3551 | 2.6384 | 1.7855 | –1.2787 | 1.4654 |
|  | FarmCPU | 1.794 | 1.1511 | –1.7904 | 1.5131 | –1.8275 | –1.4172 | 2.5304 | 1.6352 | –1.461 | 1.5131 |
|  | GEMMA | 2.9413 | 2.3887 | –2.6559 | 2.2815 | –2.6176 | –2.3869 | 3.3492 | 2.5464 | –2.3778 | 2.5415 |
|  | EMMAX | 2.9576 | 2.3763 | –2.6638 | 2.2959 | –2.6082 | –2.3857 | 3.3359 | 2.5499 | –2.3723 | 2.534 |
| Ⅲ | True effect | 1.4752 | 1.5158 | –1.8503 | 1.522 | –1.9183 | –1.0515 | 2.7261 | 1.9206 | –1.4148 | 1.5158 |
|  | BLUPmrMLM | 1.3895 | 1.2596 | –1.7787 | 1.3335 | –1.8705 | –1.2742 | 2.6308 | 1.729 | –1.1 | 1.4266 |
|  | mrMLM | 1.7512 | 1.8184 | –2.0248 | 1.5826 | –2.1527 | –1.5552 | 2.8545 | 1.9369 | –1.3818 | 1.6834 |
|  | Control | 1.5319 | 1.5682 | –1.8029 | 1.4879 | –2.1166 | –1.4485 | 2.8185 | 1.8811 | –1.1691 | 1.4842 |
|  | FarmCPU | 1.8253 | 0.9558 | –1.8418 | 1.5227 | –1.8427 | –1.4709 | 2.6908 | 1.6936 | –1.455 | 1.6246 |
|  | GEMMA | 2.9221 | 1.7509 | –2.7442 | 2.3349 | –2.6892 | –2.4948 | 3.5498 | 2.6181 | -- | 2.57 |
|  | EMMAX | 2.9294 | 1.9559 | –2.7581 | 2.3604 | –2.6733 | –2.4948 | 3.5369 | 2.6272 | -- | 2.5691 |
| Ⅳ | True effect | 1.616 | 1.6605 | –2.0269 | 1.6673 | –2.1014 | –1.1519 | 2.9863 | 2.1039 | –1.5498 | 1.6605 |
|  | BLUPmrMLM | 1.4568 | 1.371 | –1.938 | 1.4481 | –2.0486 | –1.3469 | 2.8988 | 1.8422 | –1.159 | 1.5396 |
|  | mrMLM | 1.8497 | 1.9176 | –2.1983 | 1.7203 | –2.3332 | –1.5887 | 3.1167 | 2.0516 | –1.4681 | 1.8234 |
|  | Control | 1.6719 | 1.6394 | –1.9908 | 1.5995 | –2.269 | –1.4545 | 3.0436 | 2.0262 | –1.2282 | 1.637 |
|  | FarmCPU | 1.9442 | 0.9007 | –1.9749 | 1.6636 | –2.0037 | –1.5426 | 2.9693 | 1.8062 | –1.5217 | 1.7285 |
|  | GEMMA | 3.2379 | 2.5057 | –2.9361 | 2.5165 | –2.9172 | –2.656 | 3.8954 | 2.8246 | –2.5131 | 2.7836 |
|  | EMMAX | 3.2235 | 2.4972 | –2.958 | 2.5458 | –2.9006 | –2.6557 | 3.8815 | 2.8322 | –2.5021 | 2.7739 |

*Note*: *QTN*, quantitative trait nucleotide.
