# Supplementary material for: BLUPmrMLM: A Fast mrMLM Algorithm in Genome-wide Association Studies
Source: Genomics Proteomics Bioinformatics. 2024 Feb 29;22(3):qzae020. doi: 10.1093/gpbjnl/qzae020 (PMC12016565; doi:10.1093/gpbjnl/qzae020)
Supplement: qzae020_Supplementary_Data [file qzae020_supplementary_data.zip › Table S9.docx]

**Table S9** **MSEs of QTN positions in four simulation experiments using different methods**

| **Experiment** | **Method** | **MSE for QTN position （Kb^2^）** | | | | | | | | | | **Average** |
| --- | --- | --- | --- | --- | --- | --- | --- | --- | --- | --- | --- | --- |
|  |  | **1** | **2** | **3** | **4** | **5** | **6** | **7** | **8** | **9** | **10** |  |
| Ⅰ | BLUPmrMLM | 0.0000 | 0.0080 | 0.0000 | 0.0000 | 0.0000 | 0.0000 | 0.0000 | 0.0000 | 0.0000 | 0.1925 | 0.0201 |
|  | mrMLM | 0.0000 | 0.0016 | 0.0000 | 0.0000 | 0.0000 | 0.0000 | 0.0000 | 0.0000 | 0.0000 | 0.0084 | 0.0001 |
|  | Control | 0.0000 | 0.0000 | 0.0000 | 0.0000 | 0.0123 | 0.0000 | 0.0021 | 0.0000 | 0.0000 | 0.0104 | 0.0014 |
|  | FarmCPU | 0.0000 | 0.1053 | 0.0000 | 0.0000 | 0.0000 | 0.0000 | 0.0000 | 0.0000 | 0.0000 | 0.0134 | 0.0023 |
|  | GEMMA | 0.0000 | 0.1040 | 0.0000 | 0.0000 | 0.0000 | 0.0000 | 0.0000 | 0.0000 | 0.0000 | 0.0000 | 0.0104 |
|  | EMMAX | 0.0000 | 0.1101 | 0.0000 | 0.0000 | 0.0000 | 0.0000 | 0.0000 | 0.0000 | 0.0000 | 0.0000 | 0.0110 |
| Ⅱ | BLUPmrMLM | 0.0000 | 0.0098 | 0.0000 | 0.0000 | 0.0000 | 0.0000 | 0.0000 | 0.0000 | 0.0000 | 0.2443 | 0.0254 |
|  | mrMLM | 0.0000 | 0.0000 | 0.0000 | 0.0000 | 0.0000 | 0.0000 | 0.0000 | 0.0000 | 0.0000 | 0.0112 | 0.0011 |
|  | Control | 0.0000 | 0.0000 | 0.0000 | 0.0000 | 0.0000 | 0.0000 | 0.0000 | 0.0000 | 0.0000 | 0.0202 | 0.0020 |
|  | FarmCPU | 0.0000 | 0.0936 | 0.0000 | 0.0000 | 0.0000 | 0.0000 | 0.0000 | 0.0000 | 0.0000 | 0.0113 | 0.0105 |
|  | GEMMA | 0.0000 | 0.0407 | 0.0000 | 0.0000 | 0.0000 | 0.0000 | 0.0000 | 0.0000 | 0.0000 | 0.0000 | 0.0041 |
|  | EMMAX | 0.0000 | 0.0407 | 0.0000 | 0.0000 | 0.0000 | 0.0000 | 0.0000 | 0.0000 | 0.0000 | 0.0000 | 0.0041 |
| Ⅲ | BLUPmrMLM | 0.0000 | 0.0253 | 0.0000 | 0.0000 | 0.0000 | 0.0000 | 0.0000 | 0.0000 | 0.0000 | 0.0278 | 0.0053 |
|  | mrMLM | 0.0000 | 0.0000 | 0.0000 | 0.0000 | 0.0000 | 0.0000 | 0.0000 | 0.0000 | 0.0000 | 0.0000 | 0.0000 |
|  | Control | 0.0000 | 0.0000 | 0.0000 | 0.0000 | 0.0000 | 0.0000 | 0.0000 | 0.0000 | 0.0000 | 0.0000 | 0.0000 |
|  | FarmCPU | 0.0000 | 0.1246 | 0.0000 | 0.0000 | 0.0000 | 0.0000 | 0.0000 | 0.0000 | 0.0000 | 0.0000 | 0.0125 |
|  | GEMMA | 0.0000 | 0.1040 | 0.0000 | 0.0000 | 0.0000 | 0.0000 | 0.0000 | 0.0000 | 0.0000 | 0.0000 | 0.0104 |
|  | EMMAX | 0.0000 | 0.0851 | 0.0000 | 0.0000 | 0.0000 | 0.0000 | 0.0000 | 0.0000 | 0.0000 | 0.0000 | 0.0085 |
| Ⅳ | BLUPmrMLM | 0.0000 | 0.0216 | 0.0000 | 0.0000 | 0.0000 | 0.0000 | 0.0000 | 0.0000 | 0.0000 | 0.0326 | 0.0054 |
|  | mrMLM | 0.0000 | 0.0000 | 0.0000 | 0.0000 | 0.0000 | 0.0000 | 0.0000 | 0.0000 | 0.0000 | 0.0000 | 0.0000 |
|  | Control | 0.0000 | 0.0000 | 0.0000 | 0.0000 | 0.0000 | 0.0000 | 0.0021 | 0.0000 | 0.0000 | 0.0000 | 0.0002 |
|  | FarmCPU | 0.0000 | 0.1412 | 0.0000 | 0.0000 | 0.0000 | 0.0000 | 0.0000 | 0.0000 | 0.0000 | 0.0000 | 0.0141 |
|  | GEMMA | 0.0000 | 0.0550 | 0.0000 | 0.0000 | 0.0000 | 0.0000 | 0.0000 | 0.0000 | 0.0000 | 0.0000 | 0.0055 |
|  | EMMAX | 0.0000 | 0.0550 | 0.0000 | 0.0000 | 0.0000 | 0.0000 | 0.0000 | 0.0000 | 0.0000 | 0.0000 | 0.0055 |

*Note*: QTN, quantitative trait nucleotide.
