# Supplementary material for: BLUPmrMLM: A Fast mrMLM Algorithm in Genome-wide Association Studies
Source: Genomics Proteomics Bioinformatics. 2024 Feb 29;22(3):qzae020. doi: 10.1093/gpbjnl/qzae020 (PMC12016565; doi:10.1093/gpbjnl/qzae020)
Supplement: qzae020_Supplementary_Data [file qzae020_supplementary_data.zip › Table S5.docx]

**Table S5** **Statistical powers of QTN detection in four simulation experiments using different methods**

| **Experiment** | **Method** | **Powers of QTN detection** | | | | | | | | | | **Average** |
| --- | --- | --- | --- | --- | --- | --- | --- | --- | --- | --- | --- | --- |
|  |  | **1** | **2** | **3** | **4** | **5** | **6** | **7** | **8** | **9** | **10** |  |
|  | r^2^(%) | 3 | 3 | 5 | 5 | 7 | 2 | 10 | 7 | 4 | 4 |  |
| Ⅰ | BLUPmrMLM | 48.1 | 48.1 | 86.5 | 56.4 | 96.5 | 55.1 | 99.6 | 73.8 | 47.6 | 41.4 | 65.31 |
|  | mrMLM | 29.8 | 29.5 | 81.5 | 38.2 | 94.9 | 49.3 | 99.6 | 72.4 | 37.0 | 38.3 | 57.05 |
|  | Control | 26.3 | 32.8 | 62.2 | 22.0 | 60.1 | 35.7 | 98.3 | 59.9 | 30.5 | 31.0 | 45.88 |
|  | FarmCPU | 33.9 | 16.0 | 58.2 | 41.1 | 91.0 | 29.9 | 97.6 | 61.9 | 12.5 | 12.0 | 45.41 |
|  | GEMMA | 2.5 | 1.8 | 9.9 | 25.5 | 91.0 | 26.6 | 98.8 | 62.6 | 1.1 | 2.7 | 32.25 |
|  | EMMAX | 2.0 | 1.7 | 8.8 | 19.7 | 87.9 | 25.2 | 98.5 | 61.6 | 1.1 | 2.5 | 30.90 |
| Ⅱ | BLUPmrMLM | 56.0 | 55.4 | 89.8 | 59.0 | 97.5 | 55.0 | 98.8 | 76.5 | 53.2 | 47.3 | 68.85 |
|  | mrMLM | 40.3 | 35.5 | 87.1 | 43.4 | 96.5 | 53.1 | 99.3 | 75.1 | 46.8 | 43.3 | 62.04 |
|  | Control | 34.2 | 41.1 | 72.8 | 26.6 | 67.9 | 41.8 | 98.6 | 63.5 | 35.3 | 31.9 | 51.37 |
|  | FarmCPU | 39.1 | 19.0 | 67.5 | 46.8 | 95.0 | 34.2 | 99.6 | 66.7 | 14.9 | 14.3 | 49.71 |
|  | GEMMA | 3.4 | 2.3 | 11.5 | 25.9 | 93.5 | 30.6 | 99.4 | 69.7 | 2.0 | 5.3 | 34.36 |
|  | EMMAX | 3.0 | 2.3 | 10.7 | 21.1 | 91.2 | 29.7 | 99.3 | 69.1 | 1.9 | 4.7 | 33.30 |
| Ⅲ | BLUPmrMLM | 38.1 | 56.0 | 72.2 | 60.7 | 97.1 | 56.5 | 99.9 | 75.2 | 31.3 | 47.2 | 63.42 |
|  | mrMLM | 18.3 | 36.9 | 81.5 | 35.0 | 84.5 | 51.9 | 99.6 | 51.9 | 16.2 | 45.9 | 52.17 |
|  | Control | 15.0 | 43.7 | 72.6 | 17.3 | 51.9 | 38.6 | 98.7 | 33.7 | 12.4 | 40.3 | 42.42 |
|  | FarmCPU | 23.1 | 25.9 | 50.8 | 40.4 | 92.5 | 28.5 | 99.4 | 51.0 | 6.3 | 19.8 | 43.77 |
|  | GEMMA | 0.3 | 3.6 | 6.7 | 11.6 | 92.7 | 26.9 | 100 | 66.2 | 0 | 3.4 | 31.14 |
|  | EMMAX | 0.3 | 3.3 | 6.2 | 8.3 | 89.9 | 25.6 | 100 | 65.3 | 0 | 2.5 | 30.14 |
| Ⅳ | BLUPmrMLM | 39.6 | 61.3 | 77.3 | 66.7 | 96.8 | 59.6 | 100 | 75.3 | 33.0 | 50.4 | 66.00 |
|  | mrMLM | 20.4 | 41.4 | 87.3 | 39.7 | 84.4 | 57.8 | 99.9 | 55.7 | 18.6 | 50.8 | 55.60 |
|  | Control | 18.6 | 51.3 | 75.0 | 18.4 | 53.5 | 45.0 | 99.6 | 36.7 | 15.9 | 43.8 | 45.78 |
|  | FarmCPU | 23.5 | 27.5 | 59.5 | 46.4 | 94.6 | 32.3 | 99.5 | 53.2 | 7.1 | 22.4 | 46.60 |
|  | GEMMA | 0.4 | 3.4 | 9.3 | 11.9 | 95.0 | 30.1 | 100 | 67.7 | 0.1 | 5.8 | 32.37 |
|  | EMMAX | 0.4 | 3.4 | 8.5 | 8.2 | 92.1 | 28.7 | 100 | 67.3 | 0.1 | 4.7 | 31.34 |

*Note*: *QTN*, quantitative trait nucleotide.
