# Supplementary material for: BLUPmrMLM: A Fast mrMLM Algorithm in Genome-wide Association Studies
Source: Genomics Proteomics Bioinformatics. 2024 Feb 29;22(3):qzae020. doi: 10.1093/gpbjnl/qzae020 (PMC12016565; doi:10.1093/gpbjnl/qzae020)
Supplement: qzae020_Supplementary_Data [file qzae020_supplementary_data.zip › Table S2.docx]

**Table S2**  **Positions and effects of simulated QTNs in Monte Carlo simulation experiments**

| **QTN** | **Marker** | **Chromosome** | **Position (bp)** | **r^2^ (%)** | **QTN effect in four experiments** | | | |
| --- | --- | --- | --- | --- | --- | --- | --- | --- |
|  |  |  |  |  | **Ⅰ** | **Ⅱ** | **Ⅲ** | **Ⅳ** |
| 1 | ARS-BFGL-NGS-41048 | 1 | 9,512,145 | 3 | 1.3194 | 1.4454 | 1.4752 | 1.6160 |
| 2 | BovineHD0100003010 | 1 | 9,612,489 | 3 | 1.3558 | 1.4852 | 1.5158 | 1.6605 |
| 3 | ARS-BFGL-NGS-69447 | 2 | 10,528,990 | 5 | –1.6550 | –1.8129 | –1.8503 | –2.0269 |
| 4 | BovineHD0200005513 | 2 | 19,146,299 | 5 | 1.3613 | 1.4913 | 1.5220 | 1.6673 |
| 5 | Hapmap59741-rs29016987 | 3 | 12,824,913 | 7 | –1.7158 | –1.8796 | –1.9183 | –2.1014 |
| 6 | BovineHD0300006403 | 3 | 20,192,820 | 2 | –0.9405 | –1.0303 | –1.0515 | –1.1519 |
| 7 | BovineHD0400006437 | 4 | 21,659,730 | 10 | 2.4383 | 2.6711 | 2.7261 | 2.9863 |
| 8 | BTB-01513833 | 4 | 33,607,603 | 7 | 1.7178 | 1.8818 | 1.9206 | 2.1039 |
| 9 | BovineHD0500007593 | 5 | 25,998,740 | 4 | –1.2654 | –1.3862 | –1.4148 | –1.5498 |
| 10 | BovineHD0500007627 | 5 | 26,104,010 | 4 | 1.3558 | 1.4852 | 1.5158 | 1.6605 |

*Note*: *QTN*, quantitative trait nucleotide.
