# Supplementary material for: BLUPmrMLM: A Fast mrMLM Algorithm in Genome-wide Association Studies
Source: Genomics Proteomics Bioinformatics. 2024 Feb 29;22(3):qzae020. doi: 10.1093/gpbjnl/qzae020 (PMC12016565; doi:10.1093/gpbjnl/qzae020)
Supplement: qzae020_Supplementary_Data [file qzae020_supplementary_data.zip › Table S13.docx]

**Table S13 The AIC, BIC, and negative log-likelihood function values in the regression of trait phenotypes on all the significantly associated QTNs identified by the new and existing methods in 1439 rice hybrids**

| **Trait** | **Indicator** | **BLUPmrMLM** | **mrMLM** | **FarmCPU** | **GEMMA** | **EMMAX** |
| --- | --- | --- | --- | --- | --- | --- |
| HD | BIC | **8822.07** | 9039.93 | 9364.81 | 15,490.01 | 10,757.37 |
|  | AIC | **8437.23** | 8676.18 | 9180.3 | 11,230.48 | 10,008.79 |
|  | –Log-likelihood | **4145.62** | 4269.09 | 4555.15 | 4807.24 | 4862.40 |
| GL | BIC | 62.52 | **37.40** | 505.74 | 12,607.97 | 12,009.35 |
|  | AIC | –269.60 | –**289.45** | 199.99 | 3972.92 | 3848.75 |
|  | –Log-likelihood | –197.80 | –**206.73** | 41.99 | 348.46 | 376.38 |
| Yield | BIC | **10,603.36** | 10,674.02 | 10,838.21 | 10,857.51 | 10,889.19 |
|  | AIC | **10,392.49** | 10,526.41 | 10,785.49 | 10,804.79 | 10,847.02 |
|  | –Log-likelihood | **5156.24** | 5235.21 | 5382.75 | 5392.4 | 5415.51 |
| GN | BIC | **12,837.8** | 12,871.85 | 13,152.87 | 16,508.52 | 16,508.08 |
|  | AIC | **12,590.03** | 12,629.35 | 12,999.99 | 14,326.03 | 14,336.14 |
|  | –Log-likelihood | **6248.02** | 6268.68 | 6471.00 | 6749.02 | 6756.07 |
| TGW | BIC | **5153.85** | 5176.19 | 5838.21 | 12,249.66 | 12,023.32 |
|  | AIC | **4742.66** | 4812.44 | 5590.44 | 8121.91 | 8069.55 |
|  | –Log-likelihood | **2293.33** | 2337.22 | 2748.22 | 3277.96 | 3284.77 |

*Note*: The values with bold type are the lowest AIC, BIC, and negative log-likelihood values across the five GWAS methods. AIC, Akaike’s information criterion; BIC, Bayesian information criterion.
