# Supplementary material for: BLUPmrMLM: A Fast mrMLM Algorithm in Genome-wide Association Studies
Source: Genomics Proteomics Bioinformatics. 2024 Feb 29;22(3):qzae020. doi: 10.1093/gpbjnl/qzae020 (PMC12016565; doi:10.1093/gpbjnl/qzae020)
Supplement: qzae020_Supplementary_Data [file qzae020_supplementary_data.zip › Table S15.docx]

**Table S15 The numbers of QTNs (denominator) and their previous reported genes (numerator) for the two traits in 3K rice dataset detected by the new and existing methods**

| **Trait** | **Numbers of QTNs and their known genes** | | | | |
| --- | --- | --- | --- | --- | --- |
|  | **BLUPmrMLM** | **mrMLM** | **FarmCPU** | **GEMMA** | **EMMAX** |
| Grain length width ratio | **24**/48 | **21**/71 | **10**/26 | **8**/418 | **9**/374 |
| Thousand grain weight | **29**/66 | **21**/52 | **8**/18 | **7**/33 | **9**/38 |
