# Supplementary material for: BLUPmrMLM: A Fast mrMLM Algorithm in Genome-wide Association Studies
Source: Genomics Proteomics Bioinformatics. 2024 Feb 29;22(3):qzae020. doi: 10.1093/gpbjnl/qzae020 (PMC12016565; doi:10.1093/gpbjnl/qzae020)
Supplement: qzae020_Supplementary_Data [file qzae020_supplementary_data.zip › Table S16.docx]

**Table S16 The AIC, BIC, and negative log-likelihood function values in the regression of trait phenotypes of interest on all the significant QTNs identified by the new and existing methods in 3K rice dataset**

| **Trait** | **Indicator** | **BLUPmrMLM** | **mrMLM** | **FarmCPU** | **GEMMA** | **EMMAX** |
| --- | --- | --- | --- | --- | --- | --- |
| Grain length width ratio | BIC | 968.55 | **748.25** | 1228.58 | 3833.24 | 3572.15 |
|  | AIC | 676.97 | **327.70** | 1060.36 | 1466.93 | 1452.56 |
|  | −Log-likelihood | 286.48 | **88.85** | 500.18 | 311.46 | 348.28 |
| Thousand grain weight | BIC | **6283.50** | 6355.45 | 6841.64 | 7295.21 | 7404.48 |
|  | AIC | **5920.63** | 6065.15 | 6727.59 | 7103.41 | 7186.75 |
|  | −Log-likelihood | **2890.31** | 2976.58 | 3341.80 | 3514.70 | 3551.38 |

*Note*: The values with bold type indicate the lowest AIC, BIC, and negative log-likelihood values across five GWAS. AIC, Akaike’s information criterion; BIC, Bayesian information criterion; QTN, quantitative trait nucleotide.
