# Supplementary material for: BLUPmrMLM: A Fast mrMLM Algorithm in Genome-wide Association Studies
Source: Genomics Proteomics Bioinformatics. 2024 Feb 29;22(3):qzae020. doi: 10.1093/gpbjnl/qzae020 (PMC12016565; doi:10.1093/gpbjnl/qzae020)
Supplement: qzae020_Supplementary_Data [file qzae020_supplementary_data.zip › Table S14.docx]

**Table S14** **Previously reported genes around significant QTNs for the five traits in 1439 rice hybrids using the new and existing methods**

| **Trait** | **Gene** | **RAP locus** | **Marker** | **Chr** | **Position** | **MAF** | **BLUPmrMLM** | | | **mrMLM** | | | **FarmCPU** | **GEMMA** | **EMMAX** | **Distance**  **(kb)** | ***P* value of haplotype test** | **Ref.** | **ATAC-seq** |
| --- | --- | --- | --- | --- | --- | --- | --- | --- | --- | --- | --- | --- | --- | --- | --- | --- | --- | --- | --- |
|  |  |  |  |  |  |  | **LOD** | **Effect** | **R2(%)** | **LOD** | **Effect** | **R2(%)** | ***P* value** | ***P* value** | ***P* value** |  |  |  |  |
| **HD** | *TUT1* | Os01g0208600 | 18132 | 1 | 6011781 | 0.2019 |  |  |  |  |  |  |  | 1.40E-07 | 2.92E-07 | 114.577 | 2.26E-13 | Bai et al. (2015) | ✓ |
|  | *PME42* | Os01g0312500 | 31095 | 1 | 11829090 | 0.0549 | 6.6608 | 2.3518 | 0.3842 |  |  |  | 6.39E-08 |  |  | 54.946 | 4.02E-65 | Wang et al. (2022) | ✓ |
|  |  |  | 32213 | 1 | 12009205 | 0.065 |  |  |  | 12.2236 | 3.2178 | 0.1499 |  | 1.13E-07 | 6.35E-08 | 115.86 |  |  |  |
|  | *OsEF3* | Os01g0566100 | 75218 | 1 | 23281103 | 0.0629 | 7.4722 | -2.2958 | 0.4803 | 5.9233 | -2.4097 | 0.2187 |  |  |  | 12.773 | 6.87E-10 | Fu et al. (2009) | ✓ |
|  | *OsFTIP9* | Os01g0587300 | 81231 | 1 | 24522697 | 0.2703 | 8.2517 | -1.9365 | 0.6883 |  |  |  |  |  |  | 2.695 | 5.28E-59 | Zhang et al. (2022b) | ✓ |
|  | *DHD4* | Os02g0110100 | 137546 | 2 | 333231 | 0.3287 | 6.0233 | -1.2314 | 0.3216 |  |  |  |  |  |  | 215.183 | 9.72E-09 | Cai et al. (2021) | ✓ |
|  | *YL1* | Os02g0152900 | 145271 | 2 | 3137790 | 0.253 |  |  |  | 4.3484 | 1.2728 | 0.1147 |  |  |  | 212.719 | 4.25E-28 | Chen et al. (2016a) | ✓ |
|  | *OsUbDKγ4* | Os02g0290500 | 170404 | 2 | 10914553 | 0.1233 | 5.4051 | -1.3562 | 0.2489 |  |  |  |  |  |  | 108.647 | 5.45E-18 | Song et al. (2017) | ✓ |
|  | *OsPRR1* | Os02g0618200 | 210382 | 2 | 25484107 | 0.0486 | 8.2085 | 2.7582 | 0.4791 |  |  |  |  |  |  | 53.798 | 1.33E-29 | Zhao et al. (2011) | ✓ |
|  | *HDR1* | Os02g0793900 | 241057 | 2 | 34629843 | 0.1063 | 7.102 | 1.614 | 0.2998 |  |  |  |  |  |  | 5.653 | 4.62E-06 | Sun et al. (2016) | ✓ |
|  | *OsSOC1* | Os03g0122600 | 247431 | 3 | 942257 | 0.1782 |  |  |  | 34.0636 | -3.7846 | 1.2097 |  |  |  | 306.41 | 7.30E-57 | Tadege et al. (2003) |  |
|  |  |  | 247631 | 3 | 998982 | 0.1553 | 36.9168 | -4.2432 | 3.1965 |  |  |  |  | 4.32E-07 | 5.62E-07 | 249.685 |  |  |  |
|  |  |  | 247716 | 3 | 1027231 | 0.1591 |  |  |  |  |  |  | 1.18E-37 |  |  | 221.436 |  |  |  |
|  | *OsDof12* | Os03g0169600 | 254643 | 3 | 3841064 | 0.0407 | 3.5391 | -1.8314 | 0.1702 |  |  |  |  |  |  | 121.016 | 3.15E-13 | Li et al. (2009) | ✓ |
|  | *OsMADS1* | Os03g0215400 | 262938 | 3 | 6182756 | 0.0309 |  |  |  |  |  |  |  | 7.48E-09 | 1.01E-08 | 94.055 | 6.17E-18 | Lee et al. (2004) | ✓ |
|  | *OsWDR5* | Os03g0725400 | 325539 | 3 | 30205687 | 0.3909 | 5.6986 | 0.9995 | 0.3207 |  |  |  |  |  |  | 42.158 | 2.08E-18 | Jiang et al. (2018b) | ✓ |
|  | *SPIN1* | Os03g0815700 | 338979 | 3 | 35130799 | 0.1835 |  |  |  | 4.7904 | -1.4113 | 0.1204 |  |  |  | 89.293 | 1.41E-20 | Vega-Sánchez et al. (2008) | ✓ |
|  | *ETR2* | Os04g0169100 | 357377 | 4 | 4865716 | 0.4357 |  |  |  | 6.1624 | 0.9263 | 0.4722 |  |  |  | 124.019 | 2.51E-43 | Wuriyanghan et al. (2009) | ✓ |
|  |  |  | 358050 | 4 | 5038472 | 0.483 | 4.1431 | 0.7105 | 0.2337 |  |  |  |  |  |  | 296.775 |  |  |  |
|  | *OsRR1* | Os04g0442300 | 408715 | 4 | 21957708 | 0.1122 | 8.5485 | 2.1084 | 0.5431 | 8.2258 | 2.7389 | 0.1221 |  |  |  | 88.345 | 8.15E-28 | Cho et al. (2016) | ✓ |
|  | *OsBBX14* | Os05g0204600 | 458452 | 5 | 6533500 | 0.2596 |  |  |  |  |  |  | 3.59E-07 |  |  | 34.161 | 6.44E-34 | Bai et al. (2016) | ✓ |
|  | *OsWRKY53* | Os05g0343400 | 478404 | 5 | 15940749 | 0.1039 |  |  |  |  |  |  |  | 4.12E-07 | 6.40E-07 | 187.327 | 4.66E-20 | Hu et al. (2015b) | ✓ |
|  | *OsPUP7* | Os05g0556800 | 516251 | 5 | 27506291 | 0.2901 | 8.287 | 1.5355 | 0.5541 | 7.6663 | 1.3391 | 0.2553 |  |  |  | 265.295 | 2.48E-02 | Qi & Xiong. (2013) | ✓ |
|  | *NL1* | Os05g0578900 | 520876 | 5 | 28981204 | 0.4079 | 8.6553 | -1.3743 | 0.521 | 4.6772 | -1.4515 | 0.6465 |  |  |  | 80.753 | 1.52E-38 | Wang et al. (2009) | ✓ |
|  | *Hd17* | Os06g0142600 | 531534 | 6 | 2347746 | 0.4388 |  |  |  | 3.8566 | 0.9679 | 0.21 |  |  |  | 109.605 | 6.70E-05 | Matsubara et al. (2012) | ✓ |
|  | *Hd3a* | Os06g0157700 | 533972 | 6 | 3070510 | 0.3183 | 11.8788 | 2.7237 | 1.2455 | 4.4734 | 1.8366 | 0.1091 |  |  |  | 129.057 | 2.08E-07 | Takahashi et al. (2009) | ✓ |
|  |  |  | 534573 | 6 | 3272231 | 0.0813 |  |  |  |  |  |  | 1.55E-11 |  |  | 330.778 |  |  |  |
|  | *AID1* | Os06g0181300 | 537829 | 6 | 4287800 | 0.0705 |  |  |  | 5.5533 | -2.2082 | 0.4284 |  |  |  | 269.483 | 1.61E-31 | Zhu et al. (2004) | ✓ |
|  | *OsNF-YB9* | Os06g0285200 | 555876 | 6 | 9831596 | 0.2192 | 4.0281 | 0.7829 | 0.1256 | 5.5722 | 1.1825 | 0.1181 |  |  |  | 305.228 | 6.39E-20 | Das et al. (2019) | ✓ |
|  | *SE5* | Os06g0603000 | 583214 | 6 | 24658661 | 0.2856 | 5.0886 | 1.2288 | 0.282 |  |  |  |  |  |  | 72.056 | 2.50E-05 | Andrés et al. (2009) | ✓ |
|  | *pls3* | Os07g0247100 | 627369 | 7 | 8058344 | 0.0441 |  |  |  |  |  |  |  | 3.86E-07 | 6.08E-07 | 167.836 | 1.06E-09 | Hong et al. (2018) | ✓ |
|  | *Ghd7* | Os07g0261200 | 633820 | 7 | 9164703 | 0.1369 | 21.9234 | -2.4042 | 1.3049 | 8.2748 | -1.6694 | 0.4938 |  |  |  | 19.856 | 2.05E-43 | Xue et al. (2008) |  |
|  |  |  | 633821 | 7 | 9164732 | 0.1383 |  |  |  |  |  |  | 3.43E-13 |  |  | 19.827 |  |  |  |
|  | *OsMADS18* | Os07g0605200 | 689949 | 7 | 25251835 | 0.0347 | 5.6321 | 4.0206 | 0.7286 |  |  |  | 1.07E-13 | 3.34E-07 | 4.83E-07 | 196.798 | 2.31E-73 | Fornara et al. (2004) | ✓ |
|  | *OsLHY* | Os08g0157600 | 714889 | 8 | 3368875 | 0.0417 |  |  |  |  |  |  |  | 3.03E-07 |  | 3.534 | 1.55E-90 | Sun et al. (2021) |  |
|  |  |  | 715446 | 8 | 3515772 | 0.4573 | 7.5436 | 1.2279 | 0.4724 |  |  |  |  |  |  | 143.363 |  |  |  |
|  | *Ghd8* | Os08g0174500 | 718291 | 8 | 4306691 | 0.0546 |  |  |  |  |  |  | 9.93E-61 |  |  | 26.037 | 8.72E-10 | Dai et al. (2012) | ✓ |
|  |  |  | 718316 | 8 | 4309956 | 0.0445 | 33.424 | 8.4168 | 4.0172 | 29.3726 | 8.6568 | 0.5441 |  | 1.60E-11 | 1.93E-11 | 22.772 |  |  |  |
|  | *OsVIL1* | Os08g0220600 | 725605 | 8 | 7143708 | 0.3141 |  |  |  | 9.9412 | -4.3141 | 0.4697 |  |  |  | 194.27 | 1.35E-117 | Jeong et al. (2016) | ✓ |
|  |  |  | 726433 | 8 | 7467630 | 0.0372 |  |  |  |  |  |  |  | 1.62E-07 | 4.22E-07 | 120.52 |  |  |  |
|  | *OsNTL5* | Os08g0562200 | 772506 | 8 | 28200881 | 0.2457 |  |  |  | 4.3126 | 1.5018 | 0.3174 |  |  |  | 32.000 | 2.98E-36 | Guo et al. (2018a) | ✓ |
|  | *FCA* | Os09g0123200 | 780022 | 9 | 1611940 | 0.2936 |  |  |  |  |  |  |  | 2.10E-07 |  | 125.218 | 6.32E-06 | Lee et al. (2005) | ✓ |
|  | *OsCO3* | Os09g0240200 | 784668 | 9 | 3445859 | 0.2912 |  |  |  |  |  |  |  | 2.06E-08 | 1.32E-07 | 167.934 | 3.36E-08 | Kim et al. (2008) | ✓ |
|  | *OsRRM* | Os09g0298700 | 802726 | 9 | 7659963 | 0.3336 | 12.3691 | -2.3508 | 1.1456 |  |  |  |  |  |  | 198.762 | 8.67E-06 | Chen et al. (2007b) | ✓ |
|  |  |  | 802931 | 9 | 7697771 | 0.3412 |  |  |  | 21.0518 | -3.144 | 1.2791 |  |  |  | 160.954 |  |  |  |
|  | *OsbZIP71* | Os09g0306400 | 805038 | 9 | 8324359 | 0.3694 |  |  |  |  |  |  | 9.85E-07 |  |  | 112.942 | 3.45E-16 | Li et al. (2022b) | ✓ |
|  | *EMF2B* | Os09g0306800 | 805884 | 9 | 8527843 | 0.4521 |  |  |  |  |  |  | 6.41E-07 |  |  | 23.97 | 1.73E-2 | Xie et al. (2015) | ✓ |
|  | *OsMADS8* | Os09g0507200 | 833716 | 9 | 20079530 | 0.4034 |  |  |  | 13.1946 | -2.3217 | 1.5525 |  |  |  | 227.565 | 1.76E-34 | Kang et al. (1997) |  |
|  |  |  | 834102 | 9 | 20154141 | 0.4041 | 10.1384 | -1.8827 | 0.9275 |  |  |  |  |  |  | 152.954 |  |  | ✓ |
|  |  |  | 834298 | 9 | 20207541 | 0.2457 |  |  |  |  |  |  | 3.25E-09 |  |  | 99.554 |  |  |  |
|  | *Ugp1* | Os09g0553200 | 842705 | 9 | 22730236 | 0.2109 | 3.4492 | -0.7144 | 0.0843 |  |  |  |  |  |  | 18.73 | 1.20E-06 | Chen et al. (2007a) | ✓ |
|  | *SIP1* | Os09g0560900 | 844158 | 9 | 23268054 | 0.0643 | 6.3754 | -2.0676 | 0.3448 |  |  |  |  |  |  | 144.668 | 2.33E-14 | Jiang et al. (2018c) | ✓ |
|  | *Ehd1* | Os10g0463400 | 899612 | 10 | 17263148 | 0.45 | 7.6027 | 1.5263 | 0.4066 | 3.0208 | 1.3407 | 0.3789 |  |  |  | 270.162 | 5.31E-18 | Takahashi et al. (2009) |  |
| **GL** | *OsRA2* | Os01g0169400 | 10558 | 1 | 3543831 | 0.3808 |  |  |  | 3.3406 | 0.0885 | 0.3255 |  |  |  | 6.22 | 2.48E-02 | Lu et al. (2017) | ✓ |
|  | *OsSDG721* | Os01g0218800 | 20232 | 1 | 6588093 | 0.2432 |  |  |  |  |  |  | 5.71E-09 |  |  | 74.222 | 1.92E-09 | Liu et al. (2021b) | ✓ |
|  | *RDD1* | Os01g0264000 | 24769 | 1 | 9120959 | 0.2568 | 5.1793 | 0.0551 | 0.2389 | 7.3671 | 0.0572 | 0.1158 |  |  |  | 169.267 | 1.99E-15 | Iwamoto et al. (2009) |  |
|  | *OsVQ4* | Os01g0808900 | 118210 | 1 | 36152799 | 0.0973 |  |  |  | 3.6449 | 0.0462 | 0.0295 |  |  |  | 39.502 | 6.83E-44 | Chan et al. (2021) | ✓ |
|  |  |  | 119134 | 1 | 36335003 | 0.213 | 10.3666 | 0.0744 | 0.3648 |  |  |  |  |  |  | 221.706 |  |  |  |
|  | *OsCCS52B* | Os01g0972900 | 136276 | 1 | 45051121 | 0.1883 | 3.4522 | -0.0381 | 0.091 |  |  |  |  |  |  | 303.561 | 1.59E-36 | Su’udi et al. (2012) | ✓ |
|  | *OsNAC23* | Os02g0214500 | 154830 | 2 | 6265880 | 0.3892 |  |  |  | 8.8513 | 0.095 | 0.3746 |  |  |  | 167.139 | 8.49E-06 | Li et al. (2022c) | ✓ |
|  | *WG1* | Os02g0512400 | 188803 | 2 | 18868756 | 0.2196 |  |  |  | 3.9823 | 0.0567 | 0.0362 |  |  |  | 324.402 | 5.89E-11 | Hao et al. (2021) |  |
|  |  |  | 190345 | 2 | 19376524 | 0.2616 | 3.4581 | -0.0399 | 0.1332 |  |  |  |  |  |  | 182.343 |  |  | ✓ |
|  |  |  | 190872 | 2 | 19490752 | 0.4375 |  |  |  |  |  |  | 6.96E-07 |  |  | 296.571 |  |  |  |
|  | *LARGE1* | Os02g0517531 | 190876 | 2 | 19491548 | 0.4347 |  |  |  |  |  |  | 2.43E-07 |  |  | 57.035 | 1.27E-08 | Lyu et al. (2020) | ✓ |
|  |  |  | 191961 | 2 | 19819563 | 0.2797 | 4.9995 | -0.0539 | 0.2066 | 7.9042 | -0.0849 | 0.1647 |  |  |  | 172.059 |  |  |  |
|  | *TUD1* | Os03g0232600 | 265028 | 3 | 7315635 | 0.0445 |  |  |  |  |  |  |  | 2.07E-07 | 2.53E-07 | 234.699 | 1.16E-17 | Hu et al. (2013) | ✓ |
|  | *GS3* | Os03g0407400 | 289234 | 3 | 17304860 | 0.2029 |  |  |  | 39.4432 | -0.2845 | 3.901 |  |  |  | 60.445 | 4.01E-242 | Fan et al. (2009) |  |
|  |  |  | 289445 | 3 | 17344469 | 0.1932 | 98.3528 | -0.4297 | 15.1184 |  |  |  |  |  |  | 20.836 |  |  |  |
|  |  |  | 289602 | 3 | 17365286 | 0.1852 |  |  |  |  |  |  |  | 1.77E-34 | 3.56E-27 | 0.019 |  |  |  |
|  |  |  | 290492 | 3 | 17514095 | 0.1918 |  |  |  |  |  |  | 1.54E-147 |  |  | 143.063 |  |  |  |
|  | *GL3.2* | Os03g0417700 | 291180 | 3 | 17656931 | 0.3242 |  |  |  |  |  |  |  | 4.43E-14 | 1.59E-12 | 319.632 | 4.43E-18 | Xu et al. (2015) | ✓ |
|  | *OsRGB1* | Os03g0669200 | 315715 | 3 | 27280934 | 0.237 | 6.4857 | -0.0718 | 0.3426 |  |  |  |  |  |  | 113.984 | 1.44E-59 | Sun et al. (2018) | ✓ |
|  | *GF14f* | Os03g0710800 | 323660 | 3 | 29635378 | 0.2384 |  |  |  | 3.23 | -0.0682 | 0.1123 |  |  |  | 207.005 | 7.96E-64 | Zhang et al. (2019b) | ✓ |
|  | *OsMKB3* | Os03g0733600 | 328016 | 3 | 31074102 | 0.0817 | 4.0789 | 0.0653 | 0.1432 |  |  |  |  |  |  | 262.149 | 2.17E-08 | Shimano et al. (2018) |  |
|  | *qTGW3* | Os03g0841800 | 340902 | 3 | 36022679 | 0.2995 |  |  |  | 4.5832 | 0.0547 | 0.2372 |  |  |  | 214.008 | 7.20E-05 | Ying et al. (2018) | ✓ |
|  | *OsSRT1* | Os04g0271000 | 374430 | 4 | 11259278 | 0.0942 | 5.2248 | -0.0632 | 0.2151 |  |  |  |  |  |  | 103.771 | 3.82E-02 | Zhang et al. (2016b) | ✓ |
|  | *OsAGO2* | Os04g0615700 | 434515 | 4 | 31865929 | 0.0803 | 11.2405 | -0.1342 | 0.6868 |  |  |  |  |  |  | 223.963 | 1.65E-40 | Yin et al. (2020) | ✓ |
|  |  |  | 434684 | 4 | 31915851 | 0.0368 |  |  |  |  |  |  | 1.04E-12 |  |  | 273.885 |  |  |  |
|  | *GSN1* | Os05g0115800 | 446424 | 5 | 824902 | 0.1258 |  |  |  |  |  |  | 5.03E-10 |  |  | 11.551 | 1.50E-41 | Guo et al. (2018b) | ✓ |
|  |  |  | 446425 | 5 | 825375 | 0.1338 | 5.185 | 0.091 | 0.4545 | 9.3323 | 0.0959 | 0.1394 |  |  |  | 11.078 |  |  |  |
|  | *SRS3* | Os05g0154700 | 450214 | 5 | 3073293 | 0.3478 | 4.1108 | 0.0307 | 0.0633 |  |  |  |  |  |  | 107.795 | 2.16E-14 | Kitagawa et al. (2010) | ✓ |
|  | *OsOFP19* | Os05g0324600 | 476225 | 5 | 15254693 | 0.246 |  |  |  |  |  |  | 8.69E-07 |  |  | 206.399 | 3.08E-14 | Yang et al. (2018) | ✓ |
|  | *OsDREB1C* | Os06g0127100 | 527714 | 6 | 1160139 | 0.4802 |  |  |  | 3.6149 | -0.0594 | 0.4201 |  |  |  | 273.632 | 8.08E-12 | Wei et al. (2022) | ✓ |
|  | *miR1432* |  | 536687 | 6 | 3989471 | 0.0327 | 4.5876 | -0.1443 | 0.3203 |  |  |  |  |  |  | 319.707 | 8.80E-44 | Zhao et al. (2019) | ✓ |
|  |  |  | 536754 | 6 | 4013603 | 0.2436 |  |  |  | 11.3352 | -0.0897 | 0.4026 |  |  |  | 343.839 |  |  |  |
|  | *OsER1* | Os06g0203800 | 540926 | 6 | 5168490 | 0.4559 |  |  |  | 3.6487 | 0.0677 | 0.093 |  |  |  | 74.75 | 3.34E-36 | Guo et al. (2020) | ✓ |
|  |  |  | 542034 | 6 | 5611960 | 0.4475 | 11.5567 | 0.1009 | 0.9523 |  |  |  |  |  |  | 361.116 |  |  |  |
|  | *OsBU1* | Os06g0226500 | 546735 | 6 | 6769923 | 0.3648 |  |  |  |  |  |  | 1.98E-12 |  |  | 213.174 | 1.59E-26 | Tanaka et al. (2009) | ✓ |
|  | *OsNF-YB9* | Os06g0285200 | 555752 | 6 | 9805862 | 0.1949 | 3.1753 | 0.0461 | 0.1556 |  |  |  |  |  |  | 330.962 | 1.32E-17 | Das et al. (2019) | ✓ |
|  | *GL6* | Os06g0666100 | 597295 | 6 | 28315712 | 0.0625 |  |  |  |  |  |  | 5.18E-12 |  |  | 118.473 | 2.40E-02 | Wang et al. (2019) | ✓ |
|  |  |  | 597553 | 6 | 28453136 | 0.3301 | 3.4286 | -0.0309 | 0.0879 |  |  |  |  |  |  | 14.536 |  |  |  |
|  | *OsIQD14* | Os08g0115200 | 709688 | 8 | 825491 | 0.1397 |  |  |  | 5.6995 | 0.0617 | 0.0774 |  |  |  | 28.944 | 9.26E-04 | Yang et al. (2020) | ✓ |
|  | *DEP1* | Os09g0441900 | 824420 | 9 | 17040884 | 0.1254 | 3.6063 | 0.049 | 0.1137 |  |  |  |  |  |  | 23.978 | 3.33E-45 | Sun et al. (2018) | ✓ |
|  | *OsSCP46* | Os10g0101200 | 844754 | 10 | 107290 | 0.1619 |  |  |  | 4.4835 | -0.0598 | 0.0191 |  |  |  | 11.804 | 6.00E-07 | Li et al. (2016) | ✓ |
|  | *GL10* | Os10g0536100 | 909120 | 10 | 21259651 | 0.0851 | 3.2502 | -0.0527 | 0.0954 |  |  |  |  |  |  | 58.772 | 4.46E-06 | Zhan et al. (2022) | ✓ |
|  |  |  | 909241 | 10 | 21335815 | 0.0782 |  |  |  | 9.8181 | -0.1271 | 0 | 1.32E-13 |  |  | 6.899 |  |  |  |
|  | *NAL2* | Os11g0102100 | 916324 | 11 | 373328 | 0.4698 | 4.1024 | 0.0299 | 0.0683 |  |  |  |  |  |  | 305.373 | 3.60E-05 | Cho et al. (2013) |  |
|  | *CycT1;3* | Os11g0157100 | 924212 | 11 | 2591163 | 0.4903 | 17.8002 | -0.1263 | 1.3977 | 23.474 | -0.1589 | 3.47 |  |  |  | 122.173 | 1.56E-14 | Qi et al. (2012) | ✓ |
|  |  |  | 924495 | 11 | 2639531 | 0.4931 |  |  |  |  |  |  | 1.63E-30 |  |  | 73.805 |  |  |  |
|  | *SRS5* | Os11g0247300 | 941454 | 11 | 8263239 | 0.214 | 5.1662 | 0.0367 | 0.1377 |  |  |  |  |  |  | 297.936 | 3.54E-16 | Segami et al. (2012) | ✓ |
|  | *OsMPK15* | Os11g0271100 | 945607 | 11 | 9799764 | 0.1049 | 3.5303 | -0.0458 | 0.0836 |  |  |  |  |  |  | 275.871 | 8.45E-55 | Hong et al. (2019) | ✓ |
|  | *OsPPKL3* | Os12g0617900 | 1094962 | 12 | 26467783 | 0.0841 |  |  |  |  |  |  | 1.22E-09 |  |  | 65.427 | 9.76E-28 | Zhang et al. (2012) | ✓ |
|  |  |  | 1095873 | 12 | 26680474 | 0.3506 | 7.0329 | 0.061 | 0.354 |  |  |  |  |  |  | 138.224 |  |  |  |
| **Yield** | *SPL33* | Os01g0116600 | 3187 | 1 | 769841 | 0.443 | 3.1758 | -1.537 | 0.4613 | 4.0222 | -2.3982 | 1.2062 |  |  |  | 159.043 | 3.47E-02 | Wang et al. (2017) | ✓ |
|  | *ESA1* | Os01g0524100 | 65643 | 1 | 20445931 | 0.2182 | 4.7354 | -1.7199 | 0.7065 | 5.0197 | -2.3033 | 0.3892 |  |  |  | 67.224 | 3.77E-02 | Hou et al. (2019) | ✓ |
|  | *OsERF3* | Os01g0797600 | 115420 | 1 | 35534760 | 0.499 |  |  |  | 3.3659 | -20.1121 | 0 |  |  |  | 10.83 | 4.08E-04 | Ramegowda et al. (2014) | ✓ |
|  | *OsSPL4* | Os02g0174100 | 148587 | 2 | 4376155 | 0.04 |  |  |  |  |  |  |  | 9.12E-07 |  | 298.209 | 9.27E-03 | Hu et al. (2020) | ✓ |
|  | *OsSta2* | Os02g0655200 | 217067 | 2 | 27067079 | 0.286 | 4.8707 | -2.0171 | 0.9217 |  |  |  |  |  |  | 276.232 | 1.27E-04 | Kumar et al. (2017) | ✓ |
|  | *OsDHHC06* | Os02g0819100 | 244606 | 2 | 36319244 | 0.4983 | 3.1445 | -4.4072 | 0.3202 |  |  |  |  |  |  | 273.661 | 4.11E-03 | Zhou et al. (2017a) | ✓ |
|  | *RLS3* | Os03g0586900 | 302803 | 3 | 22276018 | 0.0469 | 3.0036 | 2.5681 | 0.5564 | 4.8554 | 3.5885 | 0.3774 |  | 1.59E-07 | 1.56E-07 | 157.844 | 6.83E-09 | Lin et al. (2016) | ✓ |
|  | *TT3.1* | Os03g0706900 | 321782 | 3 | 29039507 | 0.1828 | 4.0044 | -1.3465 | 0.3524 |  |  |  |  |  |  | 169.749 | 2.75E-02 | Zhang et al. (2022a) | ✓ |
|  | *FLO19* | Os04g0119400 | 346496 | 4 | 923360 | 0.1977 |  |  |  | 3.4681 | -1.5832 | 0.4105 |  |  |  | 199.659 | 2.59E-09 | Lei et al. (2022) | ✓ |
|  | *YGL1* | Os05g0349700 | 480915 | 5 | 16682439 | 0.2943 | 7.1554 | 2.1208 | 1.3979 |  |  |  |  |  |  | 186.778 | 2.37E-02 | Wu et al. (2007) | ✓ |
|  | *ALK* | Os06g0229800 | 546657 | 6 | 6751358 | 0.3687 |  |  |  | 4.2461 | -1.8388 | 1.2402 |  |  |  | 0.979 | 7.96E-04 | Huang et al. (2015) | ✓ |
|  |  |  | 546749 | 6 | 6782647 | 0.442 |  |  |  |  |  |  | 5.39E-07 |  |  | 30.31 |  |  |  |
|  |  |  | 547099 | 6 | 6858284 | 0.3965 | 9.2133 | -2.2773 | 1.9357 |  |  |  |  |  |  | 105.947 |  |  |  |
|  | *OsMTS1* | Os07g0247100 | 628143 | 7 | 8182005 | 0.214 | 3.1267 | 1.2743 | 0.3291 |  |  |  |  |  |  | 44.175 | 6.34E-04 | Hong et al. (2018) | ✓ |
|  | *OsIQD14* | Os08g0115200 | 708829 | 8 | 529864 | 0.4659 |  |  |  |  |  |  | 2.89E-08 |  |  | 264.591 | 1.99E-02 | Yang et al. (2020) | ✓ |
|  | *Ghd8* | Os08g0174500 | 718808 | 8 | 4392481 | 0.0424 |  |  |  | 4.2905 | 3.7609 | 0 |  | 3.72E-07 | 3.82E-07 | 58.114 | 9.98E-03 | Huang et al. (2015) | ✓ |
|  | *ARE1* | Os08g0224300 | 726803 | 8 | 7609915 | 0.4097 |  |  |  | 5.5079 | -1.9863 | 1.3319 |  |  |  | 36.961 | 1.37E-03 | Wang et al. (2018) | ✓ |
|  |  |  | 726996 | 8 | 7678708 | 0.0956 | 3.0617 | -1.6127 | 0.329 |  |  |  |  |  |  | 105.754 |  |  |  |
|  | *OsSPL18* | Os09g0507100 | 834705 | 9 | 20305177 | 0.2269 |  |  |  |  |  |  | 4.56E-09 |  |  | 3.231 | 2.19E-04 | Yuan et al. (2019) | ✓ |
| **GN** | *Gn1a* | Os01g0197600 | 13810 | 1 | 4996413 | 0.0396 |  |  |  |  |  |  | 4.26E-14 |  |  | 272.69 | 1.64E-04 | Ashikari et al., 2005) |  |
|  |  |  | 14653 | 1 | 5212107 | 0.418 | 8.4647 | 7.2748 | 2.1725 | 11.5144 | 9.5649 | 3.8228 |  |  |  | 56.996 |  |  |  |
|  | *SDG721* | Os01g0218800 | 20178 | 1 | 6576432 | 0.222 | 7.3241 | -7.3088 | 2.0646 | 7.8571 | -10.2432 | 1.2106 |  |  |  | 62.561 | 2.89E-07 | Jiang et al. (2018a) | ✓ |
|  | *OsSRLK* | Os01g0223600 | 20734 | 1 | 6697922 | 0.287 |  |  |  | 4.9825 | 4.4167 | 0.492 |  |  |  | 67.569 | 7.07E-17 | Shin et al. (2019) | ✓ |
|  |  |  | 20833 | 1 | 6723543 | 0.2477 | 4.2422 | 4.5818 | 0.9516 |  |  |  |  |  |  | 41.948 |  |  |  |
|  | *OsFBK1* | Os01g0659900 | 91547 | 1 | 28437135 | 0.1049 |  |  |  | 4.3579 | 7.5561 | 0.021 |  |  |  | 190.928 | 7.57E-04 | Borna et al. (2022) | ✓ |
|  | *RGN1* | Os01g0685400 | 97389 | 1 | 30023887 | 0.2564 | 3.7376 | -3.1251 | 0.3399 | 3.1105 | -4.0053 | 0.0529 |  |  |  | 16.531 | 5.51E-07 | Li et al. (2022a) | ✓ |
|  | *OsMTA2* | Os02g0672600 | 222378 | 2 | 28347530 | 0.1925 | 13.3262 | 6.9363 | 2.2031 |  |  |  |  |  |  | 94.877 | 3.22E-09 | Zhang et al. (2019a) | ✓ |
|  | *OsGRF4* | Os02g0701300 | 227289 | 2 | 29545297 | 0.1546 |  |  |  | 4.7808 | 5.9269 | 0.2176 |  |  |  | 204.838 | 3.20E-04 | Hu et al. (2015a) | ✓ |
|  | *Ghd2* | Os02g0731700 | 235817 | 2 | 31569380 | 0.0483 |  |  |  |  |  |  | 2.64E-07 |  |  | 206.719 | 1.23E-06 | Liu et al. (2016) | ✓ |
|  | *LF1* | Os03g0109400 | 246569 | 3 | 450122 | 0.3777 | 5.3571 | -5.3114 | 1.9659 |  |  |  |  |  |  | 78.048 | 1.18E-17 | Zhang et al. (2017) | ✓ |
|  | *OscpSRP43* | Os03g0131900 | 250468 | 3 | 1807982 | 0.2189 |  |  |  |  |  |  | 1.39E-14 |  |  | 10.813 | 1.35E-02 | Lv et al. (2015) | ✓ |
|  |  |  | 250840 | 3 | 1904524 | 0.2199 |  |  |  | 3.3094 | 5.4825 | 0.8962 |  |  |  | 107.355 |  |  |  |
|  | *OsLBD37* | Os03g0445700 | 295410 | 3 | 19308600 | 0.4851 | 6.7299 | 4.8248 | 1.6905 |  |  |  |  |  |  | 262.532 | 3.97E-07 | Li et al. (2017) | ✓ |
|  | *OsFdC2* | Os03g0685000 | 318292 | 3 | 28149319 | 0.1956 | 3.5295 | -3.6585 | 0.4352 |  |  |  |  |  |  | 75.988 | 1.53E-03 | Li et al. (2015a) | ✓ |
|  | *OsTB1* | Os03g0706500 | 322504 | 3 | 29191166 | 0.0386 |  |  |  |  |  |  |  | 9.14E-09 | 2.09E-08 | 0.277 | 2.08E-07 | Yano et al. (2015) | ✓ |
|  |  |  | 322585 | 3 | 29206767 | 0.0389 | 15.7916 | 17.7497 | 3.0578 |  |  |  |  |  |  | 15.878 |  |  |  |
|  |  |  | 322712 | 3 | 29249292 | 0.0389 |  |  |  |  |  |  | 3.35E-15 |  |  | 58.403 |  |  |  |
|  |  |  | 323178 | 3 | 29425314 | 0.2488 |  |  |  | 3.9125 | 4.7901 | 0.321 |  |  |  | 234.425 |  |  |  |
|  | *ETR2* | Os04g0169100 | 357902 | 4 | 5001373 | 0.1574 | 3.5783 | -3.0133 | 0.3017 |  |  |  |  |  |  | 259.676 | 1.40E-06 | Wuriyanghan et al. (2009) | ✓ |
|  | *OsAP2-39* | Os04g0610400 | 432469 | 4 | 31289837 | 0.4885 | 3.6219 | -10.7806 | 0.3455 |  |  |  |  |  |  | 47.982 | 4.96E-04 | Yaish et al. (2010) | ✓ |
|  |  |  | 433223 | 4 | 31577674 | 0.3127 |  |  |  |  |  |  | 5.56E-10 |  |  | 239.187 |  |  |  |
|  | *SRT1* | Os04g0663600 | 439372 | 4 | 34199996 | 0.2783 |  |  |  | 3.9454 | -5.0348 | 0.3014 |  |  |  | 58.915 | 7.72E-06 | Mjomba et al. (2016) | ✓ |
|  | *CYP734A4* | Os06g0600400 | 584029 | 6 | 24842096 | 0.1223 | 4.8641 | -4.1576 | 0.4333 |  |  |  |  |  |  | 257.767 | 9.67E-03 | Qian et al. (2017) | ✓ |
|  | *sdt* | Os06g0649600 | 593378 | 6 | 27429302 | 0.2533 |  |  |  |  |  |  | 1.07E-08 |  |  | 2.238 | 8.14E-15 | Zhao et al. (2015) | ✓ |
|  | *Ghd7* | Os07g0261200 | 633402 | 7 | 9080855 | 0.4802 | 4.4326 | -3.4585 | 0.4683 |  |  |  |  |  |  | 103.704 | 1.40E-16 | Xue et al. (2008) |  |
|  | *OsPHR2* | Os07g0438800 | 655577 | 7 | 15454898 | 0.1869 |  |  |  |  |  |  | 4.20E-08 |  |  | 35.367 | 4.30E-28 | Guo et al. (2015) | ✓ |
|  | *FON4* | Os11g0595400 | 1001593 | 11 | 24845991 | 0.2637 | 4.9627 | 4.0972 | 0.8243 |  |  |  |  |  |  | 338.111 | 8.32E-04 | Xu et al. (2017) | ✓ |
|  | *OsCD1* | Os12g0555600 | 1083625 | 12 | 22876322 | 0.46 |  |  |  | 11.1846 | 7.7996 | 5.2477 |  |  |  | 65.321 | 2.53E-18 | Luan et al. (2011) | ✓ |
|  |  |  | 1084248 | 12 | 23067040 | 0.4531 | 3.9691 | -4.0196 | 1.0074 |  |  |  |  |  |  | 256.039 |  |  |  |
| **TGW** | *OsBZR2* | Os01g0203000 | 8727 | 1 | 2643977 | 0.2026 |  |  |  | 5.4472 | -0.3565 | 0.2633 |  |  |  | 249.775 | 9.13E-51 | Liu et al. (2021a) | ✓ |
|  | *OsRA2* | Os01g0169400 | 10403 | 1 | 3498549 | 0.1939 |  |  |  |  |  |  | 2.20E-11 |  |  | 51.502 | 1.63E-03 | Lu et al. (2017) | ✓ |
|  |  |  | 10542 | 1 | 3539763 | 0.3895 | 5.6998 | 0.3991 | 0.4664 |  |  |  |  |  |  | 10.288 |  |  |  |
|  | *OsMADS51* | Os01g0922800 | 128012 | 1 | 42297914 | 0.0876 | 4.4609 | 0.3462 | 0.192 |  |  |  |  |  |  | 138.705 | 7.09E-03 | Wei et al. (2021) | ✓ |
|  | *OsWAK11* | Os02g0111600 | 139244 | 2 | 856455 | 0.4458 |  |  |  | 10.0501 | 0.5284 | 1.0743 |  |  |  | 225.891 | 8.01E-47 | Yue et al. (2022) | ✓ |
|  | *OsMADS29* | Os02g0170300 | 147299 | 2 | 4040874 | 0.2675 | 6.5294 | -0.3933 | 0.5771 |  |  |  |  |  |  | 203.742 | 7.94E-06 | Yang et al. (2012) |  |
|  |  |  | 147371 | 2 | 4061580 | 0.2272 |  |  |  | 6.5268 | -0.5205 | 0.4542 |  |  |  | 224.448 |  |  |  |
|  | *OsNAC23* | Os02g0214500 | 154576 | 2 | 6162718 | 0.4399 | 3.5272 | -0.1558 | 0.0892 |  |  |  |  |  |  | 270.301 | 1.33E-12 | Li et al. (2022c) | ✓ |
|  | *GW2* | Os02g0244100 | 158808 | 2 | 8206065 | 0.3374 |  |  |  | 8.6845 | 0.546 | 1.0844 |  |  |  | 55.415 | 3.87E-30 | Hao et al. (2021) | ✓ |
|  |  |  | 160155 | 2 | 8511689 | 0.2985 | 7.4374 | 0.3736 | 0.7835 |  |  |  |  |  |  | 361.039 |  |  |  |
|  | *LARGE1* | Os02g0517531 | 190317 | 2 | 19370871 | 0.0869 | 7.3749 | 0.6174 | 0.5849 |  |  |  |  |  |  | 177.712 | 7.32E-36 | Lyu et al. (2020) | ✓ |
|  | *OsNF-YB1* | Os02g0725900 | 233174 | 2 | 30858437 | 0.1101 |  |  |  | 4.2278 | 0.4117 | 0.0393 |  |  |  | 219.909 | 2.97E-03 | Xu et al. (2016) | ✓ |
|  | *OsSOC1* | Os03g0122600 | 248378 | 3 | 1222701 | 0.1438 |  |  |  |  |  |  |  | 4.95E-07 | 7.44E-07 | 25.966 | 3.16E-10 | Huang et al. (2015) |  |
|  | *flo10* | Os03g0168400 | 253726 | 3 | 3470943 | 0.4576 | 3.4665 | 0.3533 | 0.1052 |  |  |  |  |  |  | 197.398 | 1.08E-02 | Wu et al. (2019) |  |
|  | *GS3* | Os03g0407400 | 289602 | 3 | 17365286 | 0.1852 |  |  |  |  |  |  |  | 5.26E-15 | 6.52E-14 | 0.019 | 1.83E-29 | Fan et al. (2006) |  |
|  |  |  | 289621 | 3 | 17369402 | 0.1939 |  |  |  | 40.6271 | -1.5133 | 4.686 | 2.85E-27 |  |  | 1.63 |  |  |  |
|  |  |  | 291163 | 3 | 17650070 | 0.3231 | 27.0866 | -0.9066 | 4.343 |  |  |  |  |  |  | 279.038 |  |  |  |
|  | *OsNF-YA4* | Os03g0696300 | 319777 | 3 | 28506535 | 0.1265 | 3.1685 | 0.2094 | 0.0869 |  |  |  |  |  |  | 144.255 | 1.41E-39 | Lee et al. (2015) | ✓ |
|  | *GF14f* | Os03g0710800 | 322905 | 3 | 29318978 | 0.2616 | 4.744 | -0.3213 | 0.4718 |  |  |  |  |  |  | 105.46 | 1.98E-42 | Zhang et al. (2019b) | ✓ |
|  | *qTGW3* | Os03g0841800 | 340902 | 3 | 36022679 | 0.2995 | 10.6757 | 0.4606 | 0.9363 |  |  |  |  |  |  | 214.008 | 1.82E-12 | Ying et al. (2018) | ✓ |
|  |  |  | 342092 | 3 | 36336022 | 0.3054 |  |  |  | 3.9542 | 0.6804 | 1.1269 |  |  |  | 93.308 |  |  |  |
|  | *ETR2* | Os04g0169100 | 356144 | 4 | 4585084 | 0.3728 | 4.2643 | 0.1953 | 0.152 |  |  |  |  |  |  | 152.64 | 1.39E-45 | Wuriyanghan et al. (2009) | ✓ |
|  | *OscZOG1* | Os04g0271700 | 374961 | 4 | 11353358 | 0.246 | 3.1142 | 0.1879 | 0.0941 |  |  |  |  |  |  | 68.946 | 6.92E-05 | Shang et al. (2016) | ✓ |
|  | *D11* | Os04g0469800 | 411820 | 4 | 23597077 | 0.18 |  |  |  |  |  |  | 5.64E-10 |  |  | 268.63 | 5.61E-25 | Zhu et al. (2015) | ✓ |
|  | *sgsd3* | Os04g0545000 | 418947 | 4 | 27571080 | 0.0479 | 4.0574 | -0.561 | 0.3045 |  |  |  |  |  |  | 111.74 | 1.79E-30 | Lan et al. (2020) | ✓ |
|  | *GSD1* | Os04g0620200 | 434493 | 4 | 31859404 | 0.2929 | 3.6186 | -0.2798 | 0.3362 |  |  |  |  |  |  | 63.757 | 2.22E-51 | Gui et al. (2014) | ✓ |
|  | *OsJMT1* | Os05g0102000 | 445036 | 5 | 312739 | 0.1119 | 6.9363 | 0.4439 | 0.4192 |  |  |  |  |  |  | 216.429 | 6.75E-16 | Qi et al. (2016) | ✓ |
|  |  |  | 445191 | 5 | 352810 | 0.065 |  |  |  |  |  |  | 1.37E-14 |  |  | 256.5 |  |  |  |
|  | *RSR1* | Os05g0121600 | 447246 | 5 | 1170678 | 0.1122 |  |  |  | 3.8584 | -0.4335 | 0.0055 |  |  |  | 32.377 | 2.02E-05 | Fu & Xue. (2010) | ✓ |
|  | *OsDER1* | Os05g0187800 | 453735 | 5 | 5281106 | 0.222 | 4.4084 | 0.3166 | 0.3325 |  |  |  |  |  |  | 95.285 | 2.50E-02 | Qian et al. (2018) | ✓ |
|  |  |  | 453741 | 5 | 5340811 | 0.0938 |  |  |  | 13.5752 | 0.9276 | 0.4702 | 1.09E-29 |  |  | 6.366 |  |  |  |
|  |  |  | 453748 | 5 | 5341575 | 0.0942 |  |  |  |  |  |  |  | 2.67E-07 | 3.84E-07 | 34.816 |  |  |  |
|  |  |  | 454999 | 5 | 5642930 | 0.0424 |  |  |  | 4.015 | 0.5321 | 0.0082 |  |  |  | 262.734 |  |  |  |
|  | *GW5* | Os05g0187500 | 453756 | 5 | 5343949 | 0.0942 |  |  |  |  |  |  |  | 2.67E-07 | 3.84E-07 | 3.228 | 3.86E-15 | Liu et al. (2017) |  |
|  |  |  | 454125 | 5 | 5439809 | 0.2637 | 8.2616 | 0.4174 | 0.613 |  |  |  |  |  |  | 91.053 |  |  | ✓ |
|  |  |  | 453741 | 5 | 5340811 | 0.0938 |  |  |  | 13.5752 | 0.9276 | 0.4702 | 1.09E-29 |  |  | 6.366 |  |  |  |
|  | *OsAGSW1* | Os05g0323800 | 474548 | 5 | 14898923 | 0.3798 |  |  |  | 9.6761 | -0.5181 | 1.464 |  |  |  | 120.861 | 2.45E-45 | Li et al. (2015b) | ✓ |
|  | *D1* | Os05g0333200 | 476938 | 5 | 15556574 | 0.335 |  |  |  |  |  |  | 6.80E-07 |  |  | 30.729 | 9.81E-09 | Sun et al. (2018) | ✓ |
|  |  |  | 477387 | 5 | 15686963 | 0.3429 | 3.2963 | 0.2003 | 0.1947 |  |  |  |  |  |  | 76.126 |  |  |  |
|  | *OsS40-14* | Os05g0531000 | 513640 | 5 | 26579464 | 0.204 | 3.1496 | -0.1347 | 0.0488 |  |  |  |  |  |  | 151.131 | 2.04E-66 | Habiba et al. (2021) | ✓ |
|  | *OsPUP7* | Os05g0556800 | 516924 | 5 | 27904412 | 0.2644 | 8.2257 | 0.5123 | 0.8465 | 10.2945 | 0.5609 | 0.3739 |  |  |  | 131.391 | 4.99E-67 | Qi & Xiong (2013) | ✓ |
|  | *PFPβ* | Os06g0247500 | 551081 | 6 | 7856588 | 0.4225 |  |  |  |  |  |  | 2.56E-08 |  |  | 203.201 | 4.29E-24 | Duan et al. (2016) | ✓ |
|  | *OsNF-YB9* | Os06g0285200 | 556082 | 6 | 9887813 | 0.2411 | 4.674 | 0.2346 | 0.189 |  |  |  |  |  |  | 249.011 | 1.38E-33 | Das et al. (2019) | ✓ |
|  | *GL6* | Os06g0666100 | 597491 | 6 | 28432934 | 0.3256 | 3.6293 | -0.2004 | 0.1624 |  |  |  |  |  |  | 1.251 | 6.93E-10 | Wang et al. (2019) | ✓ |
|  |  |  | 597879 | 6 | 28713231 | 0.0306 |  |  |  |  |  |  | 4.24E-09 |  |  | 274.631 |  |  |  |
|  | *SNB* | Os07g0235800 | 625783 | 7 | 7750524 | 0.3617 | 5.0436 | -0.3564 | 0.2666 |  |  |  |  |  |  | 168.454 | 5.97E-21 | Jiang et al. (2019) | ✓ |
|  | *OsGASR9* | Os07g0592000 | 686960 | 7 | 24454607 | 0.2561 |  |  |  | 19.2585 | 0.7515 | 0.871 |  |  |  | 330.883 | 3.10E-11 | Li et al. (2019) | ✓ |
|  |  |  | 687032 | 7 | 24480143 | 0.2557 | 18.1798 | 0.696 | 1.6646 |  |  |  |  |  |  | 305.347 |  |  |  |
|  |  |  | 689017 | 7 | 24942633 | 0.3127 |  |  |  |  |  |  | 6.78E-09 |  |  | 154.989 |  |  |  |
|  | *SPL29* | Os08g0206900 | 724316 | 8 | 6215407 | 0.1015 |  |  |  | 10.4576 | -0.8486 | 0.2498 |  |  |  | 21.047 | 4.65E-25 | Wang et al. (2021) | ✓ |
|  | *qGW8* | Os08g0531600 | 768150 | 8 | 26356261 | 0.0632 |  |  |  |  |  |  | 1.67E-10 |  |  | 232.911 | 7.32E-42 | Wang et al. (2012) | ✓ |
|  | *OsSPL18* | Os09g0507100 | 834624 | 9 | 20291043 | 0.0615 |  |  |  | 7.7002 | -0.9038 | 0.0238 |  |  |  | 6.133 | 4.16E-41 | Yuan et al. (2019) | ✓ |
|  |  |  | 835521 | 9 | 20474830 | 0.2158 | 5.9415 | -0.3706 | 0.4494 |  |  |  |  |  |  | 172.884 |  |  |  |
|  | *OsPLGG1b* | Os10g0578800 | 914683 | 10 | 23514170 | 0.0563 | 5.2754 | -0.4823 | 0.2572 |  |  |  |  |  |  | 16.501 | 1.08E-04 | Cui et al. (2021) | ✓ |
|  | *OsSMK1* | Os11g0213500 | 934293 | 11 | 5554478 | 0.2832 | 6.1835 | -0.4661 | 0.6545 |  |  |  |  |  |  | 323.813 | 1.85E-50 | Li et al. (2014b) | ✓ |

*Note*: The known genes were confirmed by haplotype analysis, and *P* value of haplotype test is obtained from ANOVA for the traits of interest across various haplotypes. ATAC-seq dataset (Xie et al., 2021) was derived from http://glab.hzau.edu.cn/RiceENCODE/, in which the genes with open chromation regions were marked by “✓”. Chr, Chromosome.

**References**

1. Bai J, Zhu X, Wang Q, Zhang J, Chen H, Dong G, et al. Rice *TUTOU1* encodes a suppressor of cAMP receptor-like protein that is important for actin organization and panicle development. Plant Physiol 2015;169:1179–91.
2. Wang M, Zhu X, Peng G, Liu M, Zhang S, Chen M, et al. Methylesterification of cell-wall pectin controls the diurnal flower-opening times in rice. Mol Plant 2022;15:956–72.
3. Fu C, Yang XO, Chen X, Chen W, Ma Y, Hu J, et al. *OsEF3*, a homologous gene of Arabidopsis *ELF3*, has pleiotropic effects in rice. Plant Biol (Stuttg) 2009;11:751–7.
4. Zhang L, Zhang F, Zhou X, Poh TX, Xie L, Shen J, et al. The tetratricopeptide repeat protein OsTPR075 promotes heading by regulating florigen transport in rice. Plant Cell 2022b;34:3632–46.
5. Cai M, Zhu S, Wu M, Zheng X, Wang J, Zhou L, et al. DHD4, a CONSTANS-like family transcription factor, delays heading date by affecting the formation of the FAC complex in rice. Mol Plant 2021;14:330–43.
6. Chen F, Dong G, Wu L, Wang F, Yang X, Ma X, et al. A nucleus-encoded chloroplast protein YL1 is involved in chloroplast development and efficient biogenesis of chloroplast ATP synthase in rice. Sci Rep 2016a;6:32295.
7. Song S, Chen Y, Liu L, Wang Y, Bao S, Zhou X, et al. OsFTIP1-mediated regulation of florigen transport in rice is negatively regulated by the ubiquitin-like domain kinase OsUbDKγ4. The Plant Cell 2017;29:491–507.
8. Zhao XL, Shi ZY, Peng LT, Shen GZ, Zhang JL. An atypical HLH protein OsLF in rice regulates flowering time and interacts with OsPIL13 and OsPIL15. Nat Biotechnol 2011;28:788–97.
9. Sun X, Zhang Z, Wu J, Cui X, Feng D, Wang K, et al. The *Oryza sativa* Regulator HDR1 Associates with the Kinase *OsK4* to Control Photoperiodic Flowering. PLoS Genet 2016;12:e1005927.
10. Tadege M, Sheldon CC, Helliwell CA, Upadhyaya NM, Dennis ES, Peacock WJ, et al. Reciprocal control of flowering time by *OsSOC1* in transgenic Arabidopsis and by *FLC* in transgenic rice. Plant Biotechnol J 2003;1:361–9.
11. Li D, Yang C, Li X, Gan Q, Zhao X, Zhu L. Functional characterization of rice *OsDof12*. *Planta* 2009;229:1159–69.
12. Lee S, Kim J, Han JJ, Han MJ, An G. Functional analyses of the flowering time gene *OsMADS50*, the putative *SUPPRESSOR OF OVEREXPRESSION OF CO 1/AGAMOUS-LIKE 20 (SOC1/AGL20)* ortholog in rice. Plant J 2004;38:754–64.
13. Jiang P, Wang S, Jiang H, Cheng B, Wu K, Ding Y, et al. The COMPASS-Like complex promotes flowering and panicle branching in rice. Plant Physiol 2018b;176:2761–71.
14. Vega-Sánchez ME, Zeng L, Chen S, Leung H, Wang GL. SPIN1, a K homology domain protein negatively regulated and ubiquitinated by the E3 ubiquitin ligase SPL11, is involved in flowering time control in rice. The Plant Cell 2008;20:1456–69.
15. Wuriyanghan H, Zhang B, Cao WH, Ma B, Lei G, Liu YF, et al. The ethylene receptor *ETR2* delays floral transition and affects starch accumulation in rice. The Plant Cell 2009;21:1473–94.
16. Cho LH, Yoon J, Pasriga R, An G. Homodimerization of Ehd1 is required to induce flowering in rice. Plant Physiol 2016;170:2159–71.
17. Bai B, Zhao J, Li Y, Zhang F, Zhou J, Chen F, et al. *OsBBX14* delays heading date by repressing florigen gene expression under long and short-day conditions in rice. Plant Sci 2016;247:25–34.
18. Hu L, Ye M, Li R, Zhang T, Zhou G, Wang Q, et al. The rice transcription factor *WRKY53* suppresses herbivore-induced defenses by acting as a negative feedback modulator of mitogen-activated protein kinase activity. Plant Physiol 2015b;169:2907–21.
19. Qi Z, Xiong L. Characterization of a purine permease family gene *OsPUP7* involved in growth and development control in rice. J Integr Plant Biol 2013;55:1119–35.
20. Wang L, Yin H, Qian Q, Yang J, Huang C, Hu X, et al. *NECK LEAF 1*, a *GATA* type transcription factor, modulates organogenesis by regulating the expression of multiple regulatory genes during ren in rice. Nat Commun 2009;9:735.
21. Matsubara K, Ogiso-Tanaka E, Hori K, Ebana K, Ando T, Yano M. Natural variation in *Hd17*, a homolog of Arabidopsis *ELF3* that is involved in rice photoperiodic flowering. Plant Cell Physiol 2012;53:709–16.
22. Takahashi Y, Teshima KM, Yokoi S, Innan H, Shimamoto K. Variations in *Hd1* proteins, *Hd3a* promoters, and *Ehd1* expression levels contribute to diversity of flowering time in cultivated rice. P Natl Acad Sci Usa 2009;106:4555–60.
23. Zhu QH, Ramm K, Shivakkumar R, Dennis ES, Upadhyaya NM. The *ANTHER INDEHISCENCE_1_* gene encoding a single MYB domain protein is involved in anther development in rice. Plant Physiol 2004;135:1514–25.
24. Das S, Parida SK, Agarwal P, Tyagi AK. Transcription factor *OsNF-YB9* regulates reproductive growth and development in rice. Planta 2019;250:1849–65.
25. Andrés F, Galbraith DW, Talón M, Domingo C. Analysis of *PHOTOPERIOD SENSITIVITY5* sheds light on the role of phytochromes in photoperiodic flowering in rice. Plant Physiol 2009;151:681–90.
26. Hong Y, Zhang Y, Sinumporn S, Yu N, Zhan X, Shen X. Premature leaf senescence 3, encoding a methyltransferase, is required for melatonin biosynthesis in rice. Plant J 2018;95:877–91
27. Xue W, Xing Y, Weng X, Zhao Y, Tang W, Wang L, et al. Natural variation in *Ghd7* is an important regulator of heading date and yield potential in rice. Nat Genet 2008;40:761–7.
28. Fornara F, Parenicová L, Falasca G, Pelucchi N, Masiero S, Ciannamea S, et al. Functional characterization of *OsMADS18*, a member of the *AP1/SQUA* subfamily of MADS box genes. Plant Physiol 2004;135:2207–19.
29. Sun C, Zhang K, Zhou Y, Xiang L, He C, Zhong C, et al. Dual function of clock component *OsLHY* sets critical day length for photoperiodic flowering in rice. Plant Biotechnol J 2021;19:1644–57.
30. Dai X, Ding Y, Tan L, Fu Y, Liu F, Zhu Z, et al. *LHD1*, an allele of *DTH8/Ghd8*, controls late heading date in common wild rice (*Oryza rufipogon*). J Integr Plant Biol 2012;54:790–9.
31. Jeong HJ, Yang J, Cho LH, An G. *OsVIL1* controls flowering time in rice by suppressing *OsLF* under short days and by inducing *Ghd7* under long days. Plant Cell Rep 2016;35:905–20.
32. Guo S, Dai S, Singh PK, Wang H, Wang Y, Tan JLH, et al. A membrane-bound NAC-like transcription factor *OsNTL5* represses the flowering in *Oryza sativa*. Front Plant Sci 2018a;9:555.
33. Lee JH, Cho YS, Yoon HS, Suh MC, Moon J, Lee I, et al. Conservation and divergence of *FCA* function between *Arabidopsis* and rice. Plant Mol Biol 2005;58:823–38.
34. Kim SK, Yun CH, Lee JH, Jang YH, Park HY, Kim JK. *OsCO3*, a *CONSTANS-LIKE* gene, controls flowering by negatively regulating the expression of FT-like genes under SD conditions in rice. Planta 2008;228:355–65.
35. Chen SY, Wang ZY, Cai XL. *OsRRM*, a spen-like rice gene expressed specifically in the endosperm. Cell Res 2007b;17:713–21.
36. Li X, Tian X, He M, Liu X, Li Z, Tang J, et al. *bZIP71* delays flowering by suppressing *Ehd1* expression in rice. J Integr Plant Biol 2022b;64:1352–63.
37. Xie S, Chen M, Pei R, Ouyang Y, Yao J. *OsEMF2b* acts as a regulator of flowering transition and floral organ identity by mediating *H3K27me3* deposition at *OsLFL1* and *OsMADS4* in rice. Plant Mol Biol Rep 2015;33:121–132.
38. Kang HG, Jang S, Chung JE, Cho YG, An G. Characterization of two rice MADS box genes that control flowering time. Mol Cells 1997;**7**:559–66.
39. Chen R, Zhao X, Shao Z, Wei Z, Wang Y, Zhu L, et al. Rice UDP-glucose pyrophosphorylase1 is essential for pollen callose deposition and its cosuppression results in a new type of thermosensitive genic male sterility. Plant Cell 2007a;19:847–61.
40. Jiang P, Wang S, Zheng H, Li H, Zhang F, Su Y, et al. SIP1 participates in regulation of flowering time in rice by recruiting *OsTrx1* to *Ehd1*. New Phytol 2018c;219:422-35.
41. Lu H, Dai Z, Li L, Wang J, Miao X, Shi Z. *OsRAMOSA2* shapes panicle architecture through regulating pedicel length. Front Plant Sci 2017;8:1538.
42. Liu Y, Chen X, Xue S, Quan T, Cui D, Han L, et al. *SET DOMAIN GROUP 721* protein functions in saline-alkaline stress tolerance in the model rice variety Kitaake. Plant Biotechnol J 2021b;19:2576–88.
43. Iwamoto M, Higo K, Takano M. Circadian clock- and phytochrome-regulated dof-like gene, Rdd1, is associated with grain size in rice. Plant Cell Environ 2009;32:592–603.
44. Chan AN, Wang LL, Zhu YJ, Fan YY, Zhuang JY, Zhang ZH. Identification through fine mapping and verification using CRISPR/*Cas9*-targeted mutagenesis for a minor QTL controlling grain weight in rice. Theor Appl Genet 2021;134:327–37.
45. Su’udi M, Cha JY, Ahn IP, Kwak YS, Woo YM, Son D. Functional characterization of a B-type cell cycle switch 52 in rice (*OsCCS52B*). Plant Cell Tiss Org 2012;111:101–11.
46. Li Z, Wei X, Tong X, Zhao J, Liu X, Wang H, et al. The *OsNAC23-Tre6P-SnRK1a* feed-forward loop regulates sugar homeostasis and grain yield in rice. Mol Plant 2022c;15:706–22.
47. Hao J, Wang D, Wu Y, Huang K, Duan P, Li N, et al. The *GW2-WG1-OsbZIP47* pathway controls grain size and weight in rice. Mol Plant 2021;14:1266–80.
48. Lyu J, Wang D, Duan P, Liu Y, Huang K, Zeng D, et al. Control of grain size and weight by the GSK2-LARGE1/OML4 pathway in rice. Plant Cell 2020;32:1905–18.
49. Hu X, Qian Q, Xu T, Zhang Y, Dong G, Gao T, et al. The U-box E3 ubiquitin ligase TUD1 functions with a heterotrimeric G α subunit to regulate brassinosteroid-mediated growth in rice. PLoS Genet 2013;9:e1003391.
50. Fan C, Yu S, Wang C, Xing Y. A causal C-A mutation in the second exon of GS3 highly associated with rice grain length and validated as a functional marker. Theor Appl Genet 2009;118: 465–72.
51. Xu F, Fang J, Ou S, Gao S, Zhang F, Du L, et al. Variations in *CYP78A13* coding region influence grain size and yield in rice. Plant Cell Environ 2015;38:800–11.
52. Sun S, Wang L, Mao H, Shao L, Li X, Xiao J, et al*.* A G-protein pathway determines grain size in rice. Nat Commun 2018;9:851.
53. Zhang Z, Zhao H, Huang F, Long J, Song G, Lin W. The 14-3-3 protein GF14f negatively affects grain filling of inferior spikelets of rice (*Oryza sativa* L.). Plant J 2019b;99:344–58.
54. Shimano S, Hibara KI, Furuya T, Arimura SI, Tsukaya H, Itoh JI. Conserved functional control, but distinct regulation, of cell proliferation in rice and arabidopsis leaves revealed by comparative analysis of *GRF-INTERACTING FACTOR 1* orthologs. Development 2018;145:dev159624
55. Ying JZ, Ma M, Bai C, Huang XH, Liu JL, Fan YY, et al. *TGW3*, a major QTL that negatively modulates grain length and weight in rice. Mol Plant 2018;11:750–3.
56. Zhang H, Lu Y, Zhao Y, Zhou DX. OsSRT1 is involved in rice seed development through regulation of starch metabolism gene expression. Plant Sci 2016b;248:28–36.
57. Yin W, Xiao Y, Niu M, Meng W, Li L, Zhang X, et al. *ARGONAUTE2* enhances grain length and salt tolerance by activating BIG GRAIN3 to modulate cytokinin distribution in rice. Plant Cell 2020;32:2292–306.
58. Guo T, Chen K, Dong NQ, Shi CL, Ye WW, Gao JP, et al. *GRAIN SIZE AND NUMBER1* negatively regulates the *OsMKKK10-OsMKK4-OsMPK6* cascade to coordinate the trade-off between grain number per panicle and grain size in rice. Plant Cell 2018b;30:871–88.
59. Kitagawa K, Kurinami S, Oki K, Abe Y, Ando T, Kono I, et al. A novel kinesin 13 protein regulating rice seed length. Plant Cell Physiol 2010;51:1315–29.
60. Yang C, Ma Y, He Y, Tian Z, Li J. OsOFP19 modulates plant architecture by integrating the cell division pattern and brassinosteroid signaling. Plant J 2018;93:489–501.
61. Wei S, Li X, Lu Z, Zhang H, Ye X, Zhou Y, et al. A transcriptional regulator that boosts grain yields and shortens the growth duration of rice. Science 2022;377:370–1.
62. Zhao YF, Peng T, Sun HZ, Teotia S, Wen HL, Du YX, et al. miR1432-OsACOT (Acyl-CoA thioesterase) module determines grain yield via enhancing grain filling rate in rice. Plant Biotechnol J 2019;17:712–23.
63. Guo T, Lu ZQ, Shan JX, Ye WW, Dong NQ, Lin HX. ERECTA1 acts upstream of the *OsMKKK10-OsMKK4-OsMPK6* cascade to control spikelet number by regulating cytokinin metabolism in rice. Plant Cell 2020;32:2763–79.
64. Tanaka A, Nakagawa H, Tomita C, Shimatani Z, Ohtake M, Nomura T, et al. BRASSINOSTEROID UPREGULATED1, encoding a helix-loop-helix protein, is a novel gene involved in brassinosteroid signaling and controls bending of the lamina joint in rice. Plant Physiol 2009;151:669–80.
65. Wang A, Hou Q, Si L, Huang X, Luo J, Lu D, et al. The PLATZ transcription factor GL6 affects grain length and number in rice. Plant Physiol 2019;180:2077–90.
66. Yang B, Wendrich JR, De Rybel B, Weijers D, Xue HW. Rice microtubule-associated protein IQ67-DOMAIN14 regulates grain shape by modulating microtubule cytoskeleton dynamics. Plant Biotechnol J 2020;18:1141–52.
67. Li Z, Tang L, Qiu J, Zhang W, Wang Y, Tong X, et al. Serine carboxypeptidase 46 regulates grain filling and seed germination in rice (*Oryza sativa* L.). PLoS One 2016;11:e0159737.
68. Zhan P, Ma S, Xiao Z, Li F, Wei X, Lin S, et al. Natural variations in grain length 10 (*GL10*) regulate rice grain size. J Genet Genomics 2022;49:405–13.
69. Cho SH, Yoo SC, Zhang H, Pandeya D, Koh HJ, Hwang JY, et al. The rice narrow leaf2 and narrow leaf3 loci encode WUSCHEL-related homeobox 3A (*OsWOX3A*) and function in leaf, spikelet, tiller and lateral root development. New Phytol 2013;198:1071–84.
70. Qi P, Lin YS, Song XJ, Shen JB, Huang W, Shan JX, et al. The novel quantitative trait locus GL3.1 controls rice grain size and yield by regulating *Cyclin-T1;3*. Cell Res 2012;22:1666–80.
71. Segami S, Kono I, Ando T, Yano M, Kitano H, Miura K, et al. Small and round seed 5 gene encodes alpha-tubulin regulating seed cell elongation in rice. Rice 2012;5:4.
72. Hong Y, Liu Q, Cao Y, Zhang Y, Chen D, Lou X, et al. The OsMPK15 negatively regulates magnaporthe oryza and xoo disease resistance via SA and JA signaling pathway in rice. Front Plant Sci 2019;10:752.
73. Zhang X, Wang J, Huang J, Lan H, Wang C, Yin C, et al. Rare allele of *OsPPKL1* associated with grain length causes extra-large grain and a significant yield increase in rice. Proc Natl Acad Sci U S A 2012;109:21534–9.
74. Wang S, Lei C, Wang J, Ma J, Tang S, Wang C, et al. SPL33, encoding an eEF1A-like protein, negatively regulates cell death and defense responses in rice. J Exp Bot 2017;68:899–913.
75. Hou J, Cao C, Ruan Y, Deng Y, Liu Y, Zhang K, et al. *ESA1* is involved in embryo sac abortion in interspecific hybrid progeny of rice. Plant Physiol 2019;180:356–66.
76. Ramegowda V, Basu S, Krishnan A, Pereira A. Rice *GROWTH UNDER DROUGHT KINASE* is required for drought tolerance and grain yield under normal and drought stress conditions. Plant Physiol 2014;166:1634–45.
77. Hu J, Zeng T, Xia Q, Huang L, Zhang Y, Zhang C, et al. Identification of key genes for the ultrahigh yield of rice using dynamic cross-tissue network analysis. Genomics Proteomics Bioinformatics 2020;18:256–70.
78. Kumar M, Choi J, An G, Kim SR. Ectopic expression of *OsSta2* enhances salt stress tolerance in rice. Front Plant Sci 2017;8:316.
79. Zhou B, Lin JZ, Peng D, Yang YZ, Guo M, Tang DY, et al. Plant architecture and grain yield are regulated by the novel DHHC-type zinc finger protein genes in rice (Oryza sativa L.). Plant Sci 2017a;254:12–21.
80. Lin Y, Tan L, Zhao L, Sun X, Sun C. RLS3, a protein with AAA+ domain localized in chloroplast, sustains leaf longevity in rice. J Integr Plant Biol 2016;58:971–82.
81. Zhang H, Zhou JF, Kan Y, Shan JX, Ye WW, Dong NQ, et al. A genetic module at one locus in rice protects chloroplasts to enhance thermotolerance. Science 2022a;376:1293–1300.
82. Lei J, Teng X, Wang Y, Jiang X, Zhao H, Zheng X, et al. Plastidic pyruvate dehydrogenase complex E1 component subunit Alpha1 is involved in galactolipid biosynthesis required for amyloplast development in rice. Plant Biotechnol J 2022;20:437–53.
83. Wu Z, Zhang X, He B, Diao L, Sheng S, Wang J, et al. A chlorophyll-deficient rice mutant with impaired chlorophyllide esterification in chlorophyll biosynthesis. Plant Physiol 2007;145:29–40.
84. Huang X, Yang S, Gong J, Zhao Y, Feng Q, Gong H, et al. Genomic analysis of hybrid rice varieties reveals numerous superior alleles that contribute to heterosis. Nat Commun 2015;6:6258.
85. Wang Q, Nian J, Xie X, Yu H, Zhang J, Bai J, et al. Genetic variations in ARE1 mediate grain yield by modulating nitrogen utilization in rice. Nat Commun 2018;9,735.
86. Yuan H, Qin P, Hu L, Zhan S, Wang S, Gao P, et al. *OsSPL18* controls grain weight and grain number in rice. J Genet Genomics 2019;46:41–51.
87. Ashikari M, Sakakibara H, Lin S, Yamamoto T, Takashi T, Nishimura A, et al. Cytokinin oxidase regulates rice grain production. Science 2005;309:741–5.
88. Jiang P, Wang S, Ikram AU, Xu Z, Jiang H, Cheng B, et al. *SDG721* and *SDG705* are required for rice growth. J Integr Plant Biol 2018a;60:530–5.
89. Shin NH, Trang DT, Hong WJ, Kang K, Chuluuntsetseg J, Moon JK, et al. Rice senescence-induced receptor-like kinase (OsSRLK) is involved in phytohormone-mediated chlorophyll degradation. Int J Mol Sci 2019;21:260.
90. Borna RS, Murchie EH, Pyke KA, Roberts JA, Gonzalez-Carranza ZH. The rice *EP3* and *OsFBK1 E3* ligases alter plant architecture and flower development, and affect transcript accumulation of microRNA pathway genes and their targets. Plant Biotechnol J 2022;20:297–309.
91. Li G, Xu B, Zhang Y, Xu Y, Khan NU, Xie J, et al. *RGN1* controls grain number and shapes panicle architecture in rice. Plant Biotechnol J 2022a;20:158–67.
92. Zhang F, Zhang YC, Liao JY, Yu Y, Zhou YF, Feng YZ, et al. The subunit of RNA N6-methyladenosine methyltransferase *OsFIP* regulates early degeneration of microspores in rice. PLoS Genet 2019a;15:e1008120.
93. Lv XG, Shi YF, Xu X, Wei YL, Wang HM, Zhang XB, et al. *Oryza sativa* chloroplast signal recognition particle 43 (*OscpSRP43*) is required for chloroplast development and photosynthesis. PLoS One 2015;10: e0143249.
94. Li C, Zhu S, Zhang H, Chen L, Cai M, Wang J, et al. OsLBD37 and OsLBD38, two class II type LBD proteins, are involved in the regulation of heading date by controlling the expression of Ehd1 in rice. Biochem Biophys Res Commun 2017;486:720–5.
95. Li C, Hu Y, Huang R, Ma X, Wang Y, Liao T, et al. Mutation of *FdC2* gene encoding a ferredoxin-like protein with C-terminal extension causes yellow-green leaf phenotype in rice. Plant Sci 2015a;238:127–34.
96. Yano K, Ookawa T, Aya K, Ochiai Y, Hirasawa T, Ebitani T, et al. Isolation of a novel lodging resistance QTL gene involved in strigolactone signaling and its pyramiding with a QTL gene involved in another mechanism. Mol Plant 2015;8:303–14.
97. Yaish MW, El-Kereamy A, Zhu T, Beatty PH, Good AG, Bi YM, et al. The APETALA-2-like transcription factor *OsAP2-39* controls key interactions between abscisic acid and gibberellin in rice. PLoS Genet 2010;6: e1001098.
98. Mjomba FM, Zheng Y, Liu H, Tang W, Hong Z, Wang F, et al. Homeobox is pivotal for *OsWUS* controlling tiller development and female fertility in rice. G3 (Bethesda) 2016;6:2013–21.
99. Qian W, Wu C, Fu Y, Hu G, He Z, Liu W. Novel rice mutants overexpressing the brassinosteroid catabolic gene *CYP734A4*. Plant Mol Biol 2017;93:197–208.
100. Zhao M, Liu B, Wu K, Ye Y, Huang S, Wang S, et al. Regulation of *OsmiR156h* through alternative polyadenylation improves grain yield in rice. PLoS One 2015;10:e0126154.
101. Guo M, Ruan W, Li C, Huang F, Zeng M, Liu Y, et al. Integrative comparison of the role of the *PHOSPHATE RESPONSE1* subfamily in phosphate signaling and homeostasis in rice. Plant Physiol 2015;168: 1762–76.
102. Xu W, Tao J, Chen M, Dreni L, Luo Z, Hu Y, et al. Interactions between *FLORAL ORGAN NUMBER4* and floral homeotic genes in regulating rice flower development. J Exp Bot 2017;68:483–98.
103. Luan W, Liu Y, Zhang F, Song Y, Wang Z, Peng Y, et al. *OsCD1* encodes a putative member of the cellulose synthase-like D sub-family and is essential for rice plant architecture and growth. Plant Biotechnol J 2011;9:513–24.
104. Liu D, Yu Z, Zhang G, Yin W, Li L, Niu M, et al. Diversification of plant agronomic traits by genome editing of brassinosteroid signaling family genes in rice. Plant Physiol 2021a;187:2563–76.
105. Wei X, Qiu J, Yong K, Fan J, Zhang Q, Hua H, et al. A quantitative genomics map of rice provides genetic insights and guides breeding. Nat Genet 2021;53:243–53.
106. Yue ZL, Liu N, Deng ZP, Zhang Y, Wu ZM, Zhao JL, et al. The receptor kinase OsWAK11 monitors cell wall pectin changes to fine-tune brassinosteroid signaling and regulate cell elongation in rice. Curr Biol 2022;32:2454–66.
107. Yang X, Wu F, Lin X, Du X, Chong K, Gramzow L, et al. Live and let die - the B(sister) MADS-box gene *OsMADS29* controls the degeneration of cells in maternal tissues during seed development of rice (Oryza sativa). PLoS One 2012;7:e51435.
108. Xu JJ, Zhang XF, Xue HW. Rice aleurone layer specific *OsNF-YB1* regulates grain filling and endosperm development by interacting with an ERF transcription factor. J Exp Bot 2016;67:6399–411.
109. Wu M, Ren Y, Cai M, Wang Y, Zhu S, Zhu J, et al. Rice FLOURY ENDOSPERM10 encodes a pentatricopeptide repeat protein that is essential for the trans-splicing of mitochondrial nad1 intron 1 and endosperm development. New Phytol 2019;223:736–50.
110. Fan C, Xing Y, Mao H, Lu T, Han B, Xu C, et al. GS3, a major QTL for grain length and weight and minor QTL for grain width and thickness in rice, encodes a putative transmembrane protein. Theor Appl Genet 2006;112:1164–71.
111. Lee DK, Kim HI, Jang G, Chung PJ, Jeong JS, Kim YS, et al. The NF-YA transcription factor *OsNF-YA7* confers drought stress tolerance of rice in an abscisic acid independent manner. Plant Sci 2015;241:199–210.
112. Shang XL, Xie RR, Tian H, Wang QL, Guo FQ. Putative zeatin O-glucosyltransferase *OscZOG1* regulates root and shoot development and formation of agronomic traits in rice. J Integr Plant Biol 2016;58:627–41.
113. Zhu X, Liang W, Cui X, Chen M, Yin C, Luo Z, et al. Brassinosteroids promote development of rice pollen grains and seeds by triggering expression of carbon starved anther, a MYB domain protein. Plant J 2015;82:570–81.
114. Lan J, Lin Q, Zhou C, Ren Y, Liu X, Miao R, et al. Small grain and semi-dwarf 3, a *WRKY* transcription factor, negatively regulates plant height and grain size by stabilizing SLR1 expression in rice. Plant Mol Biol 2020;104:429–50.
115. Gui J, Liu C, Shen J, Li L. Grain setting defect1, encoding a remorin protein, affects the grain setting in rice through regulating plasmodesmatal conductance. Plant Physiol 2014;166:1463–78.
116. Qi J, Li J, Han X, Li R, Wu J, Yu H, et al. Jasmonic acid carboxyl methyltransferase regulates development and herbivory-induced defense response in rice. J Integr Plant Biol 2016;58:564–76.
117. Fu FF, Xue HW. Coexpression analysis identifies rice starch regulator1, a rice AP2/EREBP family transcription factor, as a novel rice starch biosynthesis regulator. Plant Physiol 2010;154:927–38.
118. Qian D, Chen G, Tian L, Qu LQ. *OsDER1* is an ER-associated protein degradation factor that responds to eR stress. Plant Physiol 2018;178:402–12.
119. Liu J, Chen J, Zheng X, Wu F, Lin Q, Heng Y, et al. *GW5* acts in the brassinosteroid signalling pathway to regulate grain width and weight in rice. Nat Plants 2017;3:17043.
120. Li T, Jiang J, Zhang S, Shu H, Wang Y, Lai J, et al. OsAGSW1, an ABC1-like kinase gene, is involved in the regulation of grain size and weight in rice. J Exp Bot 2015b;66:5691–701.
121. Habiba, Xu J, Gad AG, Luo Y, Fan C, Uddin JBG, et al. Five *OsS40* family members are identified as senescence-related genes in rice by reverse genetics approach. Front Plant Sci 2021;12:701529.
122. Duan E, Wang Y, Liu L, Zhu J, Zhong M, Zhang H, et al. Pyrophosphate: fructose-6-phosphate 1-phosphotransferase (*PFP*) regulates carbon metabolism during grain filling in rice. Plant Cell Rep 2016;35:1321–31.
123. Jiang L, Ma X, Zhao S, Tang Y, Liu F, Gu P, et al. The APETALA2-Like transcription factor SUPERNUMERARY BRACT controls rice seed shattering and seed size. Plant Cell 2019;31:17–36.
124. Li X, Shi S, Tao Q, Tao Y, Miao J, Peng X, et al. OsGASR9 positively regulates grain size and yield in rice (*Oryza sativa*). Plant Sci 2019;286:17–27.
125. Wang Z, Wang Q, Wei L, Shi Y, Li T, Hu K, et al. UDP-N-Acetylglucosamine pyrophosphorylase 2 (UAP2) and 1 (UAP1) perform synergetic functions for leaf survival in rice. Front Plant Sci 2021;12:685102.
126. Wang S, Wu K, Yuan Q, Liu X, Liu Z, Lin X, et al. Control of grain size, shape and quality by *OsSPL16* in rice. Nat Genet 2012;44:950–54.
127. Cui L, Zhang C, Li Z, Xian T, Wang L, Zhang Z, et al. Two plastidic glycolate/glycerate translocator 1 isoforms function together to transport photorespiratory glycolate and glycerate in rice chloroplasts. J Exp Bot 2021;72:2584–99.
128. Li XJ, Zhang YF, Hou M, Sun F, Shen Y, Xiu ZH, et al. Small kernel 1 encodes a pentatricopeptide repeat protein required for mitochondrial nad7 transcript editing and seed development in maize (*Zea mays*) and rice (*Oryza sativa*). Plant J 2014b;79:797–809.
129. Xie L, Liu M, Zhao L, Cao K, Wang P, Xu W, et al. RiceENCODE: A comprehensive epigenomic database as a rice Encyclopedia of DNA Elements. Mol Plant 2021;14:1604–06.
