# Supplementary material for: BLUPmrMLM: A Fast mrMLM Algorithm in Genome-wide Association Studies
Source: Genomics Proteomics Bioinformatics. 2024 Feb 29;22(3):qzae020. doi: 10.1093/gpbjnl/qzae020 (PMC12016565; doi:10.1093/gpbjnl/qzae020)
Supplement: qzae020_Supplementary_Data [file qzae020_supplementary_data.zip › Table S1.docx]

**Table S1** **Genotypic datasets used in simulation experiments and derived from Zhu et al. [44]**

| **Chromosome** | **Start (bp)** | **End (bp)** | **Number of markers** |
| --- | --- | --- | --- |
| 1 | 3,283,135 | 22,351,140 | 5000 |
| 2 | 7,152,422 | 26,145,771 | 5000 |
| 3 | 9,930,557 | 27,991,129 | 5000 |
| 4 | 15,313,128 | 35,946,074 | 5000 |
| 5 | 19,868,450 | 39,293,694 | 5000 |
| 6 | 23,607,940 | 42,169,746 | 5000 |
| 7 | 28,929,867 | 46,653,312 | 5000 |
| 8 | 29,532,712 | 49,159,446 | 5000 |
| 9 | 35,938,453 | 56,960,713 | 5000 |
| 10 | 36,869,383 | 56,007,830 | 5000 |

[44] Zhu B, Zhu M, Jiang J, Niu H, Wang Y, Wu Y, et al. The impact of variable degrees of freedom and scale parameters in Bayesian methods for genomic prediction in Chinese Simmental beef cattle. PLoS One 2016;11:e0154118.
