# Supplementary figures and images for: BLUPmrMLM: A Fast mrMLM Algorithm in Genome-wide Association Studies
Source: Genomics Proteomics Bioinformatics. 2024 Feb 29;22(3):qzae020. doi: 10.1093/gpbjnl/qzae020 (PMC12016565; doi:10.1093/gpbjnl/qzae020)

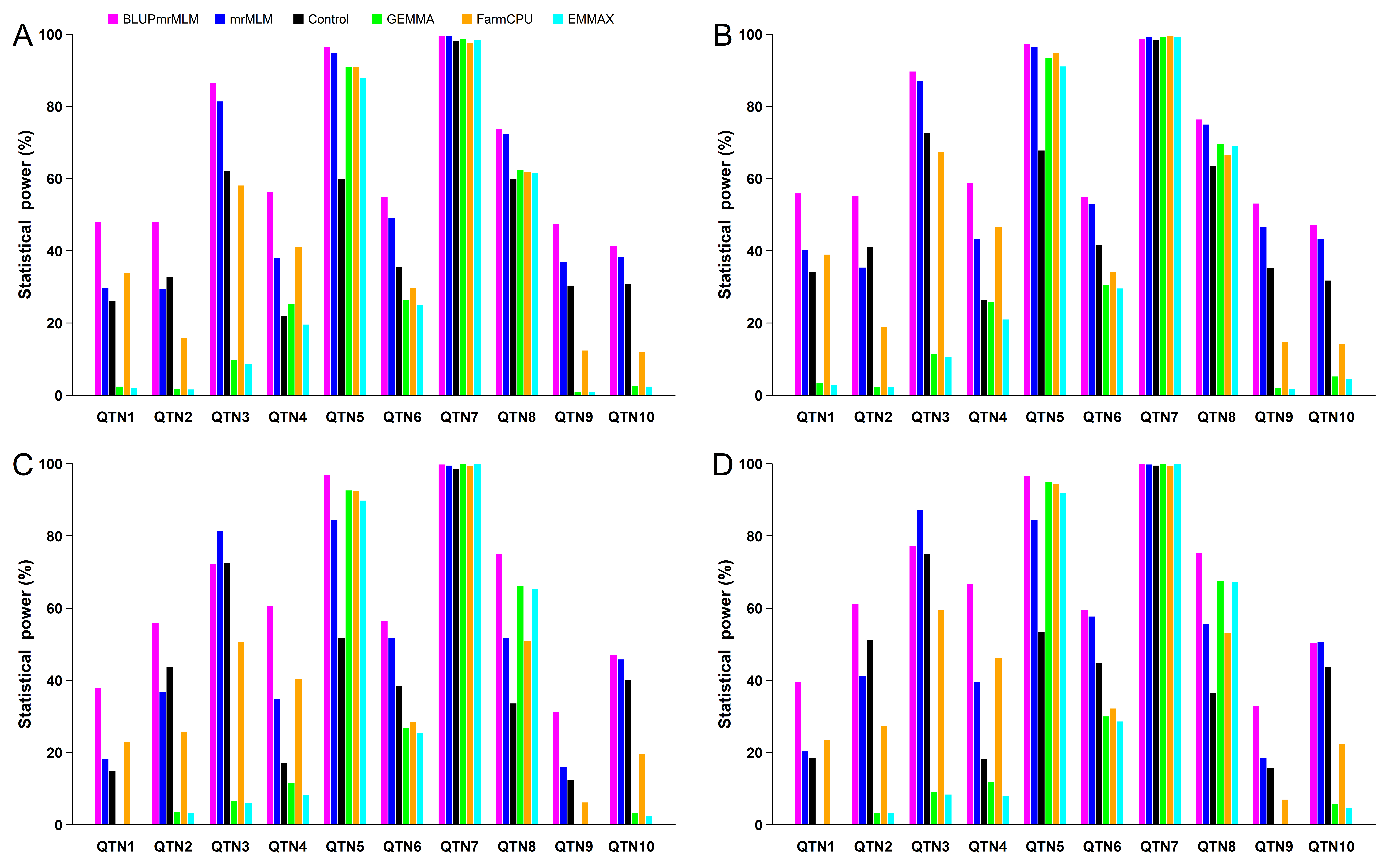

Supplement: qzae020_Supplementary_Data [file qzae020_supplementary_data.zip › Figure S1.tiff]

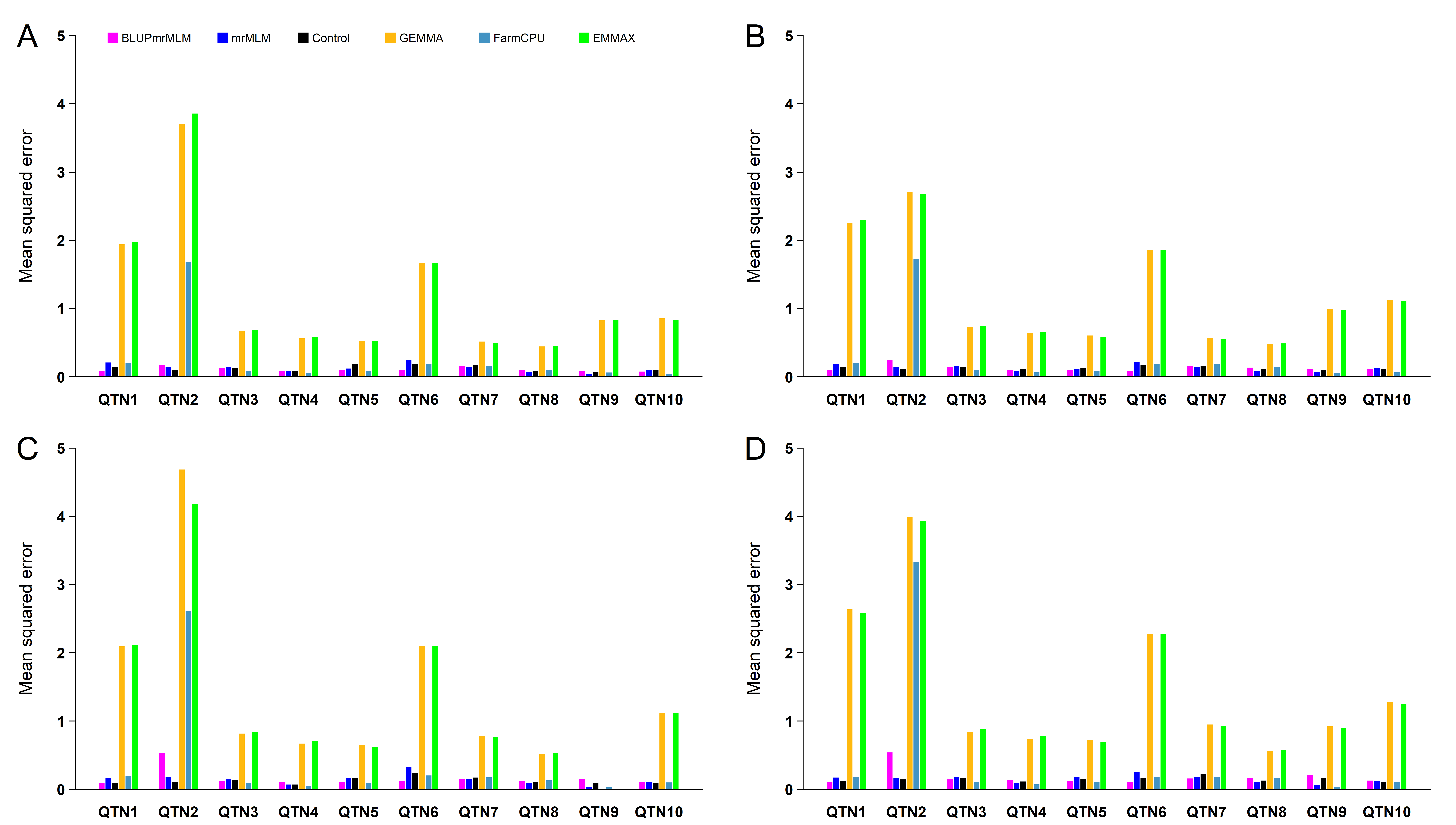

Supplement: qzae020_Supplementary_Data [file qzae020_supplementary_data.zip › Figure S3.tiff]

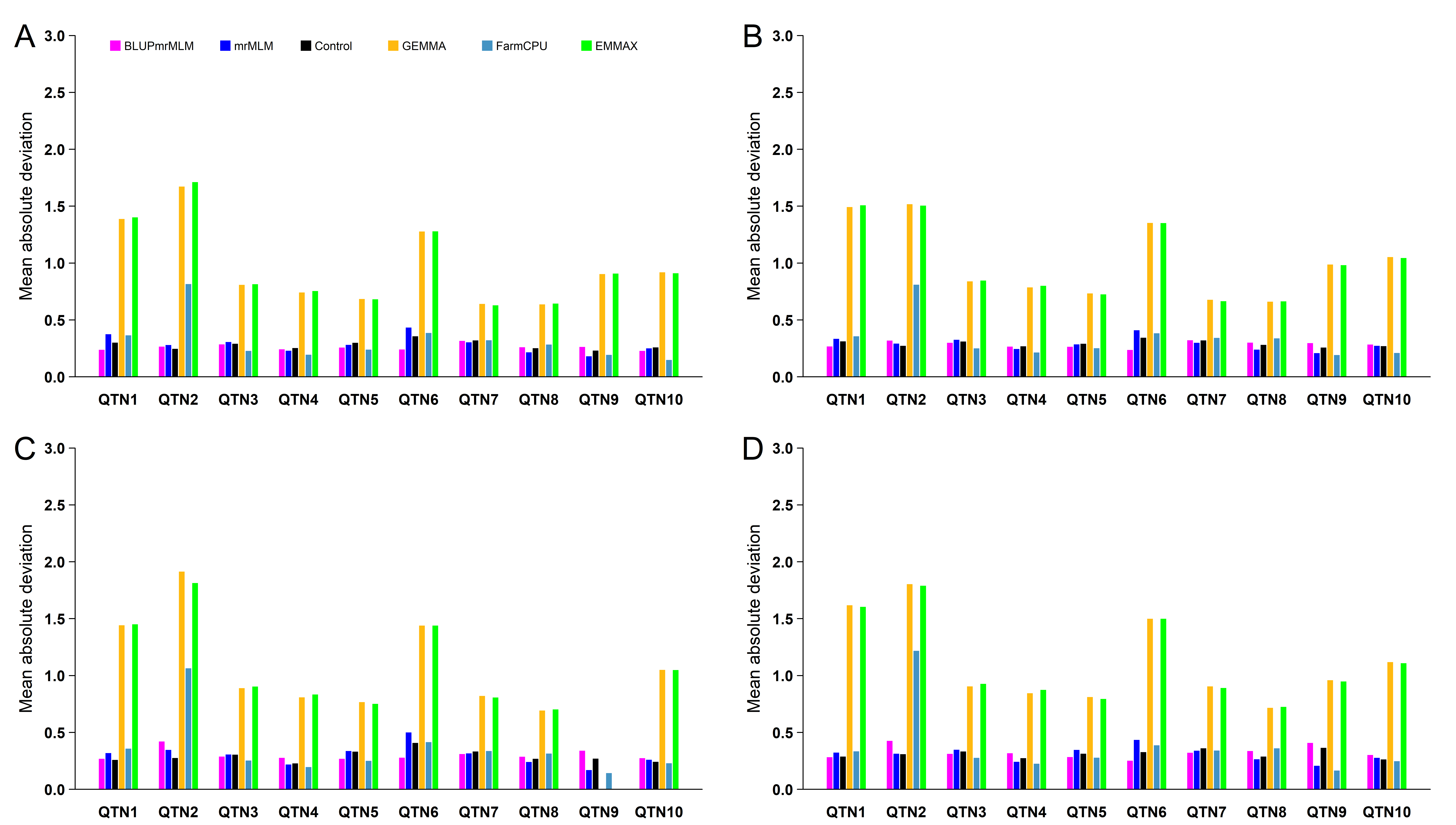

Supplement: qzae020_Supplementary_Data [file qzae020_supplementary_data.zip › Figure S4.tiff]

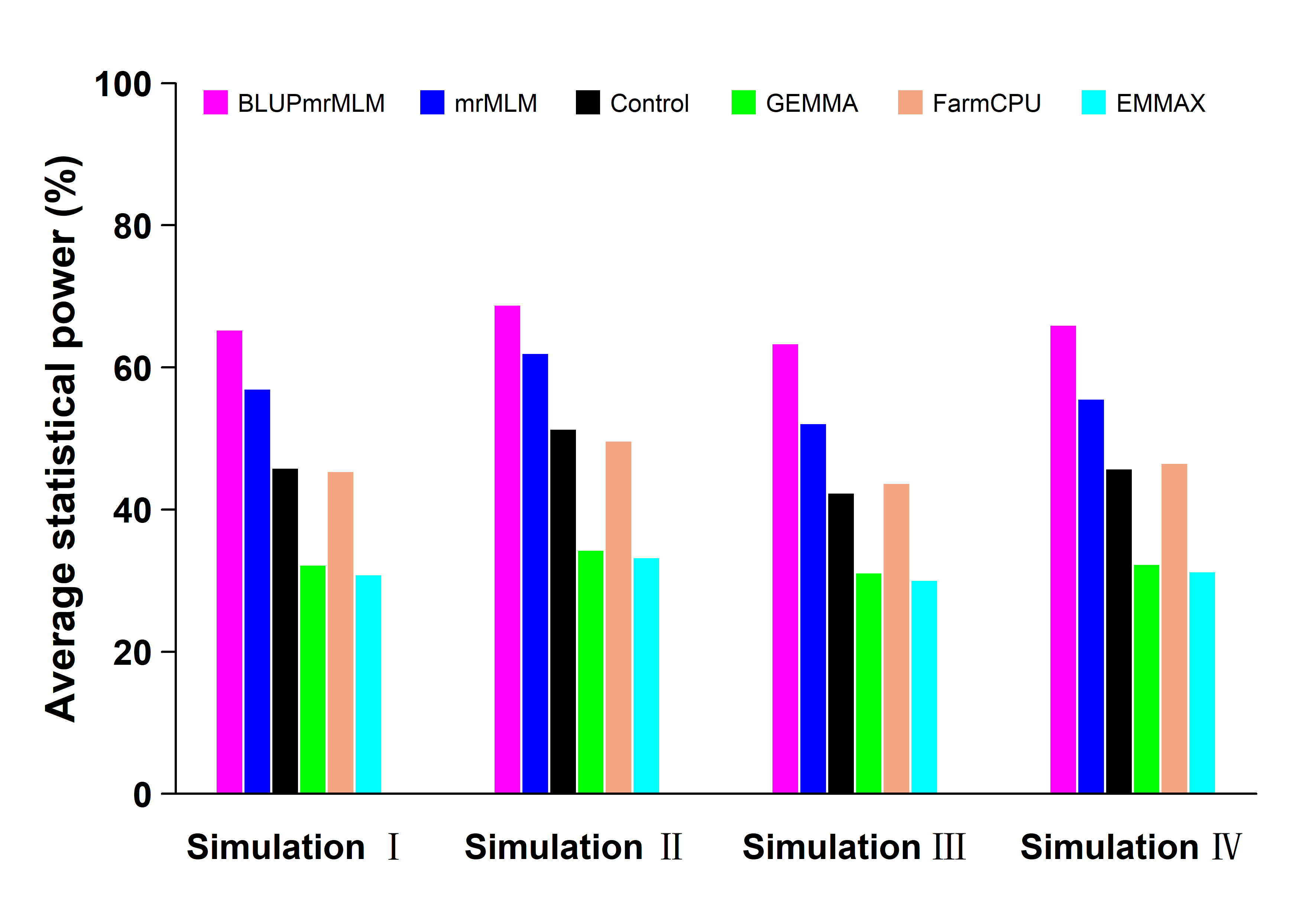

Supplement: qzae020_Supplementary_Data [file qzae020_supplementary_data.zip › Figure S2.tif]

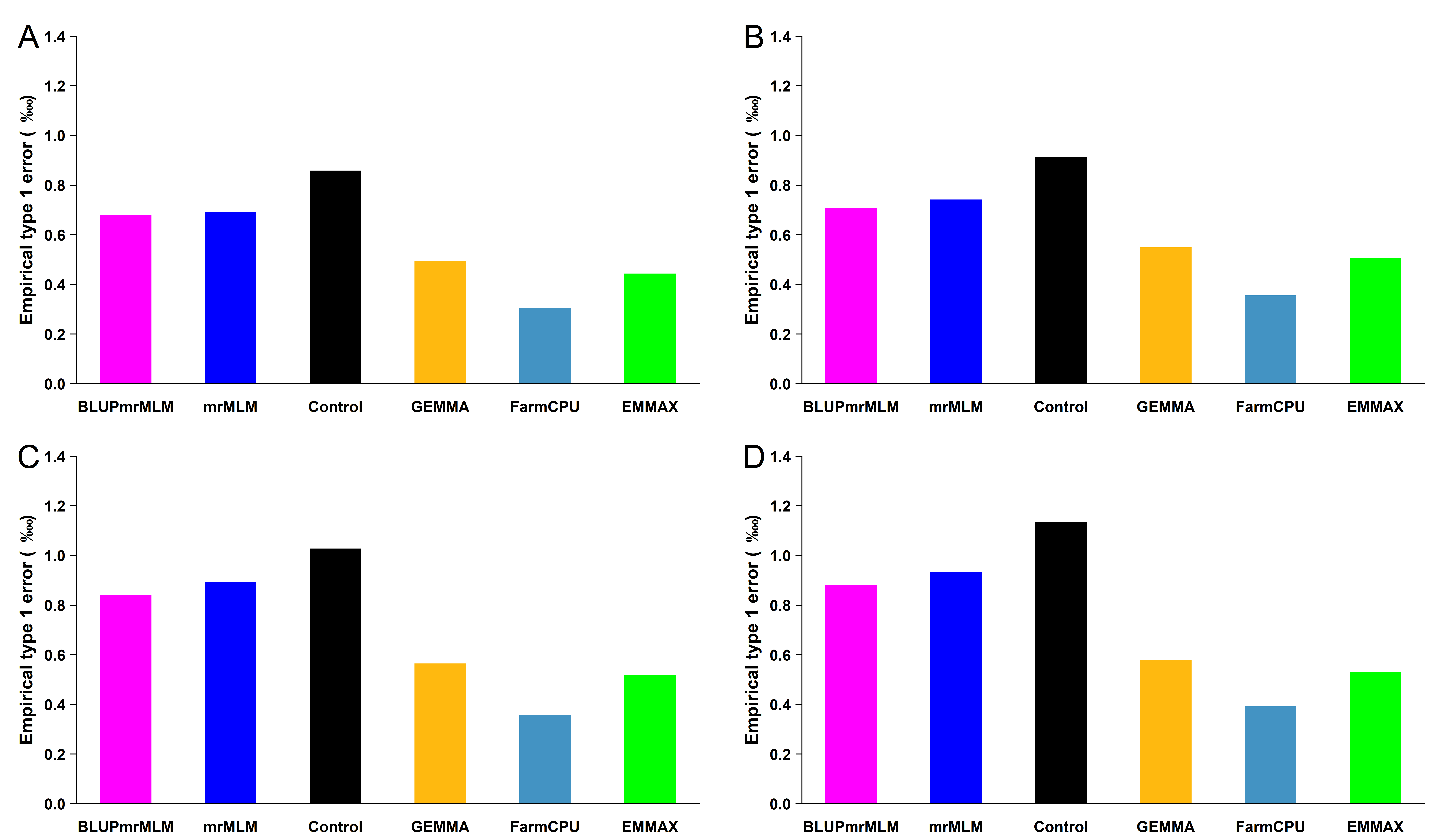

Supplement: qzae020_Supplementary_Data [file qzae020_supplementary_data.zip › Figure S5.tiff]
